# 2‑Substituted vs 2,9-Disubstituted Phenanthroline-Ni^II^-halides: Speciation Control and Structural Elucidation in Solution

**DOI:** 10.1021/acs.inorgchem.5c02704

**Published:** 2025-08-19

**Authors:** Ana Mateos-Calbet, Markus Leutzsch, Maurice van Gastel, Daniel J. SantaLucia, Josep Cornella

**Affiliations:** † 28314 Max-Planck-Institut für Kohlenforschung, Kaiser-Wilhelm-Platz 1, Mülheim an der Ruhr, 45470, Germany; ‡ 28313 Max Planck Institute for Chemical Energy Conversion, Stiftstraße 34−36, Mülheim an der Ruhr, 45470, Germany

## Abstract

Herein we study the structures of selected 2,9-disubstituted and 2-monosubstituted phenanthroline Ni^II^-dibromido and diiodido complexes via SC-XRD, SQUID magnetometry, UV–vis–NIR and DOSY NMR. In spite of the divergent speciation in solid state and in DMF solution, the Ni^II^-dibromido complexes of both mono- and disubstituted phenanthrolines converge to monomeric species in both THF and CDCl_3_. On the other hand, the nickel iodide complexes remain as monomers both in solid state and in solution, regardless of ligand substitution patterns.

## Graphical abstract

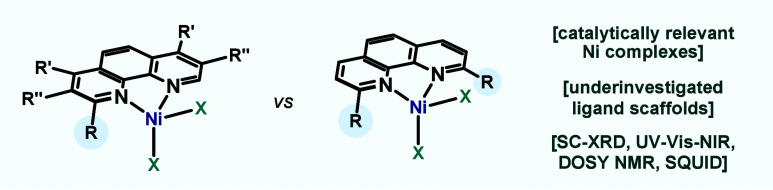

Bipyridine- and phenanthroline-derived ligands have been established as privileged scaffolds for nickel catalysis, where they are frequently used in combination with simple Ni^II^-halide salts.^1^ However, mechanistic investigations into such transformations are hampered by the propensity of Ni-polypyridyl species to speciate in solution. For example, a closer analysis on Ni^II^ complexes reveals their tendency to undergo solvent coordination;^2^ mono-, di- and oligomerizations;^3^ as well as ligand exchange processes leading to various complexes with different ligand-to-metal ratios^4,5^ and nickelates^6^ (Figure 1A). This rich dynamic behavior underscores that the commonly assumed 1:1 stoichiometry in precatalyst systems (comprising Ni^II^ salts and diamines) conceals a significant degree of complexity in the speciation of the actual active species. An approach that simplifies the solution speciation and permits mechanistic organometallic studies on Ni-polypyridyl complexes is to place substituents at the α-positions of the nitrogen atoms on the ligand (positions 2,9- for phenanthrolines and 2,2’- for bipyridines, here “di-substituted”).^7^ However, due to the paramagnetic nature of the complexes, their structures in solution remain speculative. The intermediate substitution pattern ligands, i.e. α-monosubstituted bipyridines and phenanthrolines, have also been shown to be the ligands of choice in certain Ni-catalyzed transformations (Figure 1B).^8,9^ However, data on their structures in solid state and solution have rarely been reported,^2b,7c,8e,10,11^ presumably daunted by the complicated behavior of the unsubstituted polypyridines. Here, we report our findings regarding the structures and solution behavior of a series of α-mono- and -disubstituted phenanthroline-Ni^II^-halide complexes, thus providing important structural information into catalytically relevant systems (Figure 1C).

**Figure 1:**
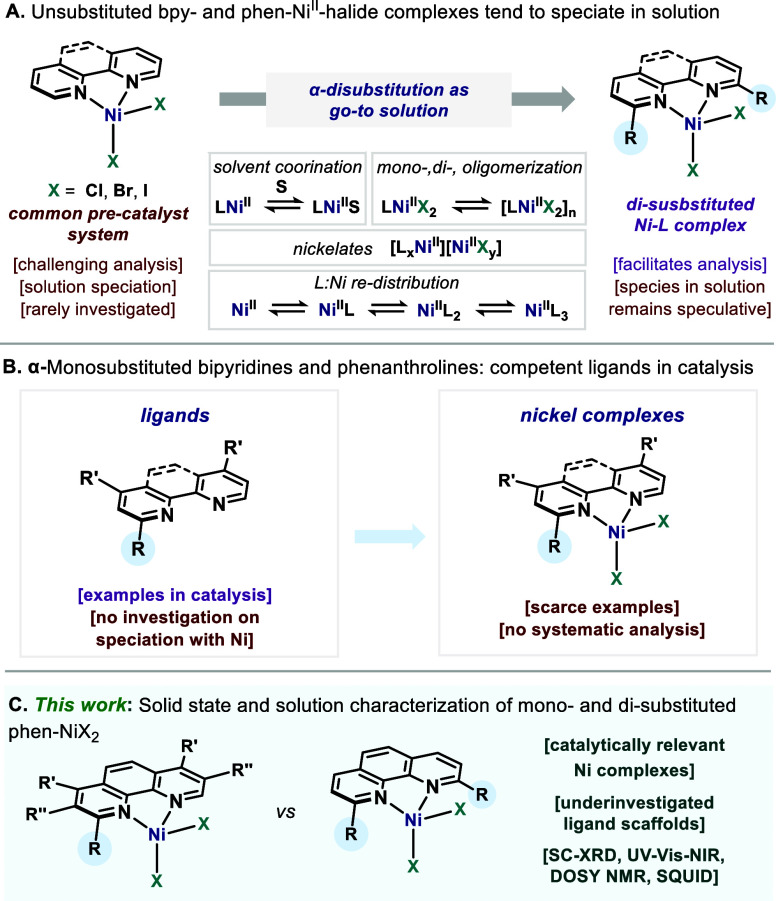
Bipyridine- and phenanthroline-Ni^II^-dihalide complexes in catalysis and coordination chemistry. (A) Unsubstituted and α-disubstituted bipyridine and phenanthroline Ni^II^-dihalides in solution. (B) α-Monosubstituted bipyridines and phenanthrolines in nickel catalysis and structural characterization of their Ni^II^-dihalide complexes. (C) The present speciation study of α-monosubstituted phenanthroline Ni^II^-dihalides compared to α-disubstituted ones.

We selected a representative set of 2- and 2,9-disubstituted phen ligands. Their Ni^II^-dibromido complexes were prepared via ligand exchange by sonication of NiBr_2_(DME) (1.0 equiv) with the corresponding ligand (1.0 equiv) in THF for ca. 14 h, affording complexes **1–5** (see SI for details). We were able to obtain crystals suitable for X-ray diffraction analysis for all complexes except for **5**, which exhibited high insolubility.^12^ Therefore, **5** was analyzed and characterized by elemental analysis (EA) and SQUID (superconducting quantum interference device) magnetometry to assess its nickel:ligand ratio and nuclearity in the solid state (Figure 2A). In all cases, the nickel:ligand ratio is 1:1. However, in line with literature precedents, in the solid state the 2,9-disubstituted ligands afforded monomeric complexes in all cases; while the 2-monosubstituted ones afforded dimeric species, linked through μ-Br bridges.^13,14^ In spite of the lack of structural X-ray data for **5**, the magnetometry data confirms a ferromagnetically coupled Ni^II^ dimer, suggesting a similar solid-state structure as **3** and **4**. This nuclearity partition had a clear effect on the appearance: all monomeric species exhibited a pink color in the solid-state, whereas the dimeric species consistently appeared yellow. This observation was further underscored through UV–vis–NIR spectroscopy, where two clear sets of spectra were recorded (pink and yellow traces in Figure 2B).^15^

**Figure 2:**
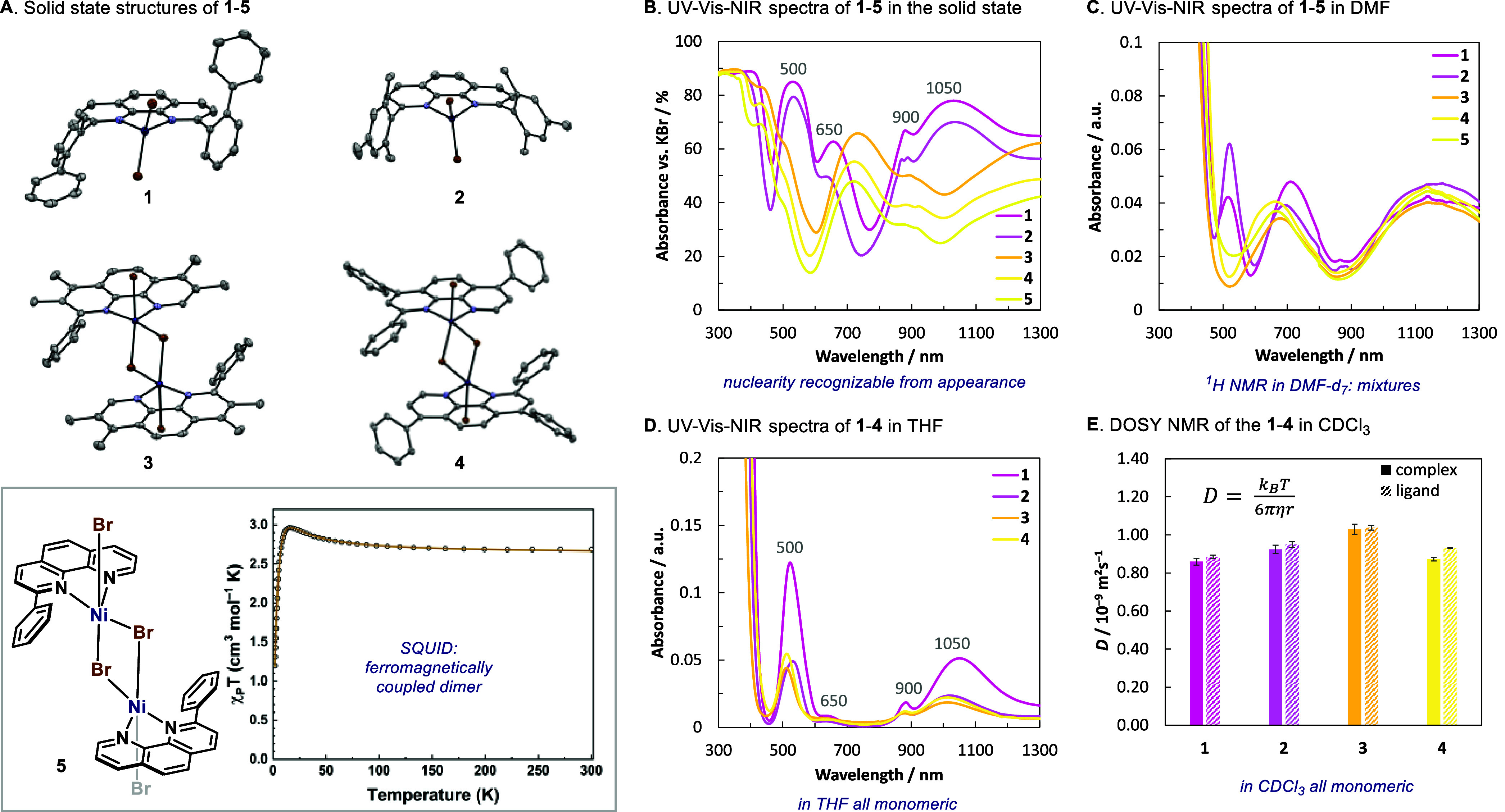
Solid state and solution behavior of complexes **1**–**5**. The colors chosen for the traces approximate the colors of the compounds in the solid state. (A) sc-XRD structures of complexes **1**–**4** (thermal ellipsoids at 50% probability) and SQUID magnetometry of complex **5**. (B) Absorption spectra of complexes **1**–**5** in the solid state. (C) Absorption spectra of complexes **1**–**5** in DMF solution. (D) Absorption spectra of complexes **1**–**4** in THF solution. (E) Diffusion coefficients of complexes **1**–**4** and their free ligands in CDCl_3_ solution, as determined by DOSY NMR.

When complexes **1–5** were dissolved in DMF (a common solvent utilized in Ni-catalysis), the spectra remained in two distinct groups as in the solid state. Yet, the maxima in absorbance shifted in most cases. In agreement with this observation, the ^1^H NMR spectra in DMF-*d*_7_ revealed the formation of mixtures for all complexes (see SI for details).^4a^ For the disubstituted phen-Ni complexes, partial decoordination of the ligand was observed, and, in all cases (with the exception of **2**), two paramagnetic species were detected that could not be identified. The hypsochromic shift of the absorption maxima of **3**–**5** in DMF respect to their spectra in the solid state,^4,6c,16^ as well as the crystallization of the L_2_NiBr_2_ analog of **5** from a solution of **5** in DMF (see SI for details), points to ligand exchange processes in those solutions, as observed in the cited reports.

When ^1^H NMR spectra were recorded in CDCl_3_ and THF-*d*_8_, simpler spectra were obtained, with only one paramagnetic species present (see details in SI; **5** could not be analyzed in these solvents due to its low solubility). Surprisingly, in these solvents all solutions were pink, reminiscent of the color of the monomers in the solid state. Interestingly, the UV–vis–NIR spectra of the complexes in THF converged, and all presented absorption maxima at approximately the same wavelengths as the monomeric solids (Figure 2D). This is indicative of the same electronic and geometric structure for all complexes analyzed, meaning that *we can unequivocally identify the speciation of these complexes as LNiBr*_2_ *in THF solution*. The simple speciation in CDCl_3_ also enabled their analysis by DOSY NMR in spite of their paramagnetism. All complexes were measured in CDCl_3_ to have very similar self-diffusion coefficients as their ligands (Figure 2E). Based on the Stokes–Einstein-Sutherland equation, in which the diffusion coefficient, *D*, is inversely proportional to the Stokes radius, *r*, of the solvate particles (Figure 2E; *T*, absolute temperature; *k*_*B*_, Boltzmann constant; η, dynamic viscosity), the diffusion coefficient of dimeric complexes should roughly be half the one of their free ligands. However, this is not observed, thus suggesting these complexes exist predominantly as monomeric species in CDCl_3_ as well.

Early coordination studies on neocuproine- and bisquinolyl-Ni dihalide complexes by Butcher et al. suggest that a monomerization trend following Cl < Br < I occurs in the solid state.^17^ Inspired by these precedents, we prepared the iodido analogs of complexes **1**, **3**, and **4**. Complexes **6**–**8** were prepared in a similar manner as the bromido analogs but using NiI_2_ (1.0 equiv) instead. Indeed, both complexes bearing 2,9-substitution as well as only 2-substituted phenanthrolines were *all monomeric in the solid state*, as analyzed by SC-XRD (Figure 3A).^18^ The three complexes were measured to have analogous UV–vis–NIR spectra in the solid state (Figure 3B), which were conserved in THF solution (Figure 3C). Furthermore, their ^1^H NMR spectra in THF-*d*_8_, and CDCl_3_ accounted for a single paramagnetic species. Taken together, this indicates that the iodide analogs also exist as monomeric LNiI_2_ species in THF solutions.

**Figure 3:**
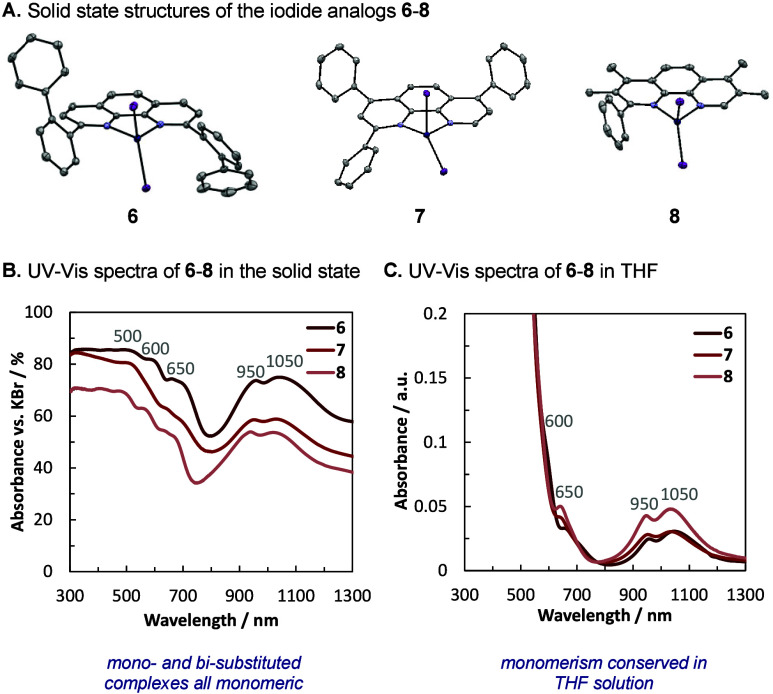
Solid state and solution behavior of 2- and 2,9-substituted phenanthroline-NiI_2_ complexes. The colors chosen for the spectra lines approximate to the appearance of the complexes in the solid state. (A) sc-XRD structures of complexes **6**–**8** (thermal ellipsoids at 50% probability). (B) Absorption spectra of complexes **6**–**8** in the solid state. (C) Absorption spectra of complexes **6**–**8** in THF solution.

In summary, we have prepared and analyzed a series of 2- and 2,9-substituted phenanthroline Ni^II^ dibromido and diiodido complexes, and analyzed their coordination geometry in the solid state as well as their speciation in solution (Figure 4). In the solid state, the 2,9-substituted phenanthroline Ni^II^ complexes are monomeric and pink, while the 2-substituted ones form dimers that appear yellow in color. In DMF, all studied complexes form mixtures, whereas in CDCl_3_ and THF the complexes appear as a single monomeric LNiBr_2_ species. On the contrary, Ni^II^ diiodido analogs bearing phenanthrolines with any of the substitution patterns (2- or 2,9-) were monomeric in the solid state, and retained their monomeric form in THF solution. The study presented here benefited from a cooperative analysis through the combination of SC-XRD, NMR and DOSY NMR, EA, SQUID magnetometry, and UV–vis–NIR analyses.

**Figure 4:**
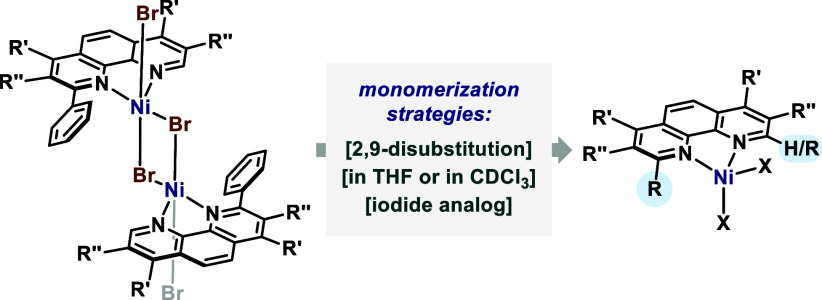
Overview of insights gained in this work.

Given the wide range of catalytic applications reported for some of the phenanthroline–Ni^II^ complexes reported herein, this work aims to serve as an informative tool for understanding the intricate speciation behavior of apparently simple Ni^II^ halide–phenanthroline systems. Speciation has implications in the coordination environment, which in turn, have a profound effect in their redox properties thus affecting reductive couplings or metallaphotoredox processes. These findings highlight how speciation at the Ni^II^ precatalyst level can be significantly influenced by factors such as counterions, solvents, and sterical encumberance at the Ni^II^-center. In addition to emphasizing these effects, this study provides experimental tools and insights to probe the solution behavior of these complexes. We believe this information will assist practitioners in making more informed decisions when designing and optimizing new Ni-based catalysts.

## Supplementary Material



## Notes

Experimental and characterization details, including ^1^H NMR spectra, UV–vis–NIR spectra and calculations, crystallographic data and computational methods (PDF)
