# Supplementary material for: 2‑Substituted vs 2,9-Disubstituted Phenanthroline-NiII-halides: Speciation Control and Structural Elucidation in Solution
Source: Inorg Chem. 2025 Aug 19;64(34):17093–7. doi: 10.1021/acs.inorgchem.5c02704 (PMC12406190; doi:10.1021/acs.inorgchem.5c02704)
Supplement: Supplementary file 1 [file ic5c02704_si_001.pdf]

Supporting Information

2-Substituted vs. 2,9-Disubstituted Phenanthroline-Ni^{II}-halides: Speciation Control and Structural Elucidation in Solution

Ana Mateos-Calbet,[1] Markus Leutzsch,[1] Maurice van Gastel,[1] Daniel J. SantaLucia,[2] and Josep Cornella[1]*

[1]Max-Planck-Institut für Kohlenforschung, Kaiser-Wilhelm-Platz 1, Mülheim an der Ruhr, 45470, Germany

[2]Max Planck Institute for Chemical Energy Conversion, Stiftstraße 34–36, Mülheim an der Ruhr, 45470, Germany

*Corresponding author: cornella@kofo.mpg.de

Table of Contents

1. **General Information** 3
2. **Synthesis of Nickel Complexes** 4
   - **Complex 1** 4
   - **Complex 2** 7
   - **Complex 3** 9
   - **Complex 4** 12
   - **Complex 5** 15
   - **Complex 6** 19
   - **Complex 7** 20
   - **Complex 8** 22
3. **Collected UV-Vis-NIR spectra and simulations** 24
   - **UV-Vis-NIR in the solid state** 24
   - **UV-Vis-NIR in DMF** 25
   - **UV-Vis-NIR in THF** 26
   - **Calculated Spectra and Selected Transitions** 27
4. **DOSY NMR of complexes 1-4** 36
   - **2D DOSY spectra of complexes 1-4** 39
   - **2D DOSY spectra of ligands L1-L4** 41
5. **Crystallographic Data** 44
   - **Single Crystal structure of 2** 44
   - **Single Crystal structure of 3** 49
   - **Single Crystal structure of 4** 53
   - **Single Crystal structure of 6** 58
   - **Single Crystal structure of 7** 63
   - **Single Crystal structure of 8** 68
   - **Single Crystal structure of S1** 72
6. **NMR Spectra** 78
7. **References** 84

1. General Information

Unless otherwise stated, all manipulations were performed under argon using standard Schlenk line techniques or in an MBraun argon-filled glove box.

Chemicals: THF-d_8 was purchased from Eurisotop, degassed by repeated freeze-pump-thaw cycles, distilled from the proper drying agents, and stored over 4 Å molecular sieves. 4 Å molecular sieves were activated at 180 °C under high vacuum (1×10^{-3} bar) for 18 h. Anhydrous n-pentane, THF, Et_2O and toluene were distilled from appropriate drying agents and were transferred under argon. Unless otherwise noted, all reagents were obtained from commercial suppliers and used without further purification.

The synthesis and analysis of ligands 2,9-di([1,1'-biphenyl]-2-yl)-1,10-phenanthroline, 2,9-dimesityl-1,10-phenanthroline, 3,4,7,8-tetramethyl-2-phenyl-1,10-phenanthroline, 2,4,7-triphenyl-1,10-phenanthroline, 2-phenyl-1,10-phenanthroline, as well as of complex **1** were prepared according to the literature procedure.[1]

Instruments: NMR spectra were recorded using a Bruker AVIII HD 300 MHz, Bruker Advance III HD 400 MHz, or Bruker AVIII 500 MHz. ^{1}H and ^{13}C chemical shifts (δ) are given in ppm relative to tetramethylsilane (TMS), coupling constants (*J*) in Hz. The solvent signals were used as references and the chemical shifts converted to the TMS scale, for ^{1}H NMR: $CDCl_3$, δ 7.26 ppm; THF-d_8, δ 1.73 ppm; DMF-d_7, δ 2.75 ppm. ^{1}H NMR spectra of the paramagnetic complexes were processed using a multipoint baseline correction with the segments algorithm in MestReNova. Multiplicities are described as s = singlet, br s = broad singlet, d = doublet, t = triplet, q = quartet, dd = doublet of doublets, td = triplet of doublets, m = multiplet. Elemental analysis was conducted at Mikroanalytisches Labor Kolbe. Variable-temperature direct current paramagnetic susceptibility and variable-temperature/-field (VTVH) magnetization data was measured in the Joint Workspace of the Max Planck Institute for Chemical Energy Conversion (MPI-CEC) and the Max-Planck-Institut für Kohlenforschung (MPI-KoFo) with a MPMS 3 Quantum Design SQUID magnetometer. Magnetometry data were processed using mpView version 1.4.6 (written by Dr. Eckhard Bill and available by email to: daniel.santalucia@cec.mpg.de). The susceptibility and VTVH data were modeled simultaneously using JulX20 version 1.4 (written by Dr. Eckhard Bill and available by email to: daniel.santalucia@cec.mpg.de). All UV-VIS spectra have been measured from 1800 nm to 200 nm in suprasil quartz cuvettes using an Agilent Cary6000i UV-VIS-NiR spectrometer.

2. Synthesis of Nickel Complexes

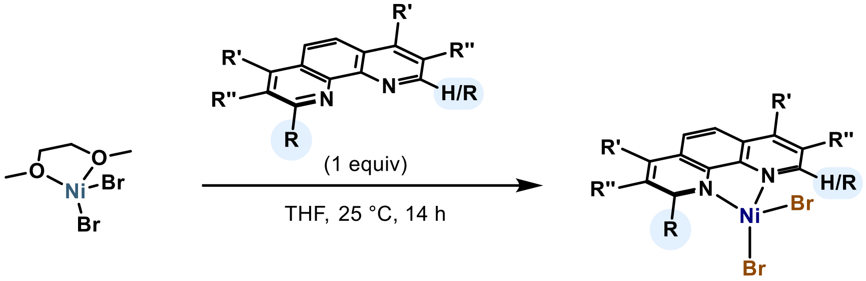

General Procedure A (GPA): Complexes **1-5** were prepared following a reported procedure.[1] $NiBr_2$(glyme) (1 equiv.), ligand (1 equiv.), and THF were added into a 250 mL Schlenk flask under argon. The mixture was stirred for 30 min at 25 °C and sonicated for 14 h. It was concentrated to a volume of 10 mL under vacuum. Et_2O (150 mL) was added, the suspension stirred for a *ca.*1 min, and the supernatant removed by decantation. The solid was washed with Et_2O (5 × 150 mL) and dried under vacuum to give complexes **1-5** as pink or yellow solids.

General Procedure B (GPB): Complexes **6-8** were prepared following a slightly modified procedure.[1] NiI_2 (1 equiv.), ligand (1 equiv.), and THF were added into a 250 mL Schlenk flask under argon. The mixture was stirred for 30 min at 25 °C and sonicated for 14 h. The solvent was then removed under reduced pressure under argon and dried under vacuum to give complexes **6**-**8** as brown solids.

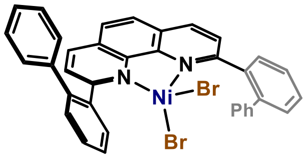

Complex 1

The preparation and analysis of complex **1** has been reported in a previous publication.[1] SC-XRD analysis revealed a monomeric structure, and the ^{1}H NMR spectra in $CDCl_3$ and THF-d_8 showed a single paramagnetic species.

^{1}H NMR in DMF-d_7

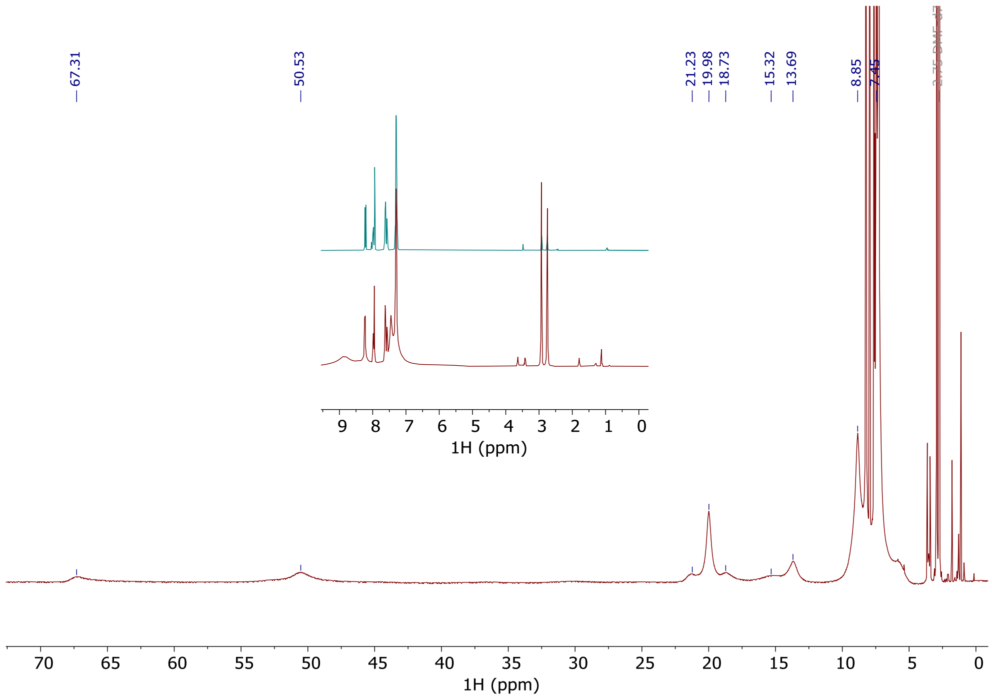

*Figure S1: ^{1}H NMR (400 MHz, 25 °C) of complex **1** in DMF-d_7 (maroon trace). Zoom-in of the diamagnetic region, ^{1}H NMR of the free ligand in DMF-d_7 included for comparison (turquoise trace).*

In the ^{1}H NMR of **1** in DMF-d_7 there seems to be more than one paramagnetic species, substantial amounts of free ligand are also observable. The formyl signal of the DMF-d_7 is no longer observable. The broadness of the signals prevented their integration.

UV-Vis-NIR

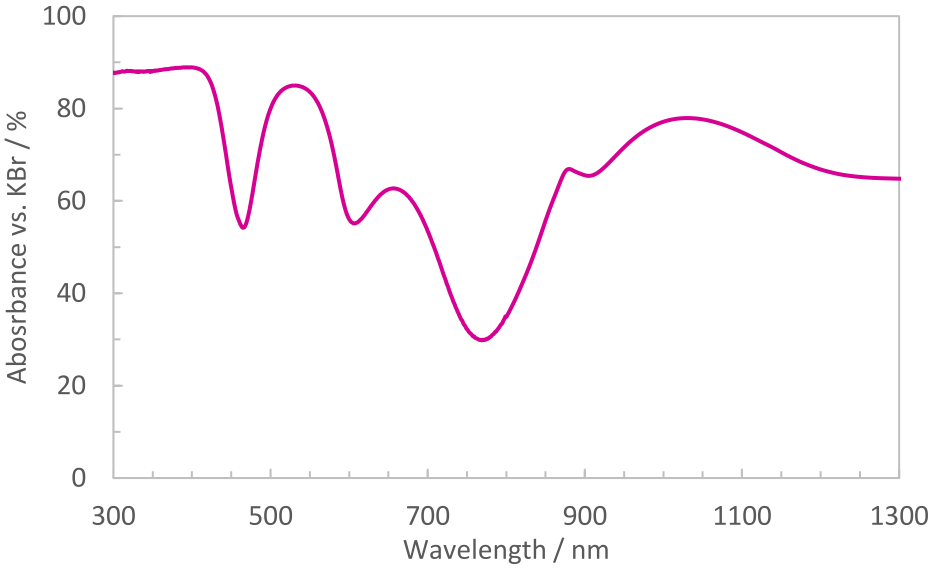

*Figure S2: UV-Vis-NIR spectrum of **1** in the solid state vs. KBr.*

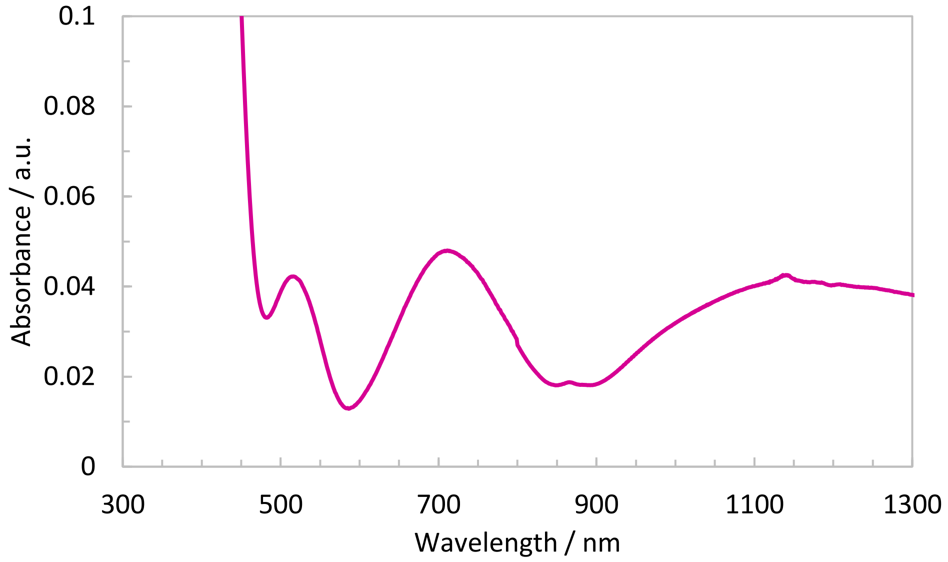

*Figure S3: UV-Vis-NIR of **1** in DMF solution (30 mM).*

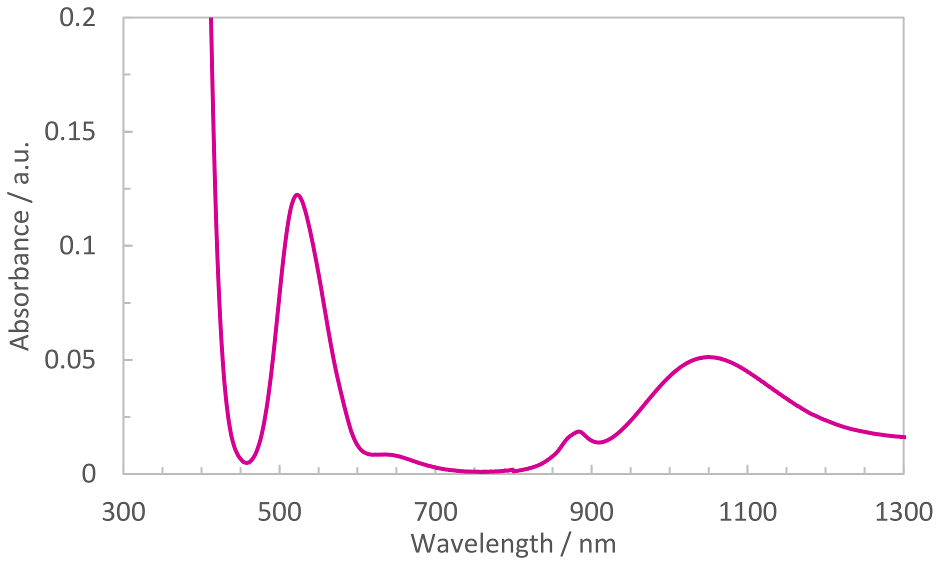

*Figure S4: UV-Vis-NIR of **1** in THF solution.*

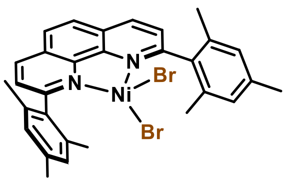

Complex 2

Following the **GPA**, $NiBr_2$(glyme) (296 mg, 0.960 mmol, 1 equiv.), 2,9-dimesityl-1,10-phenanthroline (400 mg, 0.960 mmol, 1 equiv.), and THF (100 mL) were employed. Complex **2** was obtained in 91% yield (556 mg) as a pink powder. Crystals suitable for SC-XRD analysis were obtained *via* benzene vapor diffusion into a $CDCl_3$ solution of **2**, and revealed a monomeric structure (see crystallographic data section).

^{1}H NMR (500 MHz, $CDCl_3$, 25 °C): δ 71.89 (s, 2H), 25.43 (s, 2H), 24.36 (s, 2H), 8.78 (s, 4H), 4.96 (s, 6H), 4.34 (s, 12H).

^{1}H NMR (400 MHz, THF-*d*$_8$, 25 °C): δ 70.85 (s, 2H), 25.03 (s, 2H), 24.18 (s, 2H), 8.53 (s, 4H), 4.54 (s, 6H), 4.05 (s, 12H).

The spectroscopic data was in agreement with those reported in the literature.[2]

^{1}H NMR in DMF-*d*$_7$

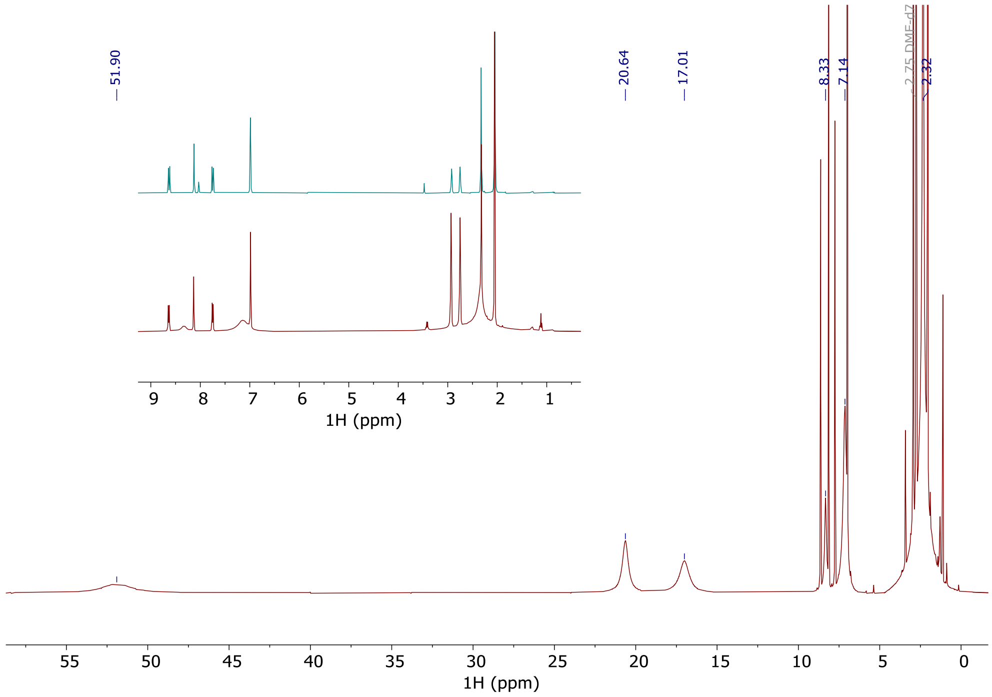

*Figure S5: ^{1}H NMR (400 MHz, 25 °C) of complex **2** in DMF-d$_7$ (maroon trace). Zoom-in of the diamagnetic region, ^{1}H NMR of the free ligand in DMF-d$_7$ included for comparison (turquoise trace).*

In the ^{1}H NMR of **2** in DMF-*d*$_7$ there seems to be one set of signals corresponding to a paramagnetic species, substantial amounts of free ligand are also observable. The formyl signal

of the DMF-d_7 appears shifted and broadened, from 8.03 ppm to 8.33 ppm. The broadness of the signals prevented their integration.

UV-Vis-NIR

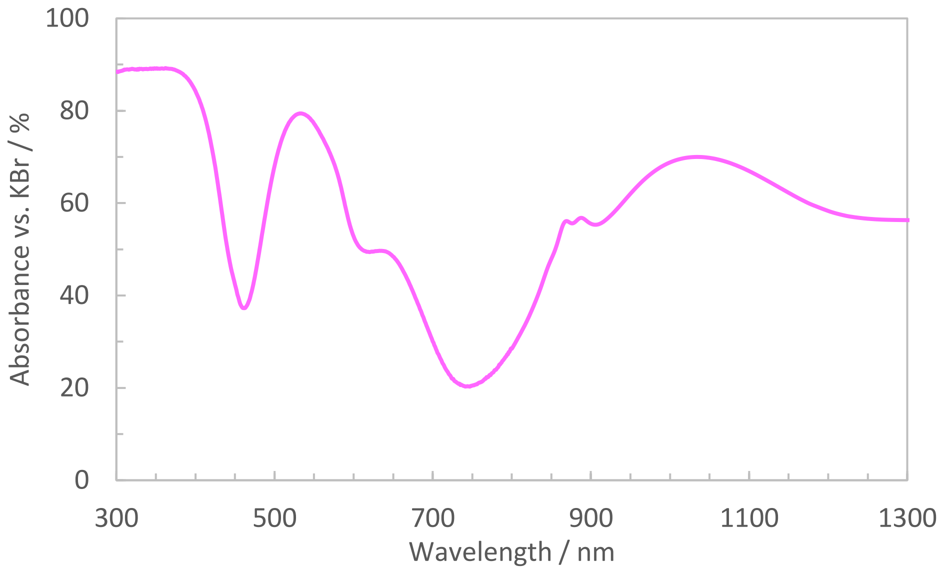

*Figure S6: UV-Vis-NIR spectrum of **2** in the solid state vs. KBr.*

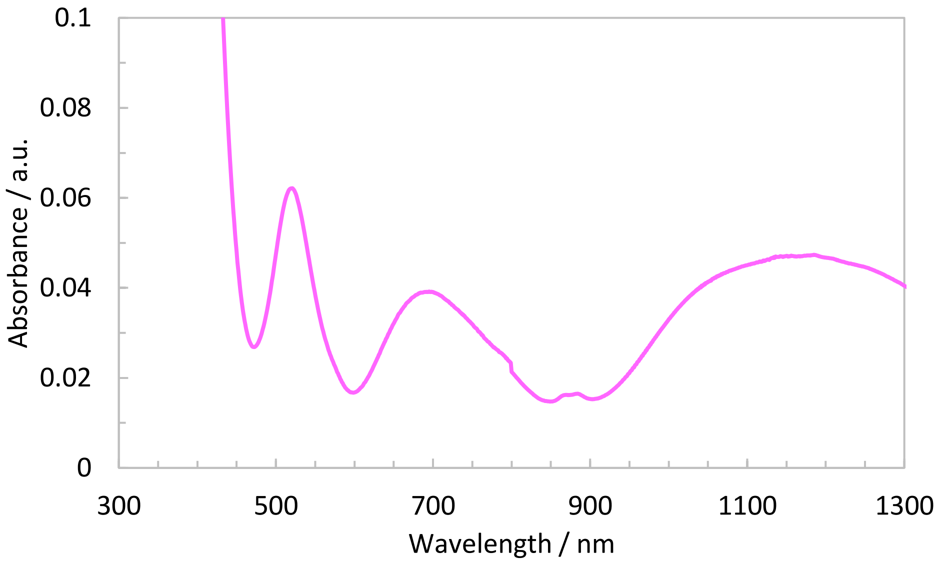

*Figure S7: UV-Vis-NIR of **2** in DMF solution (30 mM).*

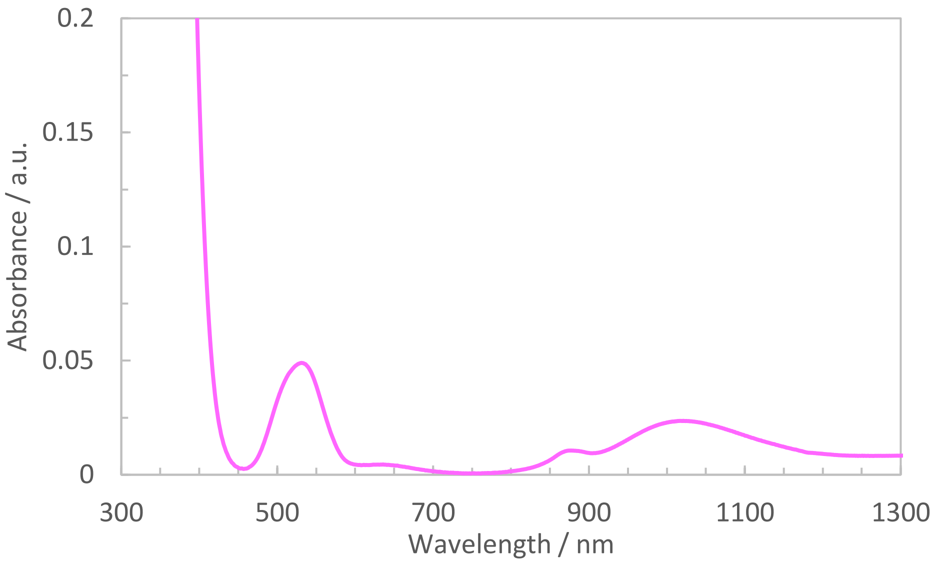

*Figure S8: UV-Vis-NIR of **2** in THF solution.*

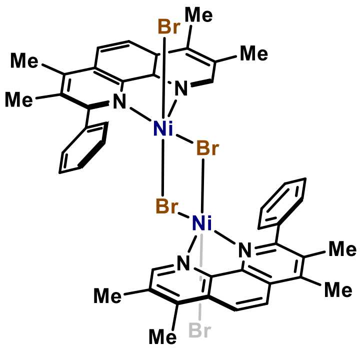

Complex 3

Following the **GPA**, $NiBr_2$(glyme) (296 mg, 0.960 mmol, 1 equiv.), 3,4,7,8-tetramethyl-2-phenyl-1,10-phenanthroline (300 mg, 0.960 mmol, 1 equiv.), and THF (100 mL) were employed. Complex **3** was obtained in 97% yield (493 mg) as a yellow powder. Crystals suitable for SC-XRD analysis were obtained *via* benzene vapor diffusion into a $CDCl_3$ solution of **3**, and revealed a dimeric structure (see crystallographic data section).

*NMR spectra of complexes featuring this ligand (**3** and **8**) always miss two hydrogen atoms. In the crystal structures of these complexes, no abnormalities can be observed in the ligand (vide infra). Therefore, missing signals can be attributed to the paramagnetic nature of the complexes.*

^{1}H NMR (500 MHz, $CDCl_3$, 25 °C): δ 32.86 (s, 1H), 26.40 (s, 2H), 16.35 (s, 3H), 15.59 (s, 3H), 10.91 (s, 1H), 7.82 (s, 2H), -10.22 (s, 3H), -11.08 (s, 3H).

^{1}H NMR (400 MHz, THF-d_8, 25 °C): δ 32.20 (s, 1H), 25.83 (s, 1H), 24.77 (s, 1H), 15.90 (s, 3H), 14.79 (s, 3H), 10.45 (s, 1H), 7.93 (s, 2H), -10.60 (s, 3H), -11.57 (s, 3H).

^{1}H NMR in DMF-d_7

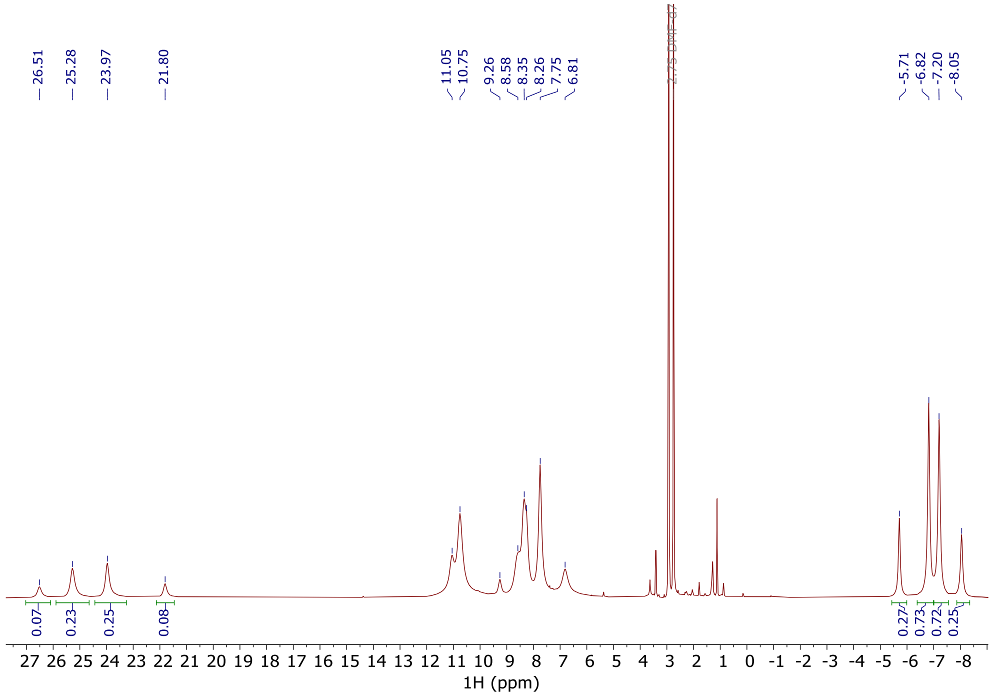

*Figure S9: ^{1}H NMR (400 MHz, 25 °C) of complex **3** in DMF-d_7*. Due to overlapping, integration was only employed to determine the approximate ratio of the observed species (75 : 25).

In the ^{1}H NMR spectrum of **3** in DMF-d_7 two paramagnetic species in an approximate ratio of 75 : 25 can be observed; free ligand is not observed.

UV-Vis-NIR

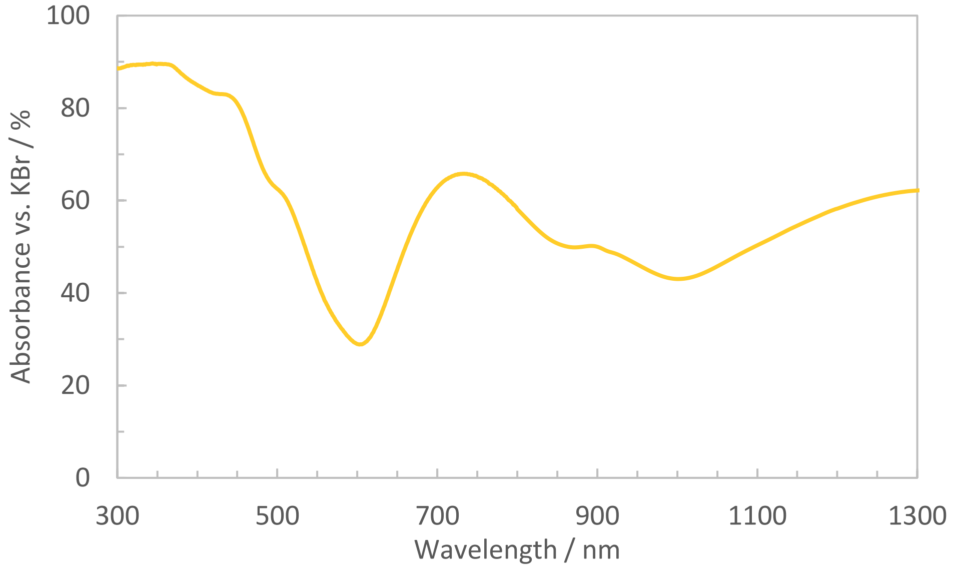

*Figure S10: UV-Vis-NIR spectrum of **3** in the solid state vs. KBr.*

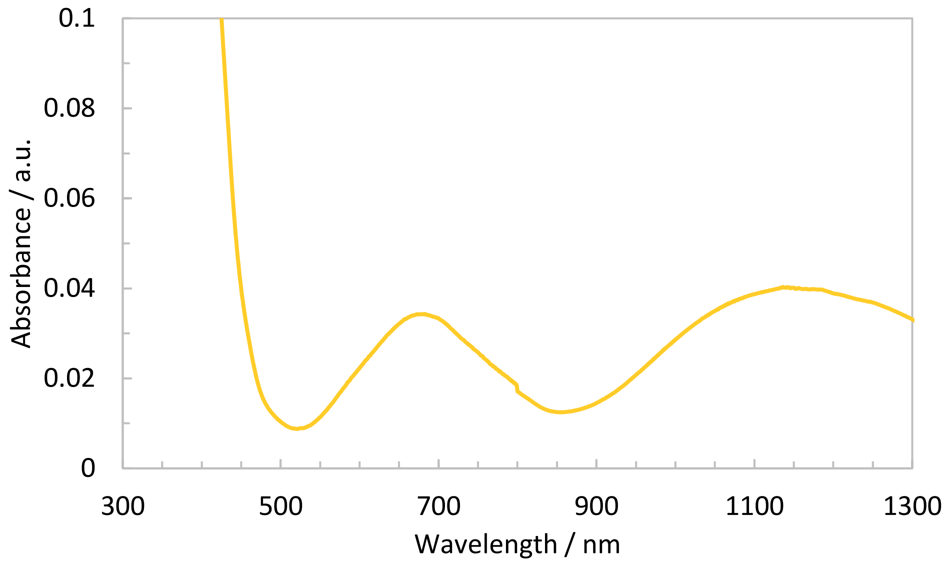

*Figure S11: UV-Vis-NIR of **3** in DMF solution (30 mM).*

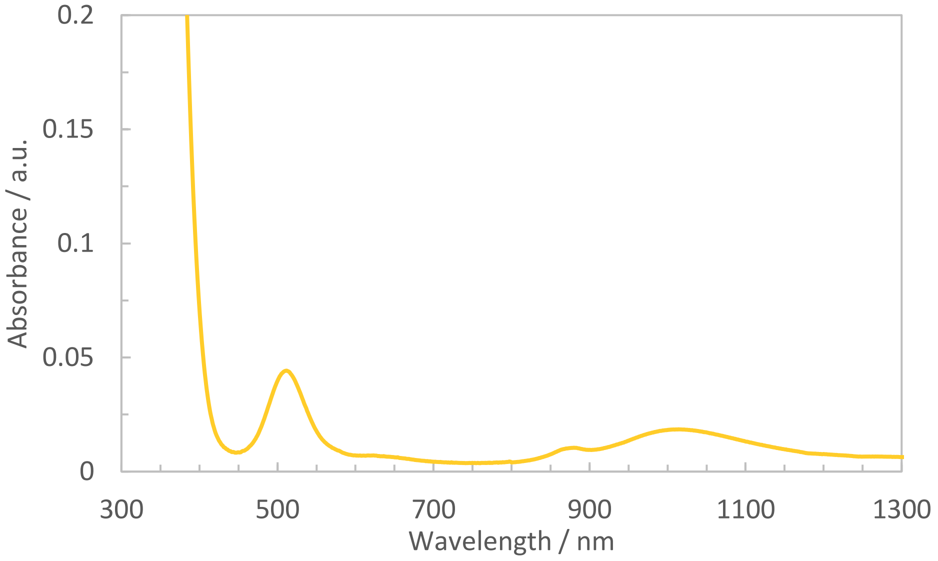

*Figure S12: UV-Vis-NIR of **3** in THF solution.*

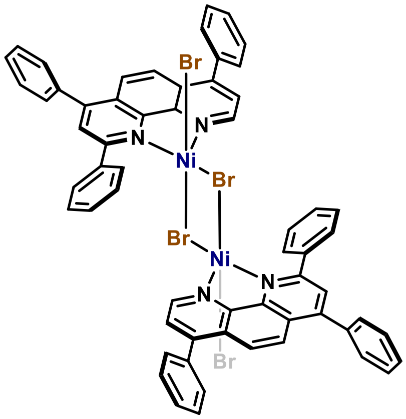

Complex 4

Following the **GPA**, $NiBr_2$(glyme) (529 mg, 1.71 mmol, 1 equiv.), 2,4,7-triphenyl-1,10-phenanthroline (700 mg, 1.71 mmol, 1 equiv.), and THF (150 mL) were employed. Complex **4** was obtained in 96% yield (1.03 g) as a yellow powder. Crystals suitable for SC-XRD analysis were obtained *via* pentane vapor diffusion into a THF solution of **4**, and revealed a dimeric structure (see crystallographic data section).

^{1}H NMR (400 MHz, $CDCl_3$, 25 °C): δ 75.12 (s, 1H), 70.33 (s, 1H), 44.98 (s, 1H), 32.61 (s, 1H), 25.28 (s, 1H), 13.36 (s, 1H), 9.98 (d, J = 7.4 Hz, 2H), 8.98 (t, J = 7.5 Hz, 1H), 8.48 (d, J = 7.5 Hz, 2H), 8.31 (t, J = 7.5 Hz, 1H), 7.41 (t, J = 6.8 Hz, 2H), 7.10 (t, J = 7.2 Hz, 4H), 1.75 (s, 2H).

^{1}H NMR (400 MHz, THF-d_8, 25 °C): δ 73.45 (s, 1H), 68.96 (s, 1H), 43.83 (s, 1H), 32.63 (s, 1H), 24.86 (s, 1H), 13.42 (s, 1H), 10.39 (d, J = 7.2 Hz, 2H), 9.08 (t, J = 7.4 Hz, 1H), 8.40 (d, J = 8.3 Hz, 2H), 8.23 (t, J = 7.7 Hz, 1H), 7.91 (s, 2H), 7.47 (t, J = 7.2 Hz, 2H), 6.94 (t, J = 7.4 Hz, 2H), 1.12 (s, 3H).

^{1}H NMR in DMF-d_7

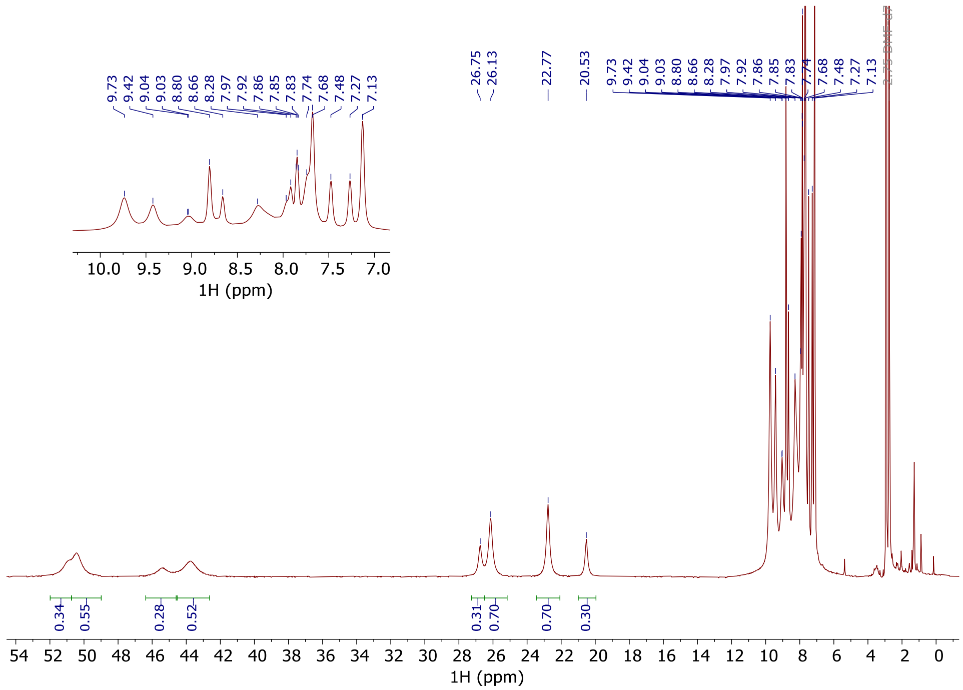

*Figure S13: ^{1}H NMR (400 MHz, 25 °C) of compound **4** in DMF-d$_7$. Due to overlapping, integration was only employed to determine the approximate ratio of the observed species (70 : 30).*

In the ^{1}H NMR spectrum of **4** in DMF-d_7 two paramagnetic species in an approximate ratio of 70 : 30 can be observed; free ligand is not observed.

UV-Vis-NIR

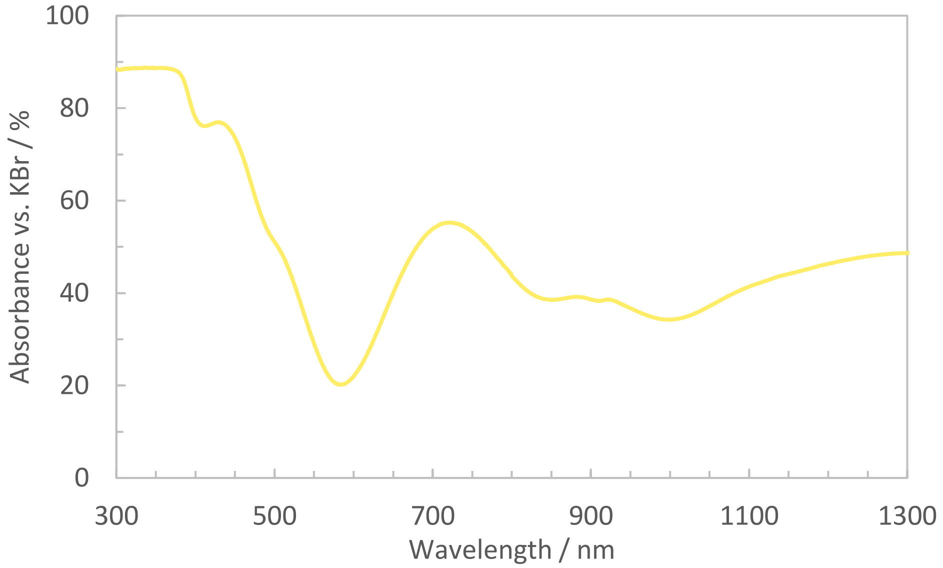

*Figure S14: UV-Vis-NIR spectrum of **4** in the solid state vs. KBr.*

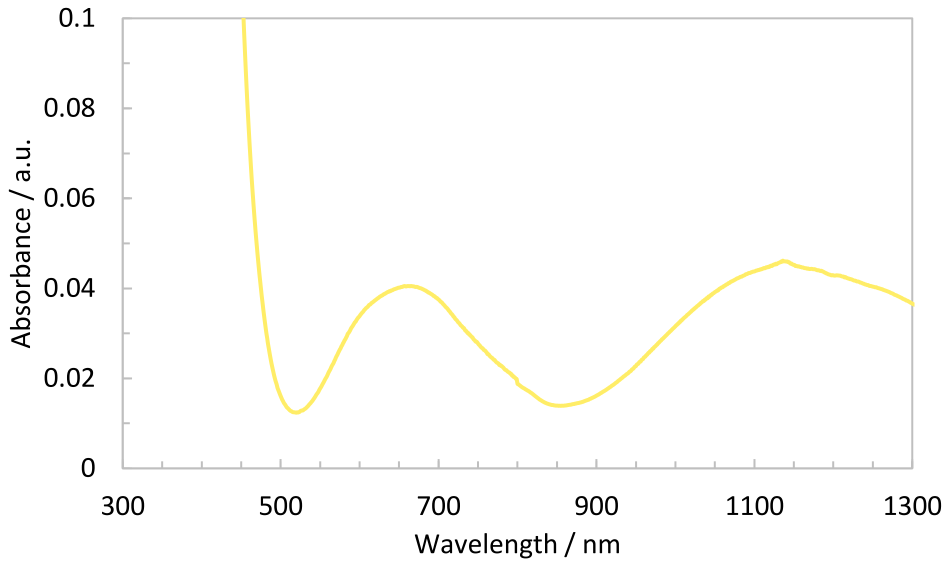

*Figure S15: UV-Vis-NIR of **4** in DMF solution (30 mM).*

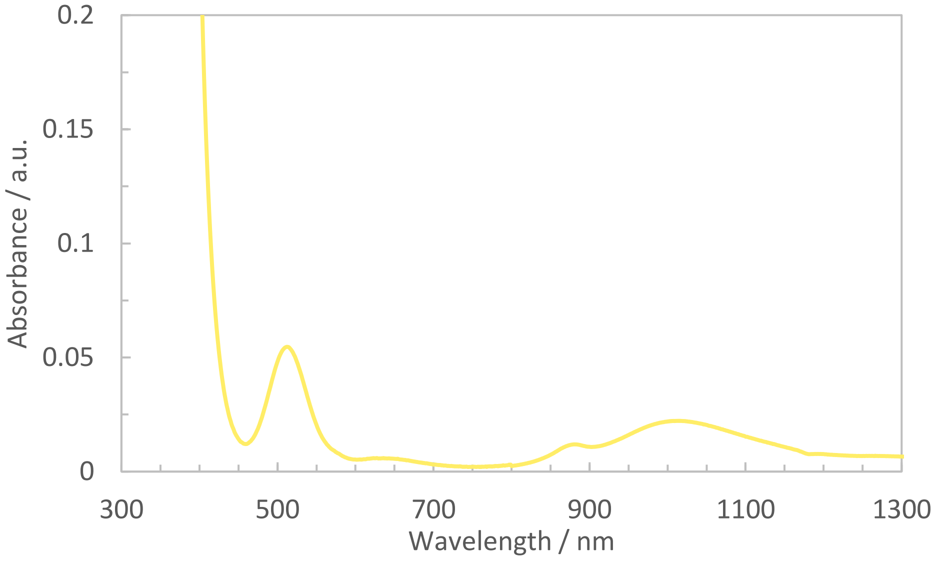

*Figure S16: UV-Vis-NIR of **4** in THF solution.*

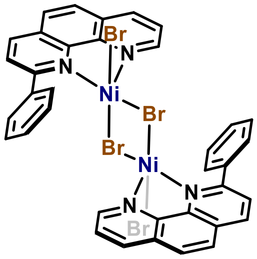

Complex 5

Following the **GPA**, $NiBr_2$(glyme) (903 mg, 2.93 mmol, 1 equiv.), 2-phenyl-1,10-phenanthroline (750 mg, 2.93 mmol, 1 equiv.), and THF (100 mL) were employed. Complex **5** was obtained in 90% yield (1.25 g) as a yellow powder. In spite of numerous attempts, the limited solubility of **5** precluded its crystallization. For this reason, elemental analysis (EA) and SQUID (superconducting quantum interference device) magnetometry were employed to identify the solid state structure of this complex.

EA calc. for $C_{36}H_{24}Br_4N_4Ni_2$: C 45.53, H 2.55, N 5.90, Ni 12.36; found: C 45.41, H 2.58, N 5.86, Ni 12.31.

Magnetometry (SQUID)

Approximately 15 mg of the complex was measured in a gel capsule sample holder. The diamagnetic susceptibilities of the gel capsule and sample holder were accounted for by measuring the empty holder under the same conditions, and subsequently the diamagnetism was subtracted during data processing. Experimental data were corrected for intrinsic diamagnetism using the following relation: $\chi_D = -M_W/2 \bullet 10^{-6}\ \mathrm{cm^3\ mol^{-1}} = -0.000474809\ \mathrm{cm^3\ mol^{-1}}$.

The sample was cooled to 2 K without an applied magnetic field. The magnetic moment was then measured in logarithmic increments up to 300 K in a 1000 Oe magnetic field. VTVH data were collected isofields of 10, 40, and 70 kOe while increasing the temperature from 2 to 300 K in even-spaced $1/T$ increments. Data were modeled with the following spin-Hamiltonian:

$$\hat{H} = \mu_B \overrightarrow{S_1}\, \overleftrightarrow{\boldsymbol{g_1}}\, \vec{B} + \mu_B \overrightarrow{S_2}\, \overleftrightarrow{\boldsymbol{g_2}}\, \vec{B} + D_1\left[\vec{S}_{z\,1}{}^2 - \frac{1}{3}S_1(S_1+1)\right] + E_1\left[\vec{S}_{x\,1}{}^2 - \vec{S}_{y\,1}{}^2\right] + D_2\left[\vec{S}_{z\,2}{}^2 - \frac{1}{3}S_2(S_2+1)\right] + E_2\left[\vec{S}_{x\,2}{}^2 - \vec{S}_{y\,2}{}^2\right] - 2J_{12}\overrightarrow{S_1}\,\overrightarrow{S_2}$$

where μ_B is the Bohr magneton, $\vec{B}$ is the applied external magnetic field, and $\vec{S}_1$ ($\vec{S}_2$) is the (effective) spin vector on the first (second) spin center, $\overleftrightarrow{\boldsymbol{g_1}}$ ($\overleftrightarrow{\boldsymbol{g_2}}$) is the electron *g*-tensor on the first (second) spin center, D_1 (D_2) is the axial zero-field splitting (ZFS) parameter on the first (second) spin center, E_1 (E_2) is the rhombic ZFS parameter on the first (second) spin center (the rhombicity parameter is defined such that $0 \leq E/D \leq 0.333$), $S_1 = S_2 = 1$, and J_{12} is the Heisenberg-Dirac-van Vleck (HDvV) exchange coupling constant between spin centers 1 and 2 (using the $-2J$ convention). The data were constrained such that the parameters for spin center 2 were the same as those for spin center 1, justified by the inversion center relating spin centers 1 and 2 by symmetry in the molecule. An axial $\overleftrightarrow{\boldsymbol{g_1}}$ ($\overleftrightarrow{\boldsymbol{g_2}}$)-tensor ($g_z = g_\parallel$; $g_x = g_y = g_\perp$) was used. Temperature-independent paramagnetism (TIP) was used in the spin-Hamiltonian model for the

magnetometry data in order to correct the values at high temperatures such that $\chi_P T$ leveled off at a constant value.

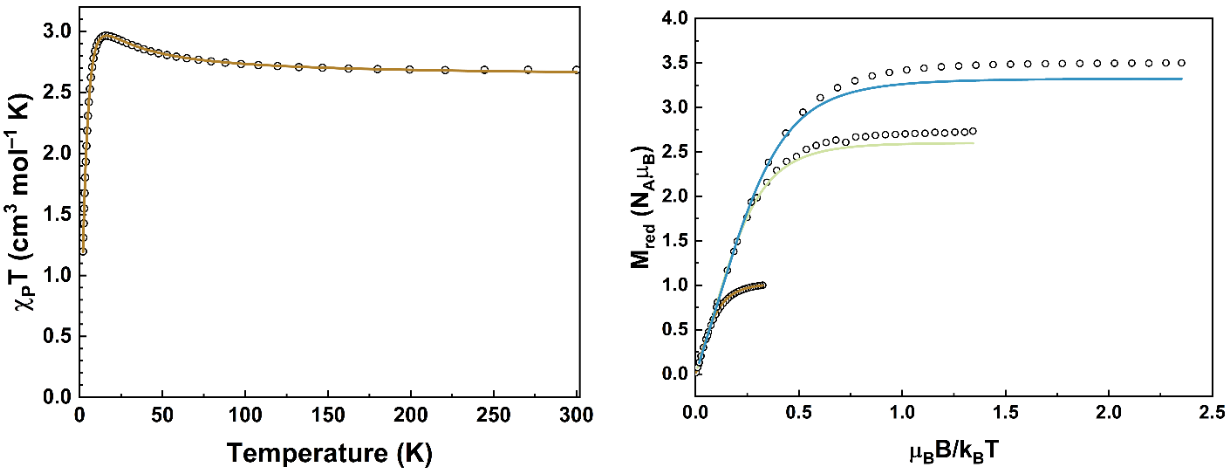

Figure S17: Left: Paramagnetic susceptibility (open circles) and spin Hamiltonian fit (brown trace) and right: VTVH reduced magnetization data (open circles) and spin Hamiltonian fit at 1, 4, and 7 T (brown, green, and blue traces).

The susceptibility and magnetization data shown in Figure S17 clearly show that complex **5** is a dimer, evidenced by the ferromagnetic coupling behavior. The following spin Hamiltonian parameters were used in the model to the data: $S_1 = S_2 = 1$, $D_1 = D_2 =$ +12.5(1.0) cm^{-1}, $E_1/D_1 = E_2/D_2 =$ 0.32(10), $g_{\perp 1} = g_{\perp 2} =$ 2.285(5), $g_{\parallel 1} = g_{\parallel 2} =$ 2.313(5), $J_{12} =$ +2.2(2) cm^{-1}, and TIP = 0.00054(3) cm^3 mol^{-1}. Although the rhombicity of the spin centers fit to a value near the rhombic limit ($E/D =$ 0.333), the large error accounts for relative insensitivity of the model to this parameter.

^{1}H NMR in DMF-d_7

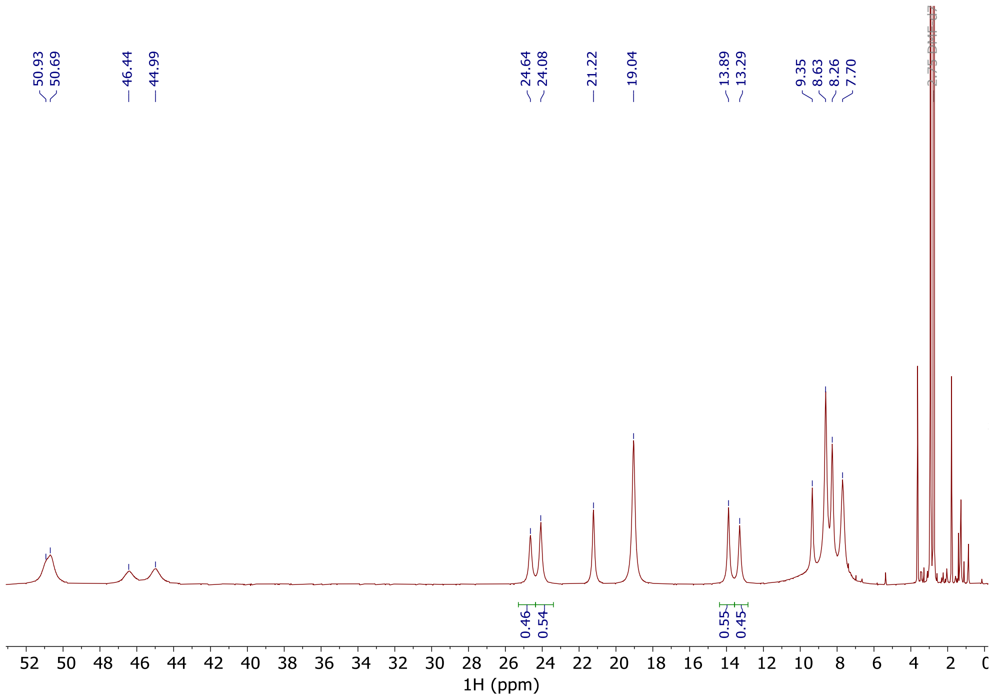

*Figure S18: ^{1}H NMR (400 MHz, 25 °C) of compound **5** in DMF-d$_7$. Due to overlapping, integration was only employed to determine the approximate ratio of the observed species (55 : 45).*

In the ^{1}H NMR spectrum of **5** in DMF-d_7 two paramagnetic species in an approximate ratio of 55 : 45 can be observed; free ligand is not observed.

From a solution of **5** in DMF, the following crystal structure (see crystallographic data section) was obtained *via* benzene vapor diffusion:

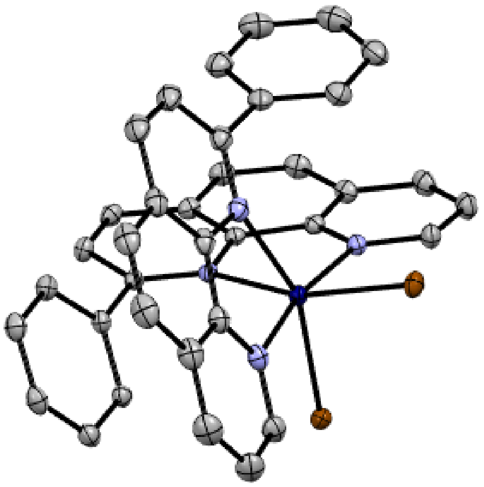

*Figure S19: Crystal structure obtained from a DMF solution of **5** via benzene vapor diffusion. Solvent molecules and hydrogen atoms omitted for clarity.*

This suggests that ligand exchange processes occur in DMF solution and that this might be one of the species observed in the ^{1}H NMR spectrum of **5** in DMF-d_7. This is in line with the previously proposed speciation of similar bipyridine-derived Ni-dihalide complexes.[3-5]

UV-Vis-NIR

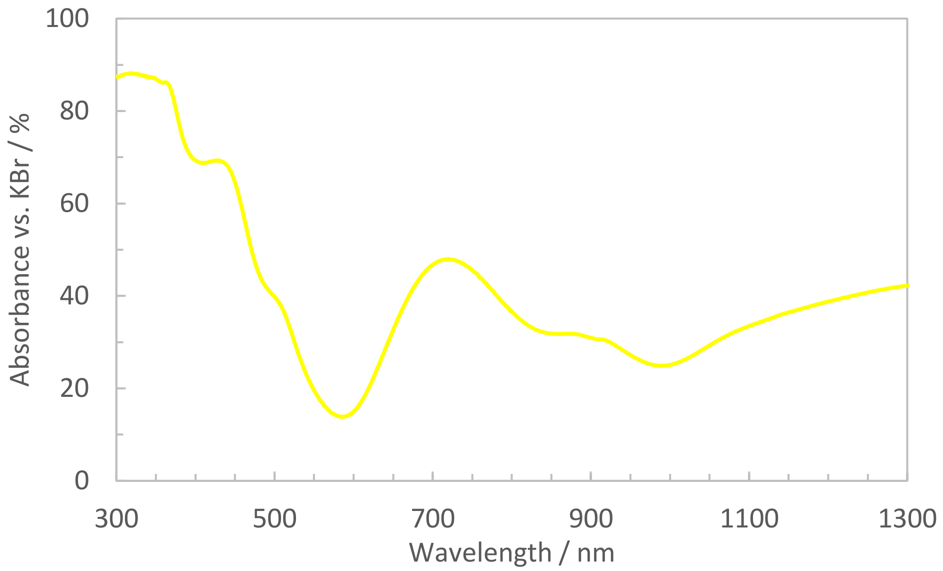

*Figure S20: UV-Vis-NIR spectrum of **5** in the solid state vs. KBr.*

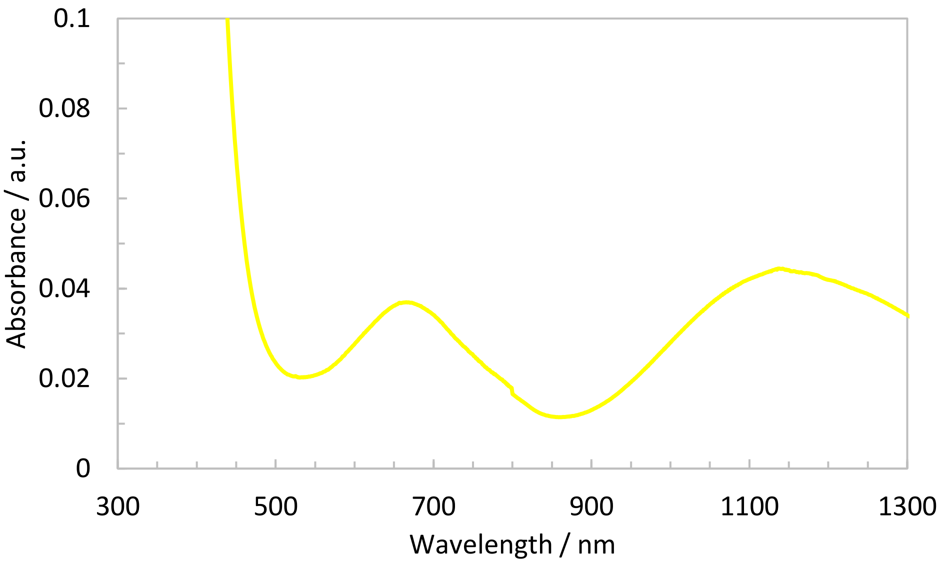

*Figure S21: UV-Vis-NIR of **5** in DMF solution (30 mM).*

Due to its low solubility, a UV-Vis-NIR of **5** in THF solution could not be recorded.

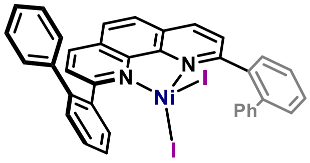

Complex 6

Following the **GPB**, NiI_2 (97 mg, 0.31 mmol, 1 equiv.), 2,9-di([1,1'-biphenyl]-2-yl)-1,10-phenanthroline (150 mg, 0.310 mmol, 1 equiv.), and THF (100 mL) were employed. Complex **6** was obtained in 88% yield (217 mg) as a brown powder. Crystals suitable for SC-XRD analysis were obtained *via* pentane vapor diffusion into a MeCN solution of **6**, and revealed a monomeric structure (see crystallographic data section).

^{1}H NMR (500 MHz, $CDCl_3$, 25 °C): δ 70.27 (s, 1H), 32.15 (bs, 1H), 26.22 (s, 1H), 22.84 (s, 1H), 8.51 (s, 1H), 7.17 (s, 1H), 6.75 (s, 2H), 5.35 (bs, 3H). *One proton could not be detected, perhaps due to the paramagnetism of the sample.*

^{1}H NMR (400 MHz, THF-d_8, 25 °C): δ 69.80 (s, 1H), 29.23 (bs, 1H), 25.84 (s, 1H), 22.79 (s, 1H), 8.18 (s, 1H), 7.15 (s, 1H), 6.80 (s, 2H), 5.61 (bs, 3H). *One proton could not be detected, perhaps due to the paramagnetism of the sample.*

UV-Vis-NIR

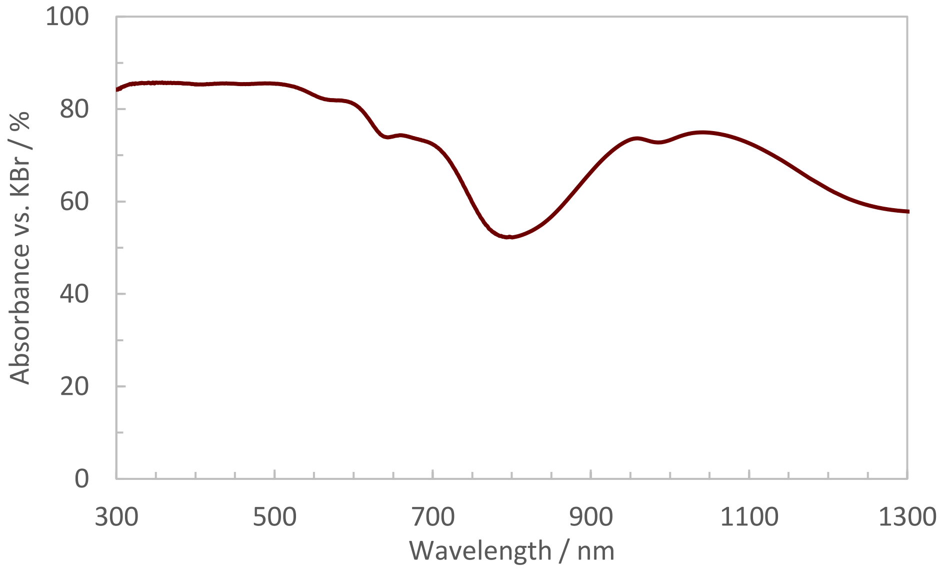

*Figure S22: UV-Vis-NIR spectrum of **6** in the solid state vs. KBr.*

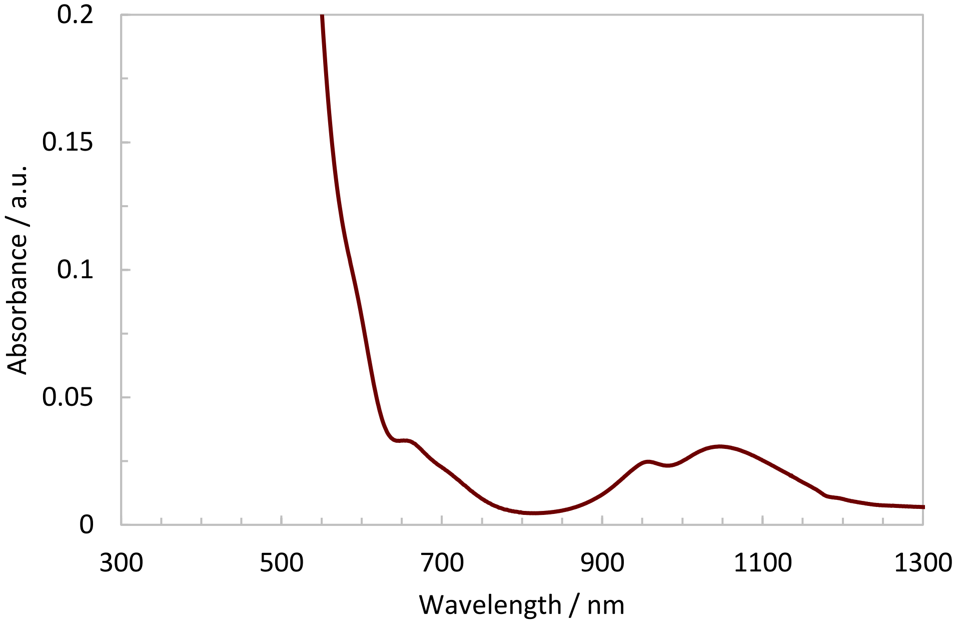

*Figure S23: UV-Vis-NIR of **6** in THF solution.*

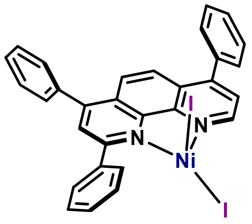

Complex 7

Following the **GPB**, NiI_2 (383 mg, 1.22 mmol, 1 equiv.), 2,4,7-triphenyl-1,10-phenanthroline (500 mg, 1.22 mmol, 1 equiv.), and THF (200 mL) were employed. Complex **7** was obtained in 96% yield (846 mg) as a brown powder. Crystals suitable for SC-XRD analysis were obtained *via* pentane vapor diffusion into a benzene solution of **7**, and revealed a monomeric structure (see crystallographic data section).

^{1}H NMR (400 MHz, $CDCl_3$, 25 °C): δ 73.53 (s, 1H), 68.04 (s, 1H), 33.31 (s, 2H), 26.21 (s, 1H), 10.84 (s, 1H), 9.15 (d, *J* = 7.7 Hz, 2H), 8.77 (d, *J* = 7.2 Hz, 2H), 8.67 (t, *J* = 7.7 Hz, 1H), 8.36 (t, *J* = 7.4 Hz, 1H), 7.24 (d, *J* = 5.5 Hz, 12H), 7.05 (t, *J* = 7.2 Hz, 2H), 3.63 (s, 2H).

^{1}H NMR (400 MHz, THF-d_8, 25 °C): δ 72.74 (s, 1H), 66.54 (s, 1H), 33.28 (s, 1H), 32.18 (s, 1H), 25.86 (s, 1H), 10.88 (s, 1H), 9.35 (s, 2H), 8.85 (s, 2H), 8.72 (s, 1H), 8.36 (s, 1H), 7.15 (s, 2H), 7.01 (s, 2H), 4.04 (s, 2H), 3.28 (s, 2H).

UV-Vis-NIR

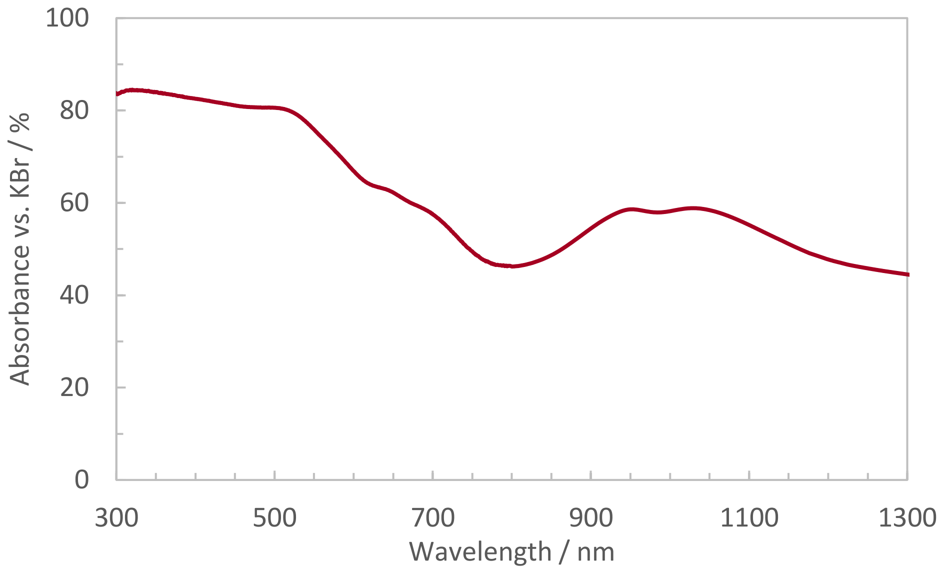

*Figure S24: UV-Vis-NIR spectrum of **7** in the solid state vs. KBr.*

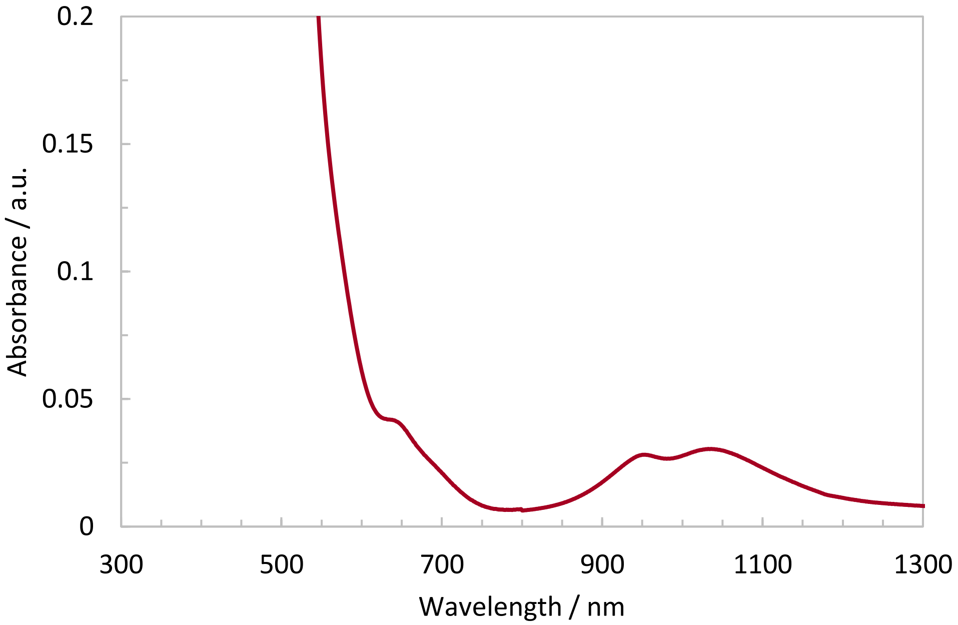

*Figure S25: UV-Vis-NIR of **7** in THF solution.*

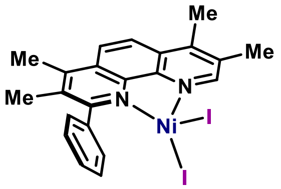

Complex 8

Following the **GPB**, NiI_2 (50 mg, 0.16 mmol, 1 equiv.), 3,4,7,8-tetramethyl-2-phenyl-1,10-phenanthroline (50 mg, 0.16 mmol, 1 equiv.), and THF (100 mL) were employed. Complex **8** was obtained in 98% yield (98 mg) as a brown powder. Crystals suitable for SC-XRD analysis were obtained *via* pentane vapor diffusion into a $CDCl_3$ solution of **8**. Crystals suitable for SC-XRD analysis were obtained *via* pentane vapor diffusion into a $CDCl_3$ solution of **8**, and revealed a monomeric structure (see crystallographic data section).

*NMR spectra of complexes featuring this ligand (**3** and **8**) always miss two hydrogen atoms. In the crystal structures of these complexes, no abnormalities can be observed in the ligand (vide infra). Therefore, missing signals can be attributed to the paramagnetic nature of the complexes.*

^{1}H NMR (400 MHz, $CDCl_3$, 25 °C): δ 33.17 (s, 1H), 26.96 (s, 1H), 17.94 (s, 1H), 13.73 (s, 3H), 12.76 (s, 3H), 7.64 (s, 1H), 4.35 (s, 2H), -9.27 (s, 3H), -12.07 (s, 3H).

^{1}H NMR (400 MHz, THF-*d*$_8$, 25 °C): δ 33.09 (s, 1H), 26.91 (s, 1H), 17.09 (s, 1H), 13.34 (s, 3H), 12.76 (s, 3H), 7.49 (s, 1H), 4.66 (s, 2H), -10.13 (s, 3H), -12.69 (s, 3H).

UV-Vis-NIR

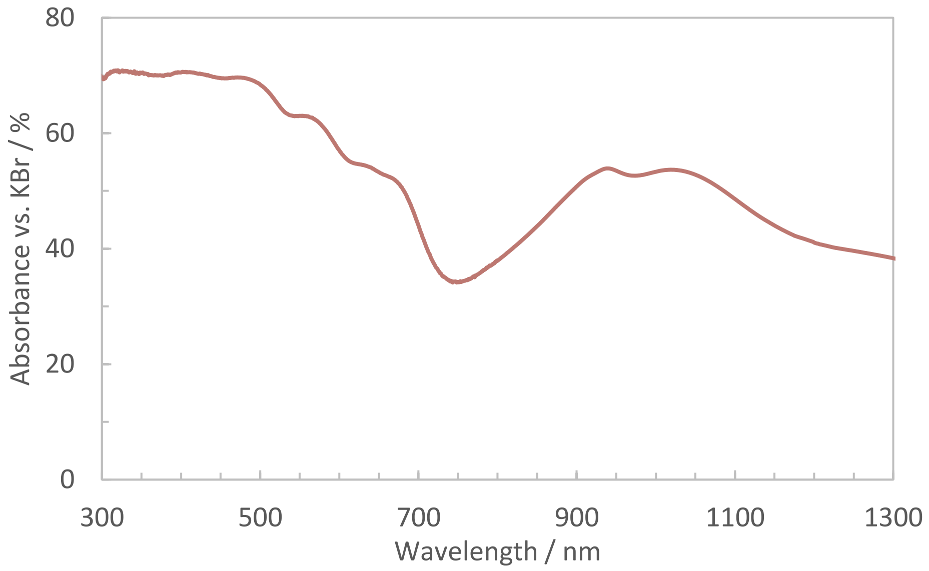

*Figure S26: UV-Vis-NIR spectrum of **8** in the solid state vs. KBr.*

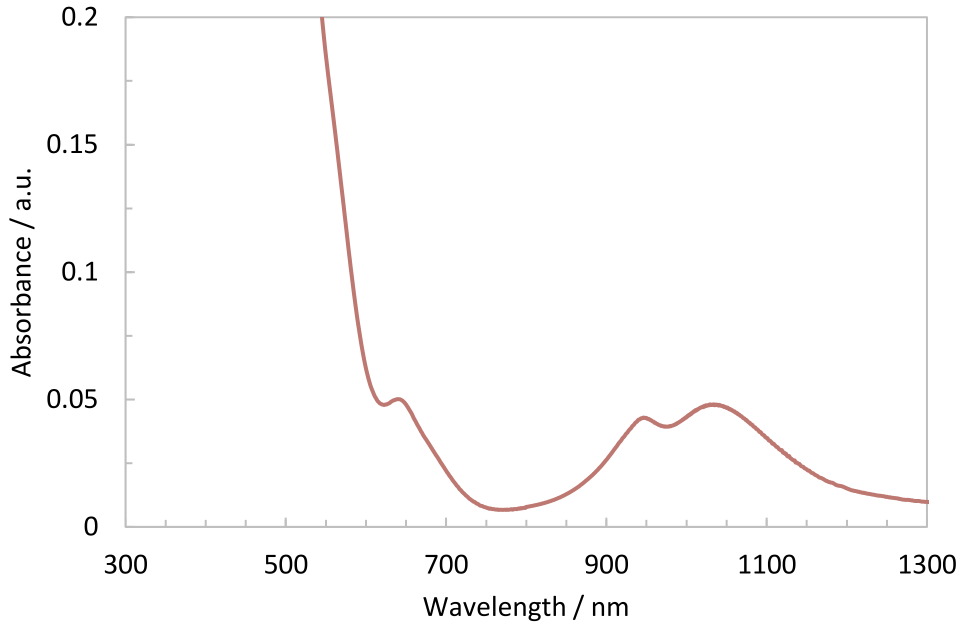

*Figure S27: UV-Vis-NIR of **8** in THF solution.*

3. Collected UV-Vis-NIR spectra and simulations

UV-Vis-NIR in the solid state

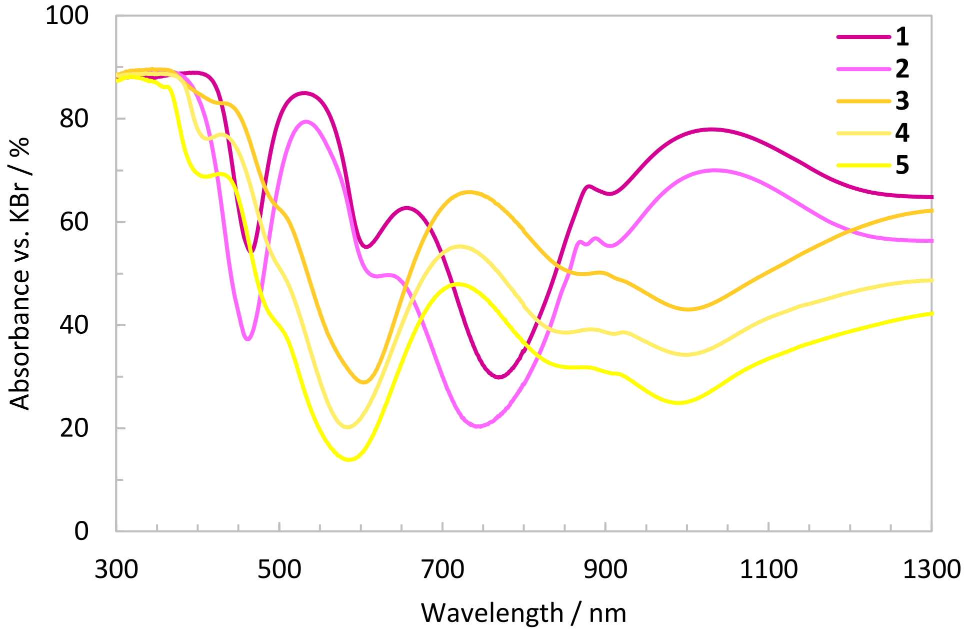

*Figure S28: Superimposed UV-Vis-NIR spectra of LNiBr$_2$ complexes **1**-**2** and [LNiBr$_2$]$_2$ complexes **3**-**5** in the solid state.*

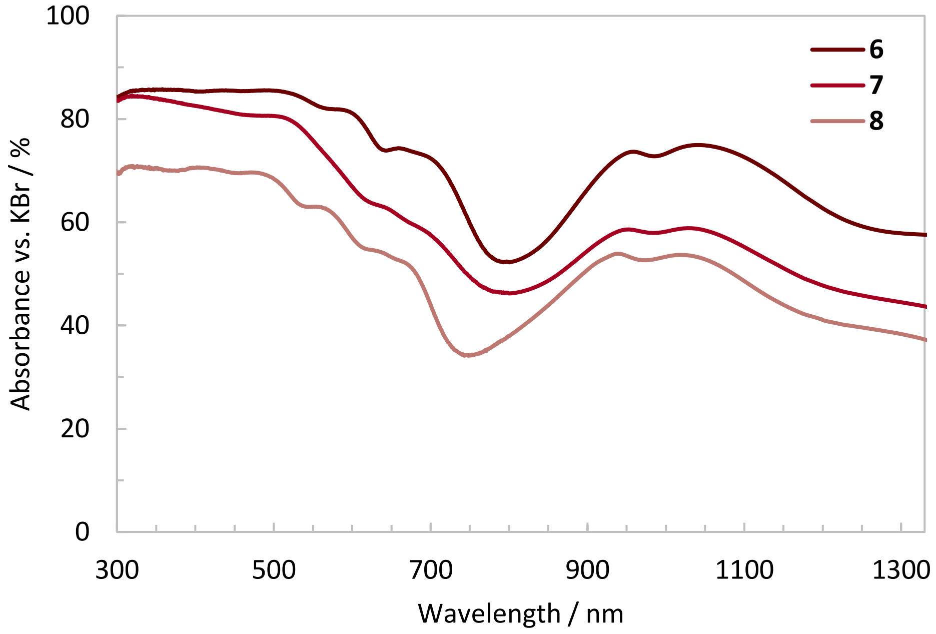

*Figure S29: Superimposed UV-Vis-NIR spectra of LNiI$_2$ complexes **6**-**8** in the solid state.*

UV-Vis-NIR in DMF

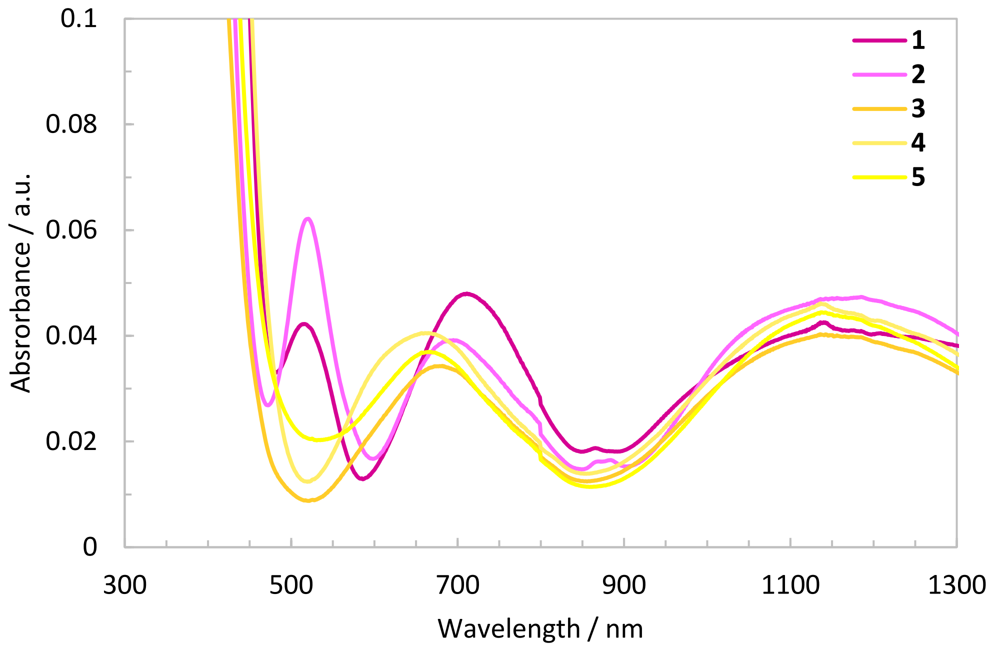

*Figure S30: Superimposed UV-Vis-NIR spectra of $LNiBr_2$ complexes **1**-**5** in DMF solution (30 mM).*

UV-Vis-NIR in THF

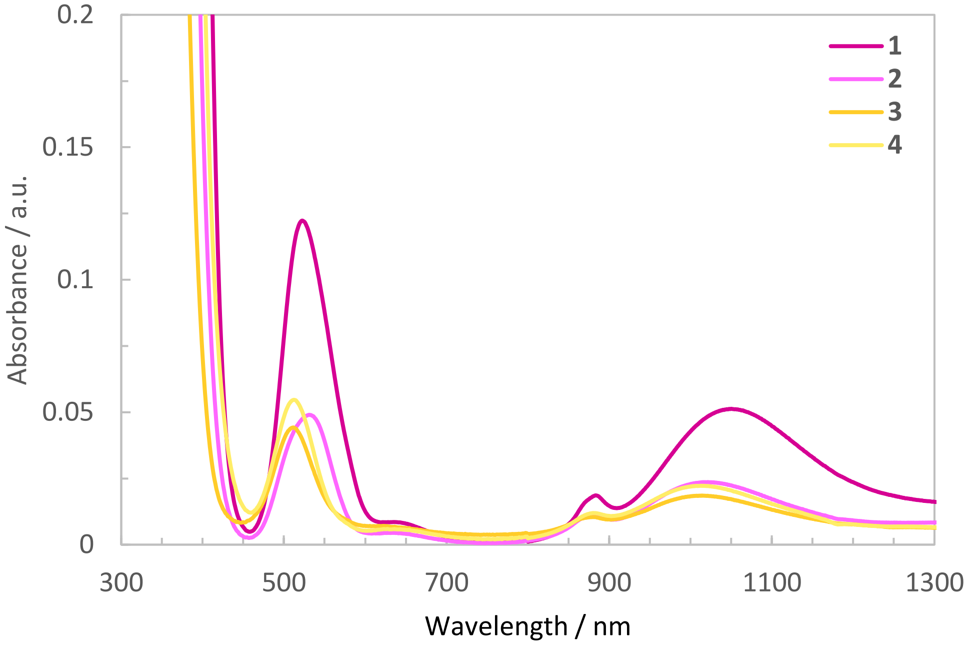

*Figure S31: Superimposed UV-Vis-NIR spectra of $LNiBr_2$ complexes **1-4** in THF solution.*

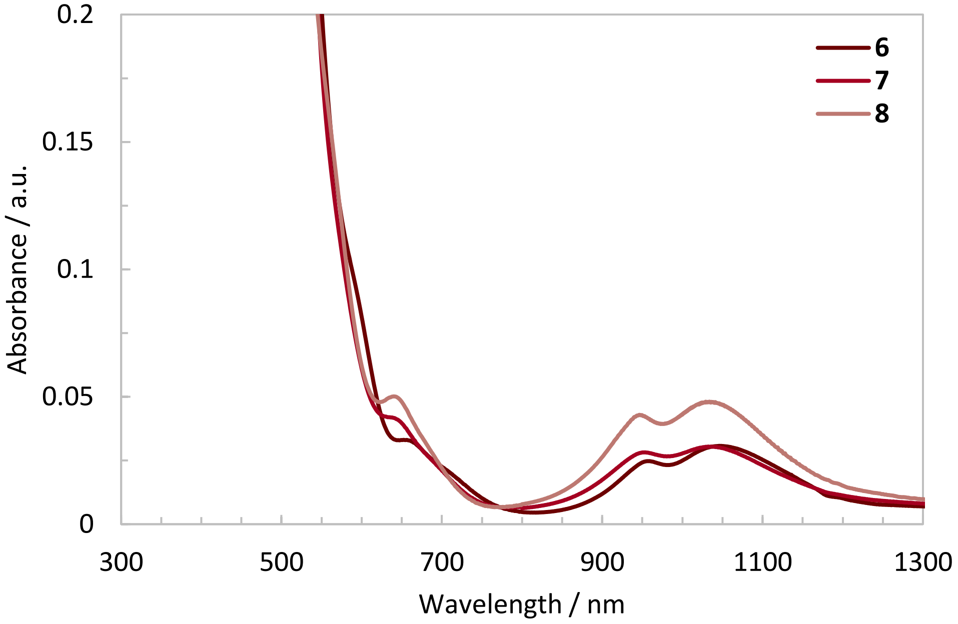

*Figure S32: Superimposed UV-Vis-NIR spectra of $LNiI_2$ complexes **6-8** in THF solution.*

Calculated Spectra and Selected Transitions

All calculations have been performed with the ORCA quantum chemistry package.[6] Full Geometry optimizations have been performed with the BP86 functional[7] using x2c basis set[8, 9] and x2c/j auxiliary basis set.[8, 9] Furthermore, the cpcm solvent model[10] was used to model the THF solvent along with the D3BJ dispersion correction.[11] In case of the dimeric systems, the broken symmetry formalism[12, 13] with two α and two β electrons was used to account for local triplet Ni^{2+} ions that anti-ferromagnetically couple. TDDFT calculations wit 300 roots have subsequently been performed on the optimized geometries using the B3LYP functional.[14, 15]

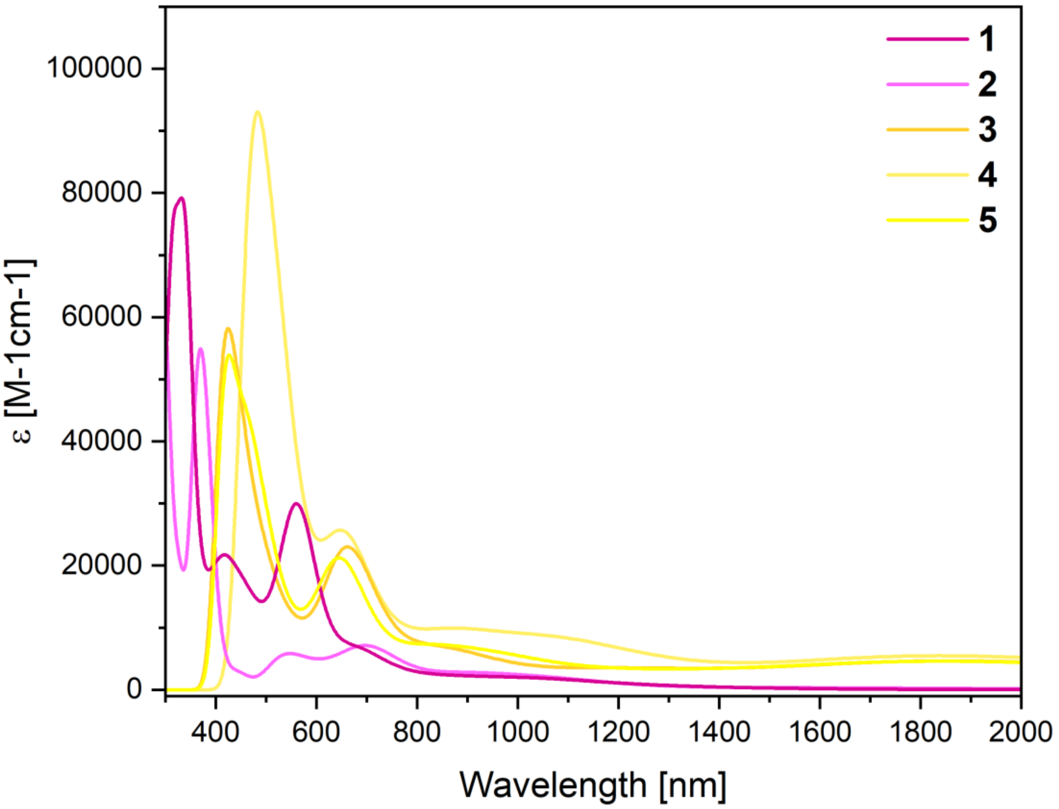

*Figure S33: Calculated UV-Vis-NIR spectra of complexes **1**-**5**.*

Difference densities depicted in yellow (positive) and red (negative)

Complex 1

415.0nm

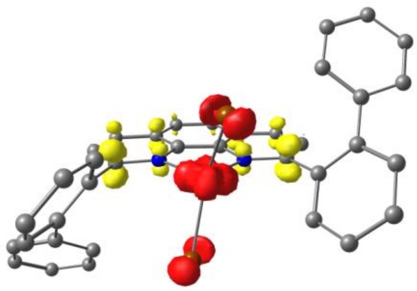

569.2nm

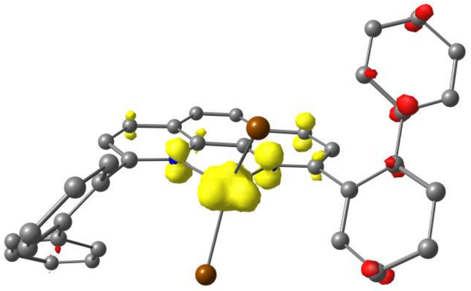

678.0nm

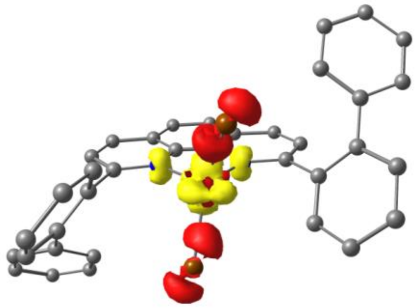

901.3nm

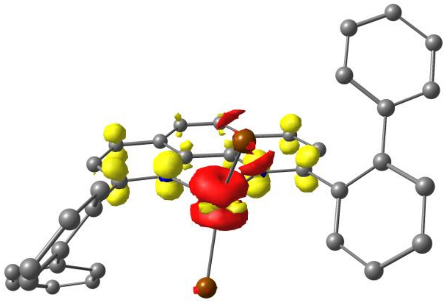

1240.8nm

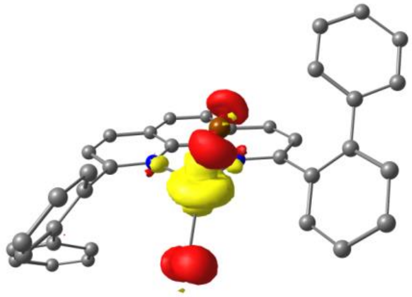

Complex 2

368.8nm

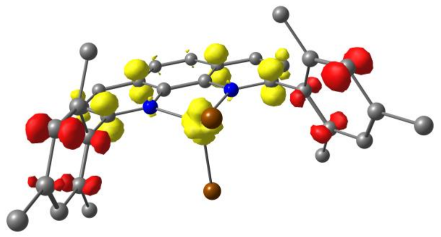

544.8nm

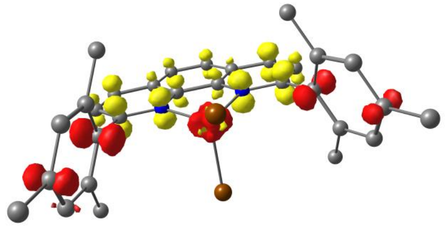

692.1nm

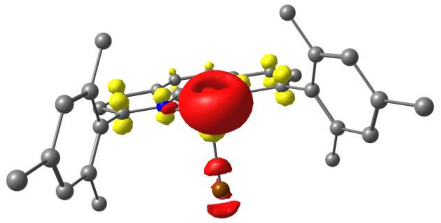

1086.4nm

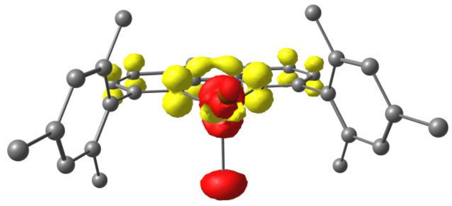

1530.0nm

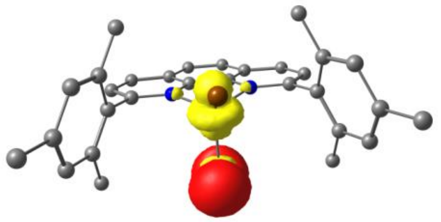

Complex 3

423.6nm

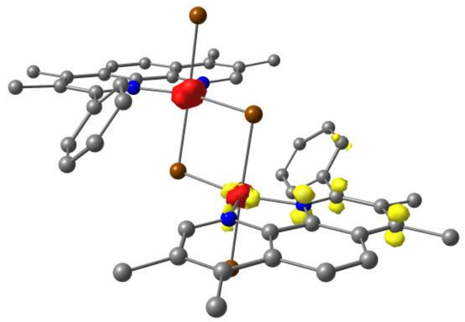

423.8nm

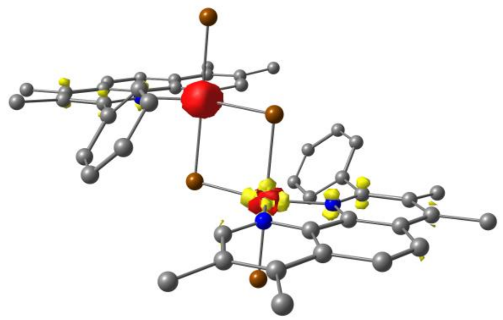

665.3nm

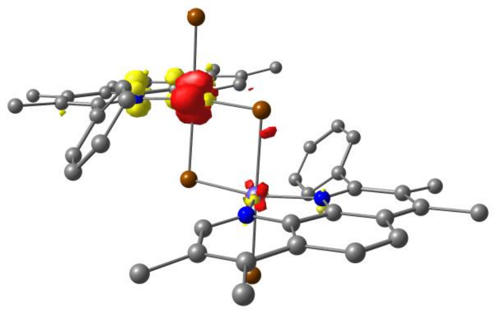

884.9nm

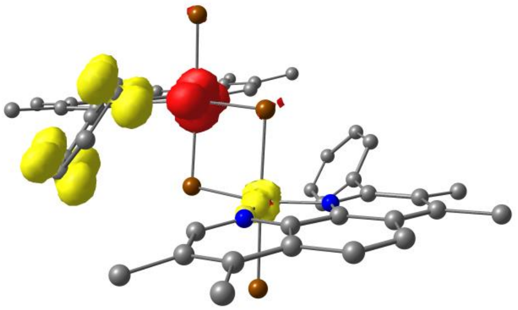

1197.5nm

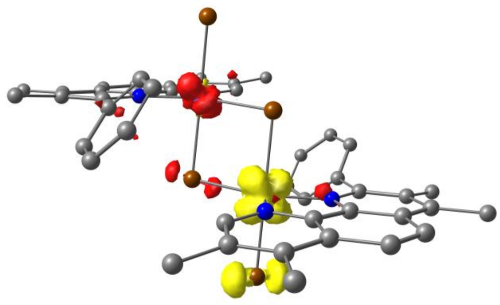

1844.3nm

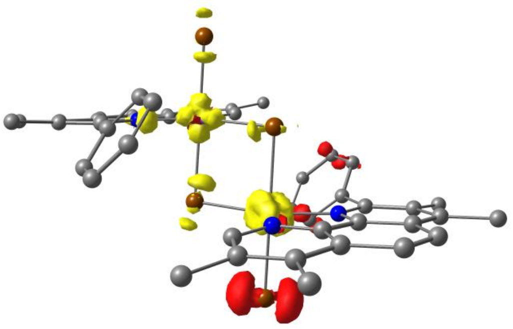

Complex 4

481.1 nm

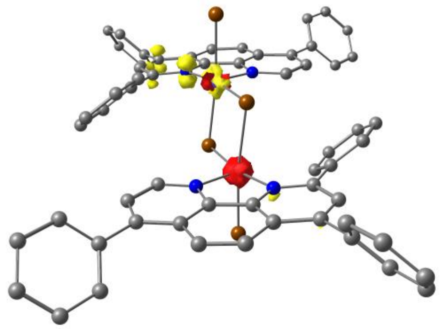

694.1 nm

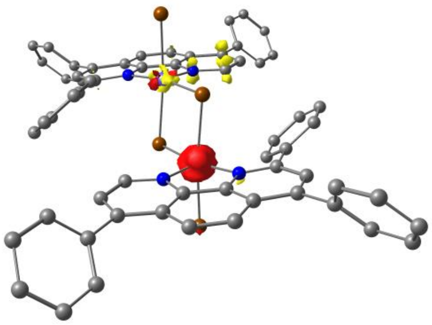

948.1 nm

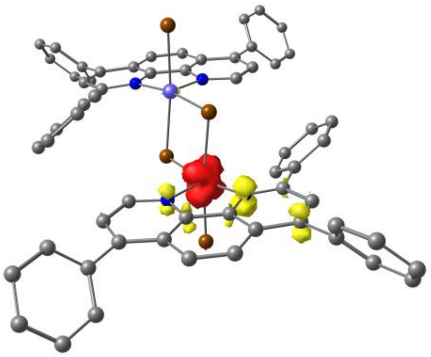

1221.6 nm

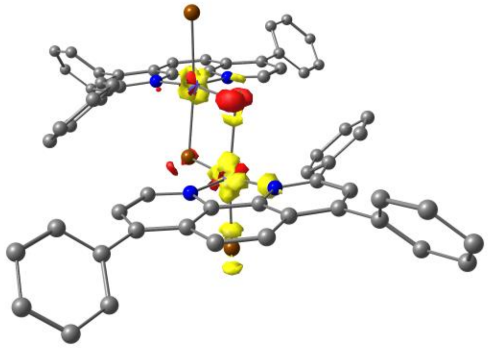

Complex 5

464.0 nm

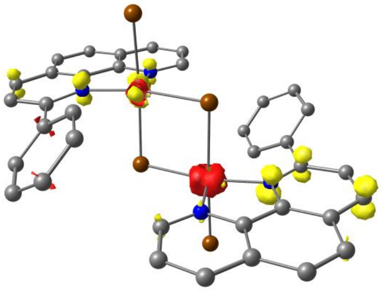

611.1 nm

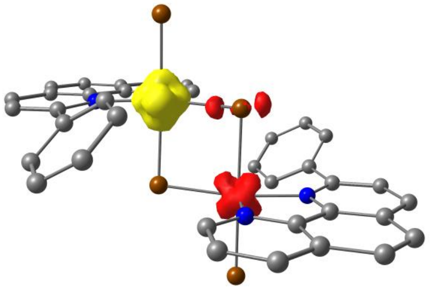

645.3 nm

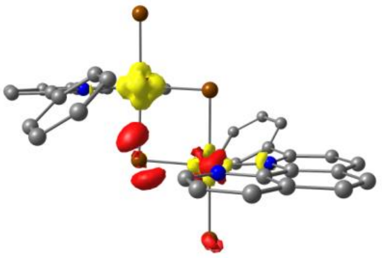

833.5 nm

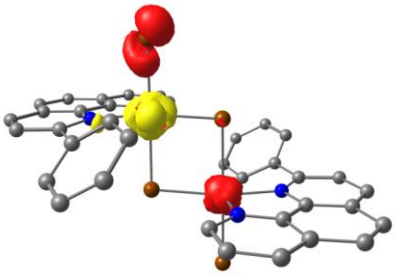

981.7 nm

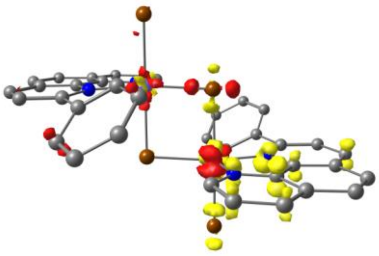

1303.0 nm

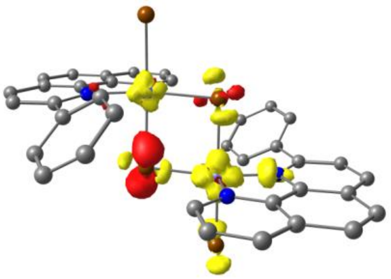

1419.1 nm

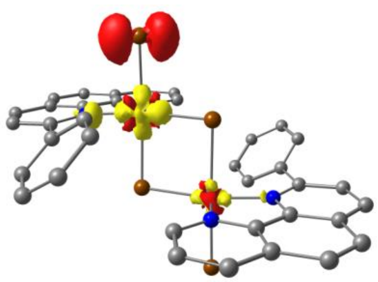

1894.1 nm

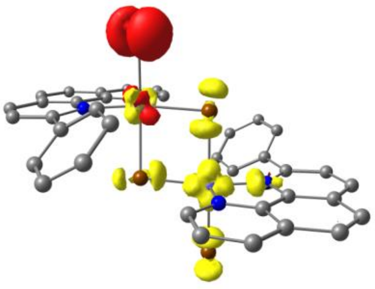

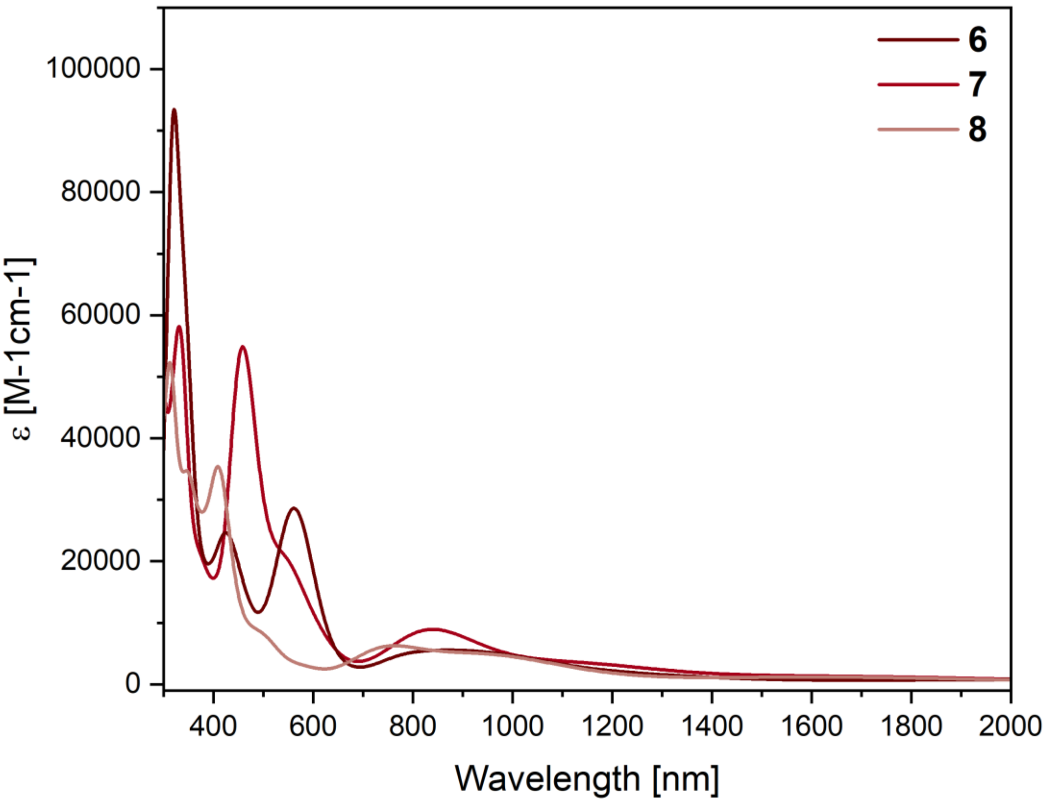

*Figure S34: Calculated UV-Vis-NIR spectra of complexes **6**-**8**.*

Difference densities depicted in yellow (positive) and red (negative)

Complex 6

321.0 nm

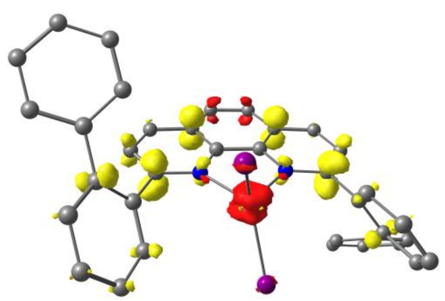

434.4 nm

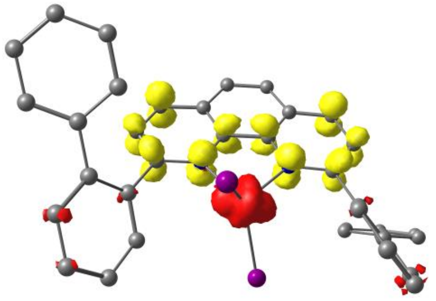

569.6 nm

898.6 nm

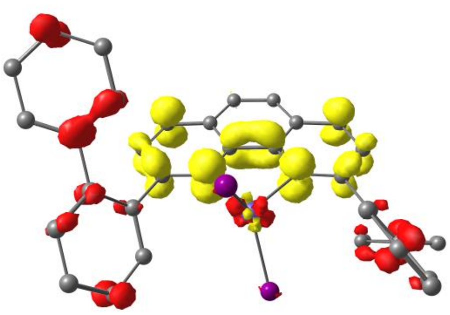

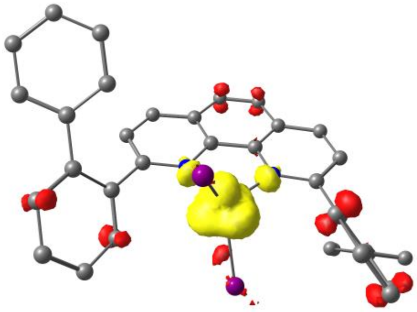

Complex 7

325.6 nm

491.9 nm

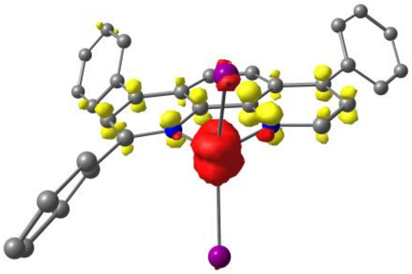

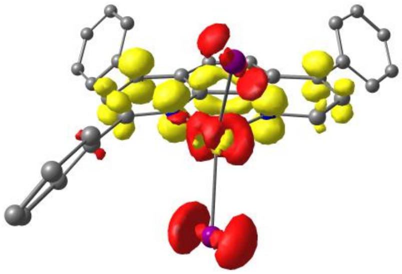

556.1 nm

806.6 nm

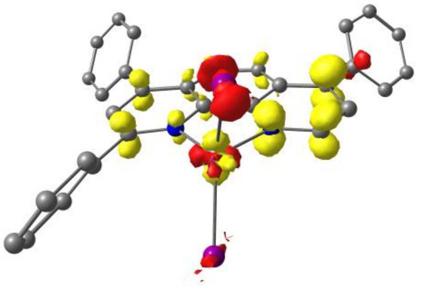

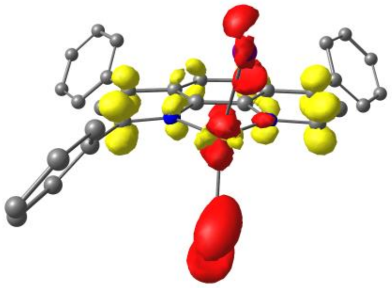

Complex 8

309.1 nm

410.3 nm

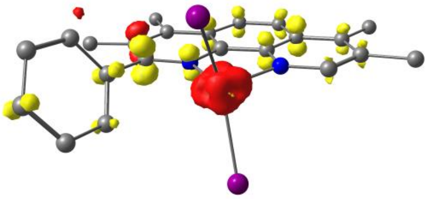

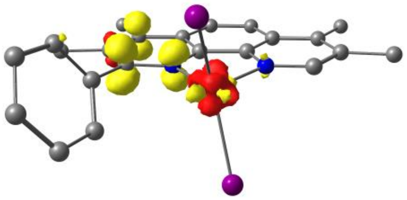

411.9 nm

443.8 nm

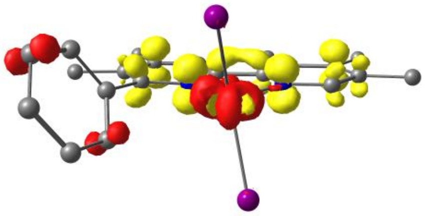

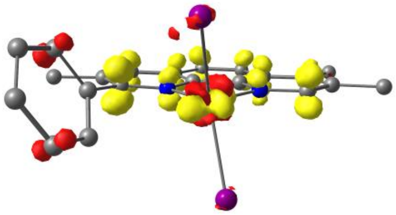

757.0 nm

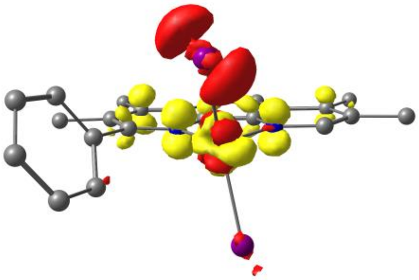

889.9 nm

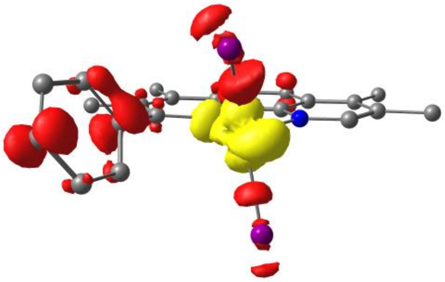

4. DOSY NMR of complexes 1-4

Diffusion coefficients were obtained from a double stimulated echo sequence with bipolar gradient pulsed, convection compensation, longitudinal eddy current delay, and three spoiler gradients (Bruker sequence: dstebpgp3s). The data was acquired at a Bruker AVIII 500 MHz NMR spectrometer equipped with a PA BBO 500S1 BBF-H-D Z PLUS (G_{max} = 53.5 G·cm^{-1}) at 298 K. The gradient pulse strength *G* was incremented from 5% to 98% of the maximum gradient strength (G_{max}) with a linear gradient ramp in 40 steps. The diffusion time (delay d20) used was 75 ms and the length of a gradient pulse (δ/2) of the encoding gradient was 1.0 ms. After the measurement, the dataset was processed with the AU program *setdiffparm* in Bruker Topspin 3.6.3 or 3.7.0 and then imported into in MNOVA 15.0.0 and processed therein. The imported 2D DOSY dataset was Fourier-transformed with 10 Hz line broadening and afterwards phased and baseline corrected. 2D DOSY plots were obtained using the DOSY transform implemented in MNOVA 15.0.0 following the options described in Table S1.

Table S1: MNOVA settings used for the 2D DOSY plots.

Method	Peak Fit
**Peak Fit Option**	
Decay components	Single
Fit Mode	Normal
Autocorrect Peak Positions	No
Confidence interval	95%
Scaling	
Method	No scaling
**DOSY Spectrum**	
Units	m^2/sec
Points in diff. dim.	160
Scale	manual
min	1.00E-10
max	3.00E-09

*Table S2: Overview of all determined self-diffusion constants D for complexes **1**-**4** and their ligands. For simplicity, in this section the ligands have been named **Lx** according to the (**Lx**)$NiBr_2$ complex **x** they form.*

**Complexes**							
	**D_{exp} (10^{-9} m^2/s)**						
**Name**	D_1	D_2	D_3	D_4	**average**		M (g mol^{-1})
**1**	0.869	0.862	0.874	0.834	**0.86**	**0.02**	703.1
**2**	0.913	0.949	0.911		**0.92**	**0.02**	635.07
**3**	1.02	1.01	1.06		**1.03**	**0.03**	530.92
**4**	0.864	0.882	0.877	0.865	**0.87**	**0.01**	627.01
**Ligands**							
**L1**	0.876	0.886	0.896	0.885	**0.89**	**0.01**	484.6
**L2**	0.940	0.968	0.934	0.956	**0.95**	**0.02**	416.57
**L3**	1.05	1.02	1.04	1.04	**1.04**	**0.01**	312.42
**L4**	0.929	0.930	0.932	0.933	**0.93**	**0.00**	408.5

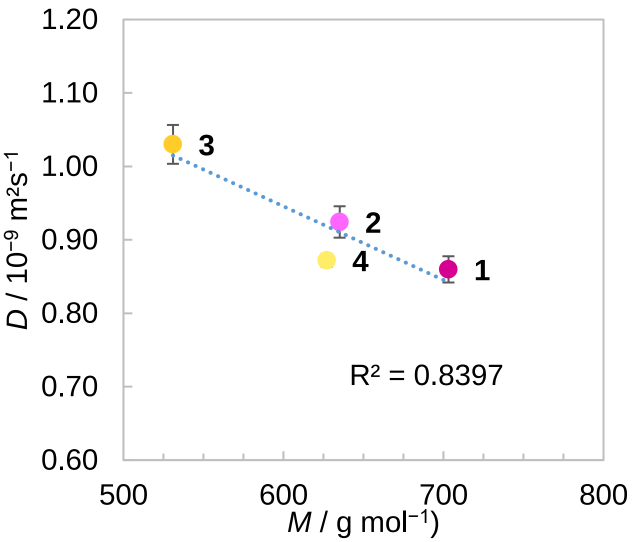

Figure S35: Correlation of the complexes with the molecular weights of their monomers.

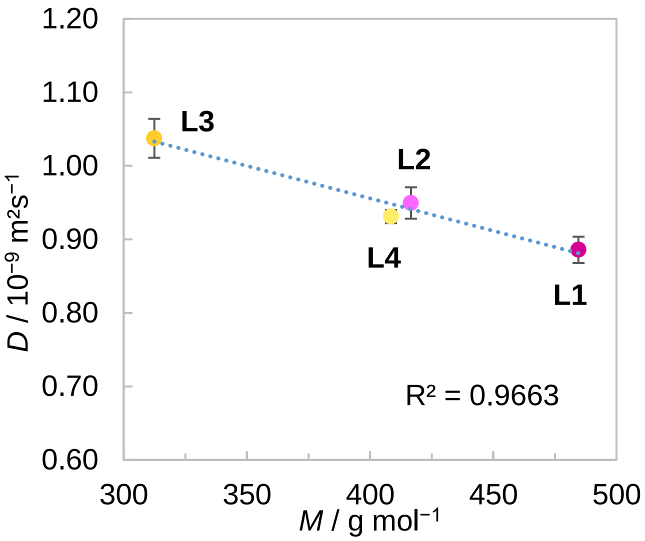

Figure S36: Correlation of the ligands to their molecular weights.

The results of the DOSY experiments are summarized in Table S2, as well as depicted in Figures S35 and S36. For both the complexes and ligands, the experimental self-diffusion coefficient decreases with rising molecular weights (calculated for the monomeric forms of the complexes). Assuming the radii of the complexes and ligands correlate linearly to their molecular weights, the trend observed is as expected from the Stokes-Einstein-Sutherland equation, in which the diffusion coefficient D is inversely proportional to the Stokes radius r of the solvate particles (T, absolute temperature; k_B, Boltzmann constant; η, dynamic viscosity):

$$\boldsymbol{D} = \frac{k_B T}{6\pi\eta \boldsymbol{r}}$$

The small deviation of complex **4** from the linear correlation is, to a lesser extent, also observed for its corresponding free ligand. This likely derives from the positioning of the phenyl groups at the backbone of the phenanthroline, which alters the shape of both the ligand and its complex compared to the other ones studied. Consequently, the assumed linear correlation between the molecular radius and the molecular weight might not strictly apply in this case.

Nevertheless, as depicted in Figure S37, complexes **1**-**4** all display similar diffusion coefficients as their free ligands. In case of dimerization, the radius of the complexes would roughly be double the one of the free ligands, thereby affording a diffusion coefficient half as high. As this is not observed, the experimental DOSY data supports a predominantly monomeric structure for complexes **1**-**4** in $CDCl_3$.

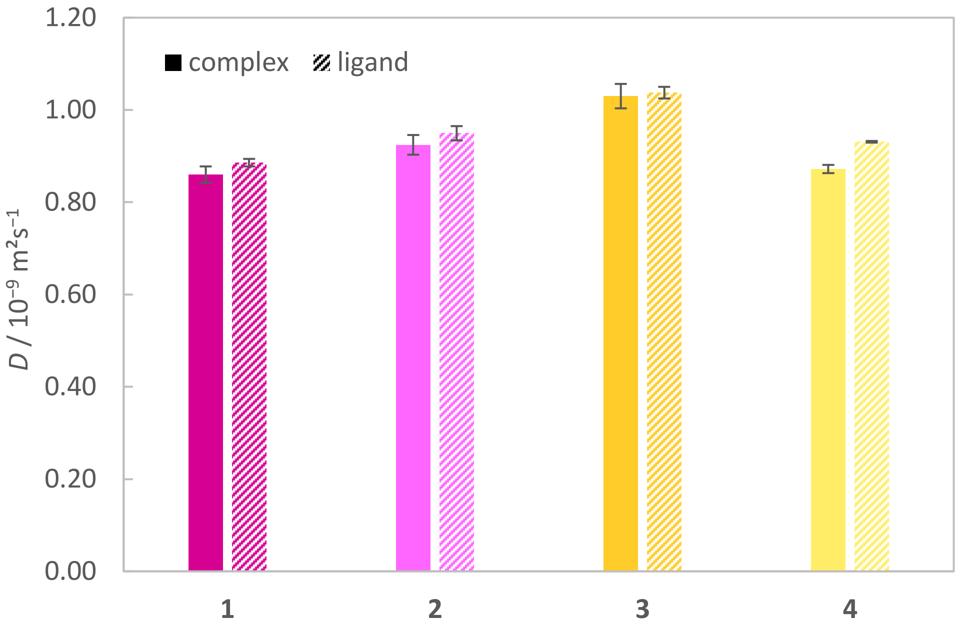

*Figure S37: Self-diffusion coefficients of complexes **1**-**4** and their ligands*

2D DOSY spectra of complexes 1-4

Comment: Due to the paramagnetic properties of the complexes, the ^{1}H nuclei exhibit significantly shorter relaxation times. Consequently, during the pulse sequence, they experience greater signal attenuation, resulting in DOSY signal intensities that are substantially lower than those of diamagnetic impurities.

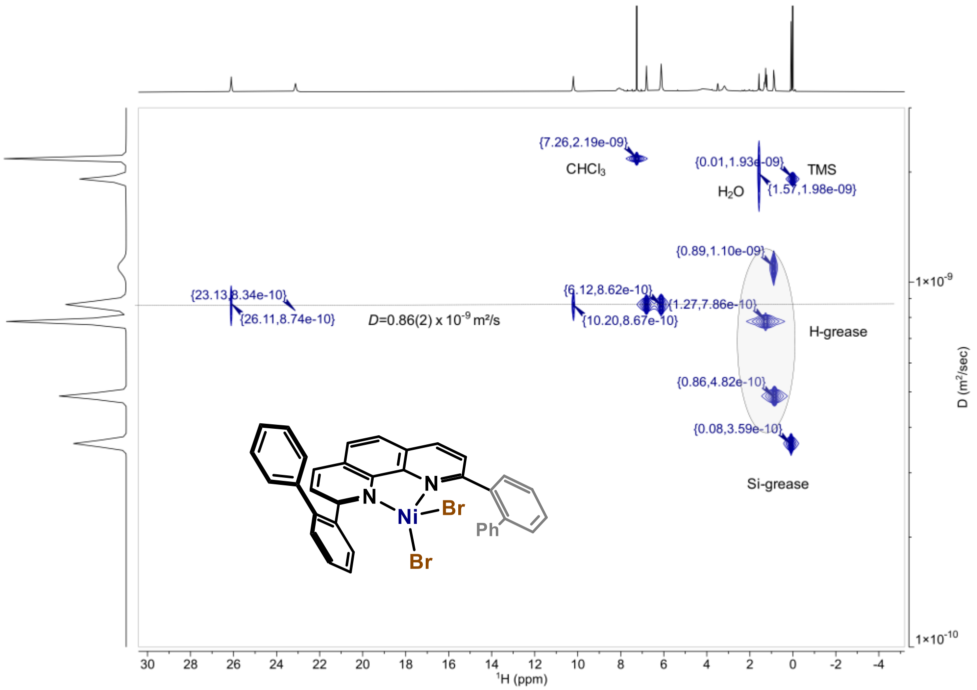

*Figure S38: 2D DOSY spectrum of complex **1** in $CDCl_3$, 500 MHz, 298 K.*

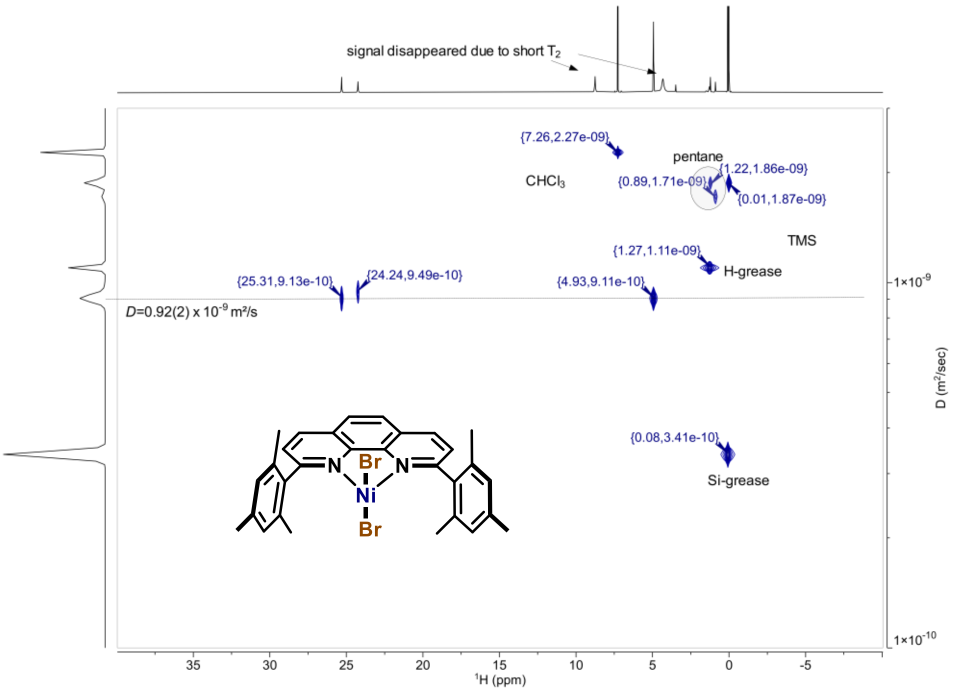

*Figure S39: 2D DOSY spectrum of complex **2** in $CDCl_3$, 500 MHz, 298 K.*

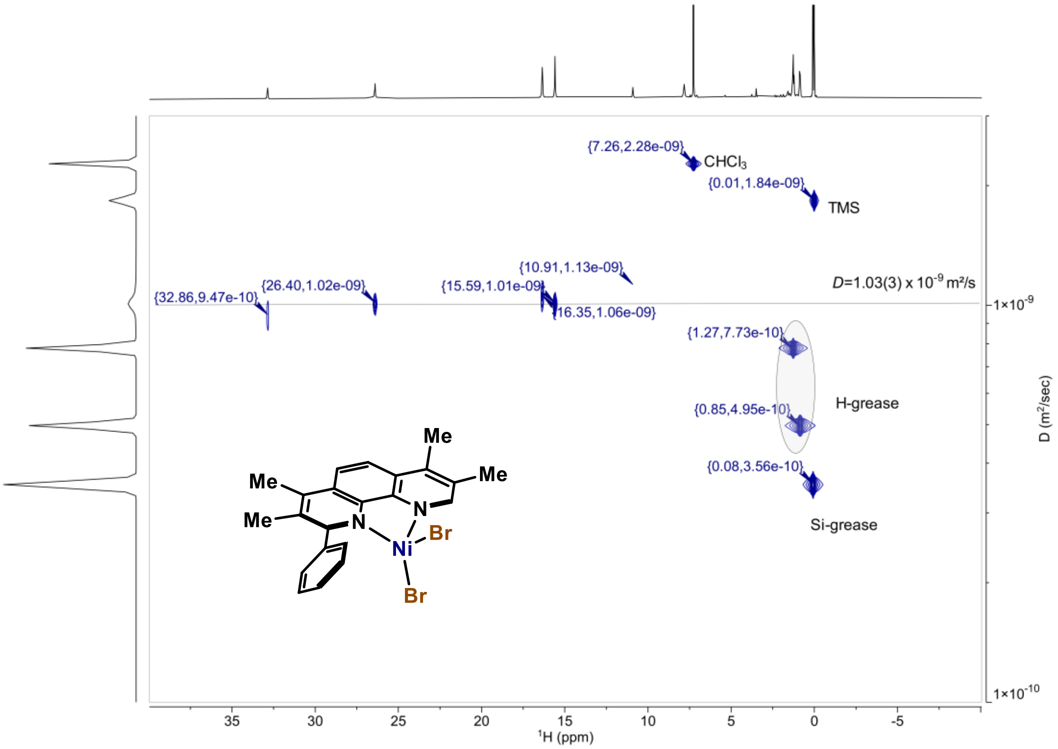

*Figure S40: 2D DOSY spectrum of complex **3** in $CDCl_3$, 500 MHz, 298 K.*

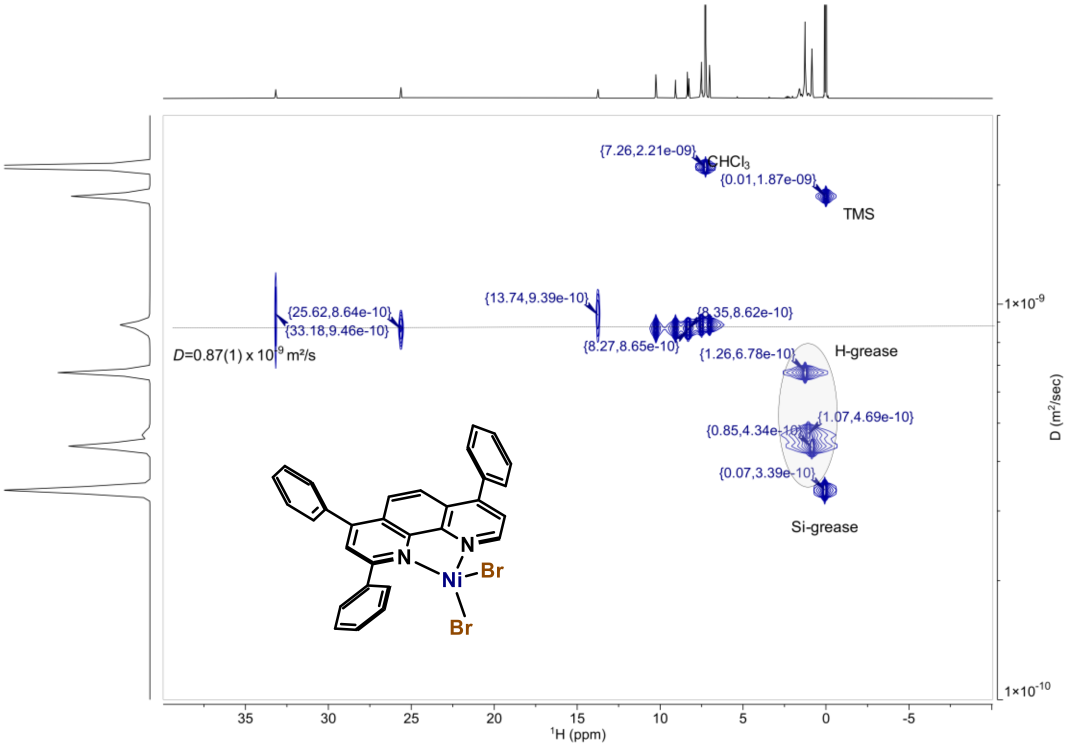

*Figure S41: 2D DOSY spectrum of complex **4** in $CDCl_3$, 500 MHz, 298 K.*

2D DOSY spectra of ligands L1-L4

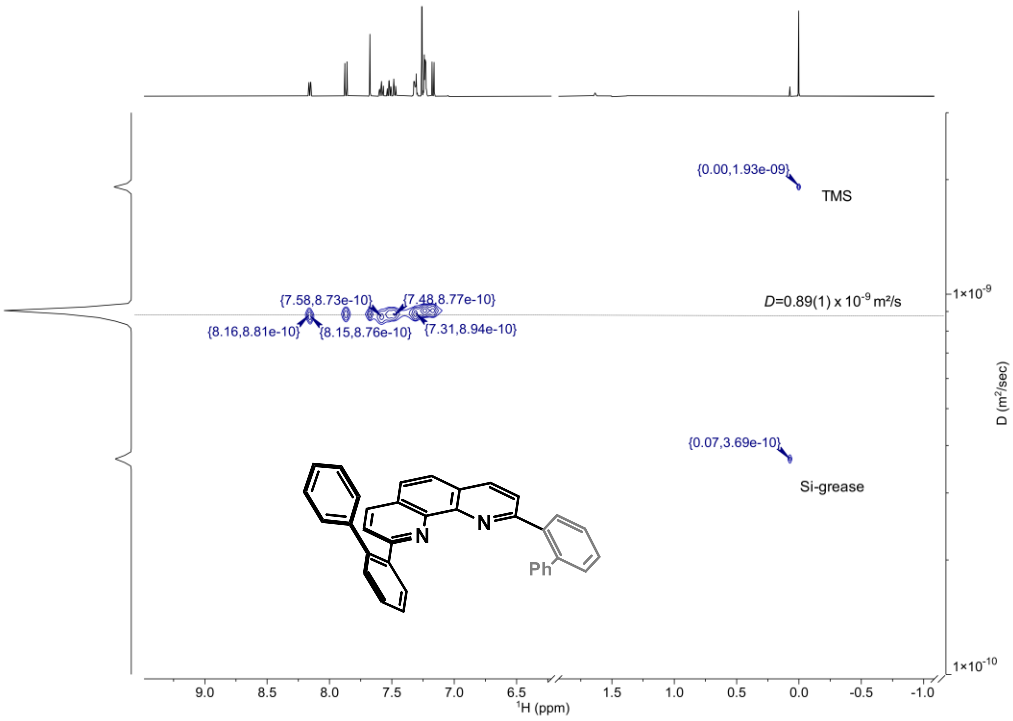

*Figure S42: 2D DOSY spectrum of Ligand **L1** in $CDCl_3$, 500 MHz, 298 K.*

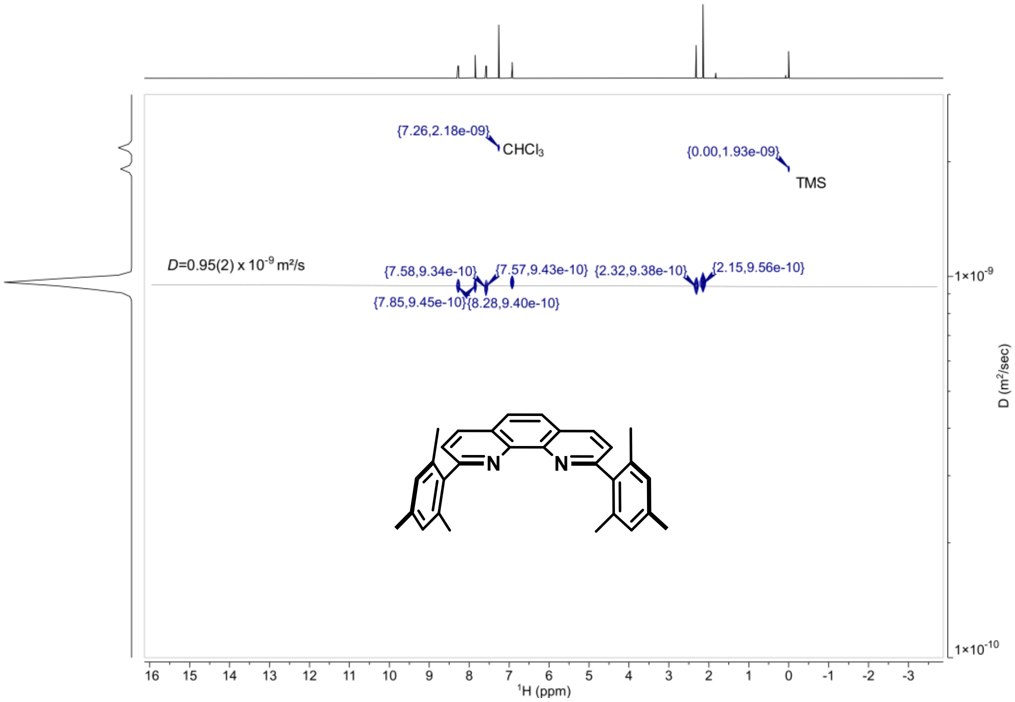

*Figure S43: 2D DOSY spectrum of Ligand **L2** in $CDCl_3$, 500 MHz, 298 K.*

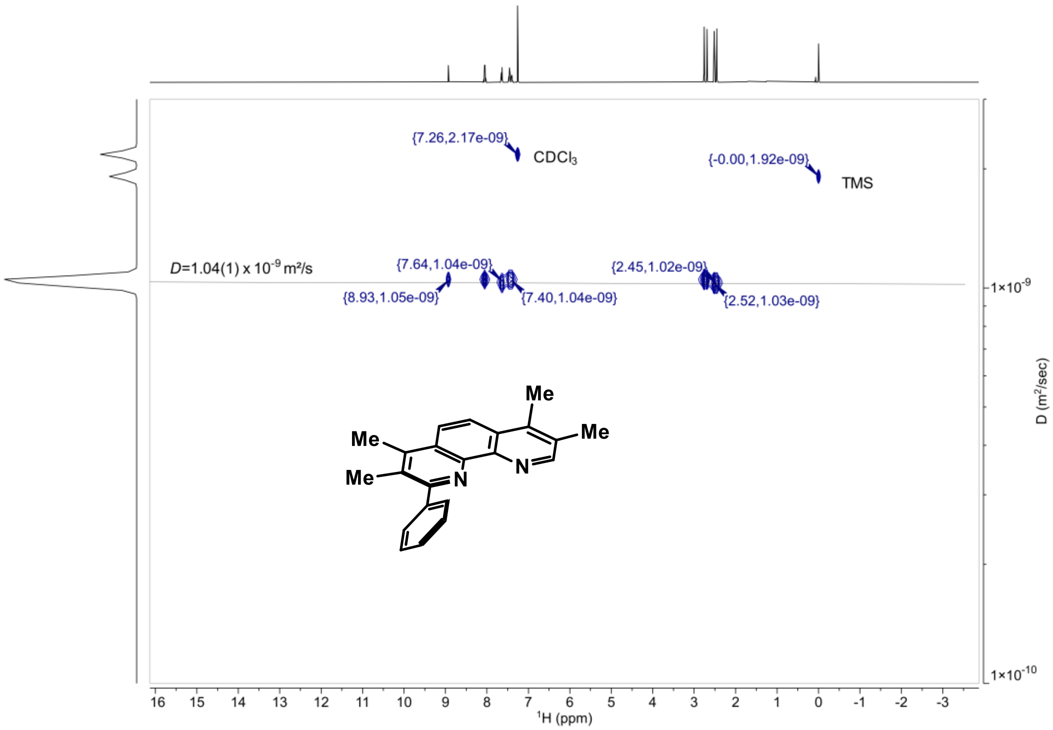

*Figure S44: 2D DOSY spectrum of Ligand **L3** in CDCl₃, 500 MHz, 298 K.*

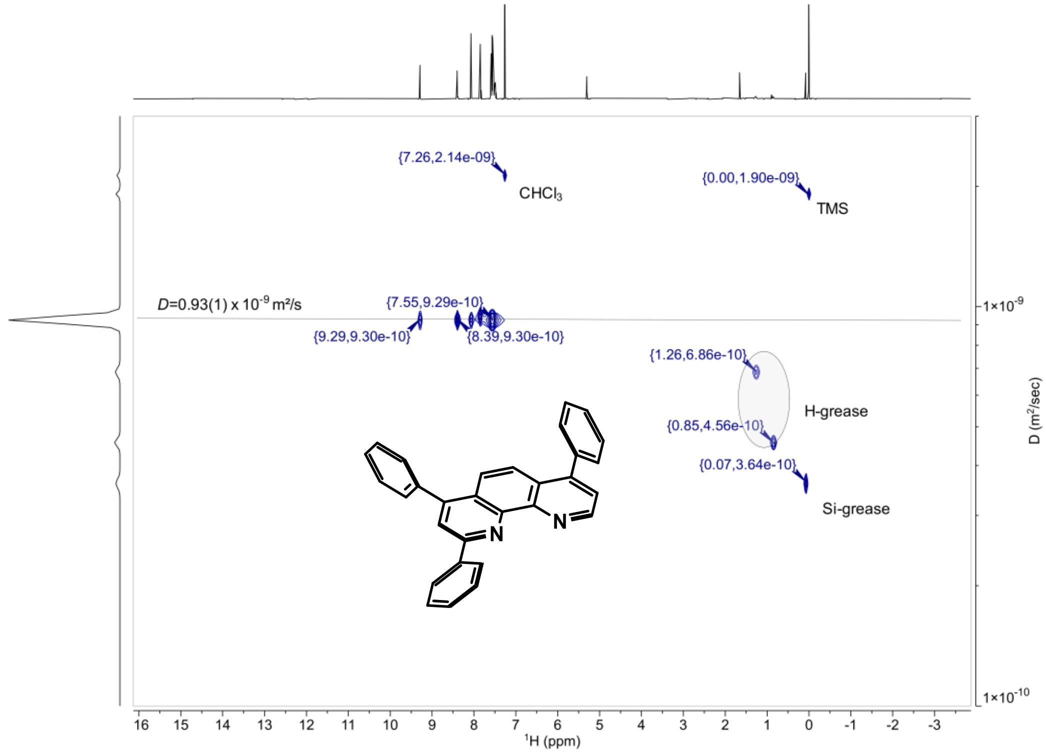

*Figure S45: 2D DOSY spectrum of Ligand **L4** in CDCl₃, 500 MHz, 298 K.*

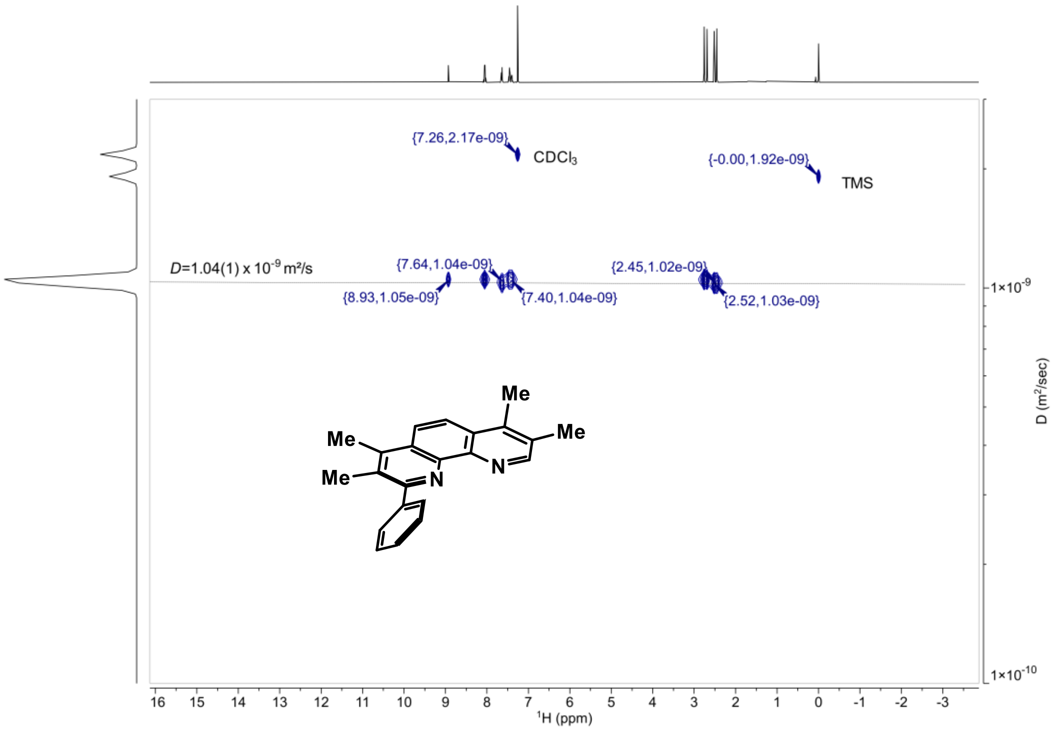

*Figure S46: 2D DOSY spectrum of Ligand **L3** in $CDCl_3$, 500 MHz, 298 K.*

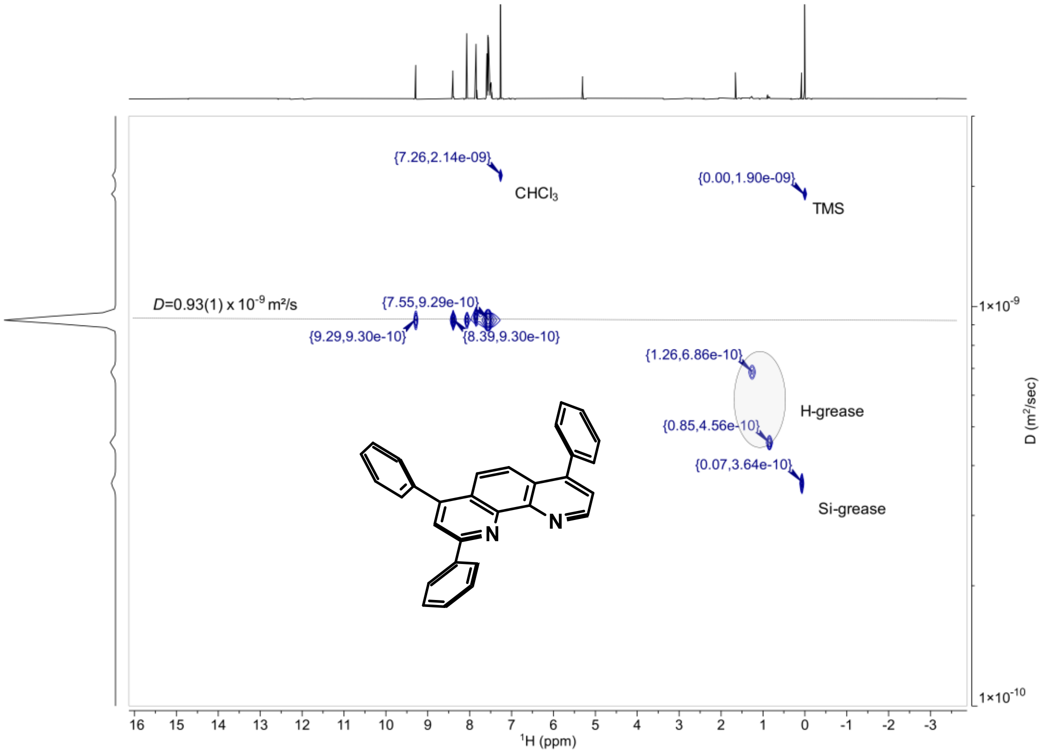

*Figure S47: 2D DOSY spectrum of Ligand **L4** in $CDCl_3$, 500 MHz, 298 K.*

5. Crystallographic Data

Single Crystal structure of 2

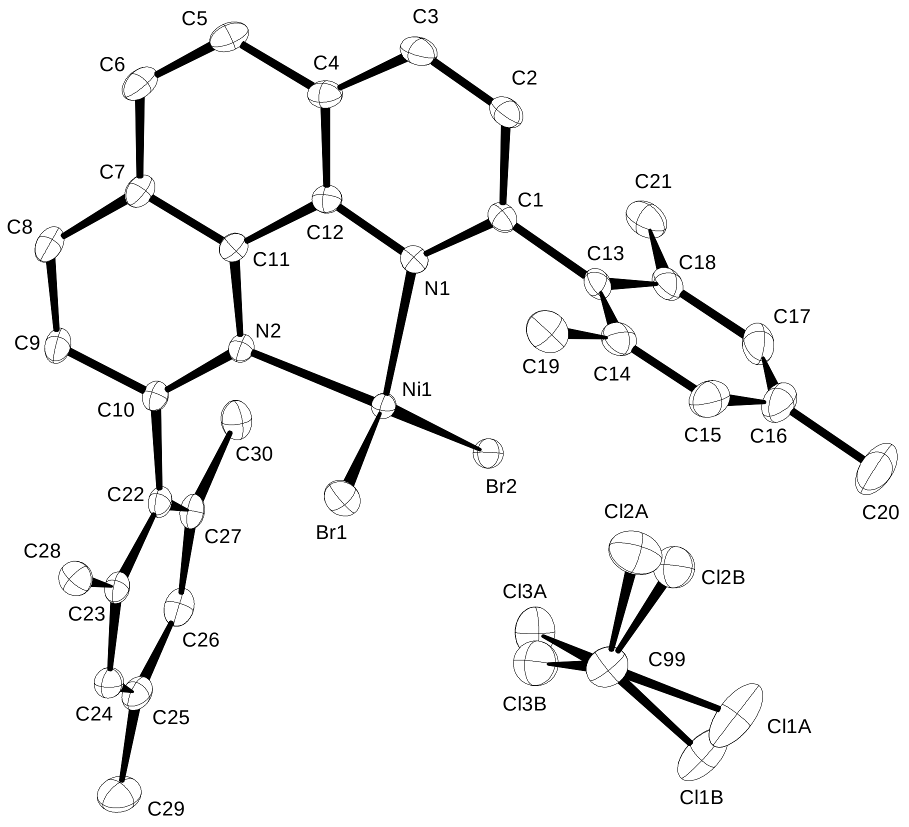

*Figure S48: The molecular structure of **2**; H atoms have been removed for clarity.*

X-Ray Crystal Structure Analysis of 2:

C_{31} H_{28} D Br_2 Cl_3 N_2 Ni, M_r = 755.45 g mol^{-1}, violet prism, crystal size 0.13 x 0.121 x 0.083 mm^3, Monoclinic, space group *C2c* [15], a = 25.8208(14) Å, b = 16.0862(9) Å, c = 19.4662(18) Å, β = 130.266(2)°, V = 6169.6(8) Å^3, T = 100(2) K, Z = 8, D_{calc} = 1.627 g·cm^3, λ = 0.71073 Å, $\mu(Mo\text{-}K_\alpha)$ = 3.503 mm^{-1}, Gaussian absorption correction ($T_{\min}$ = 0.68, $T_{\max}$ = 0.82), Bruker-AXS Kappa Mach3 with APEX-II detector and IµS microfocus Mo-anode X-ray source, 1.634 < θ < 33.831°, 141646 measured reflections, 12366 independent reflections, 10494 reflections with $I > 2\sigma(I)$, R_{int} = 0.0333. The structure was solved by *SHELXT* and refined by full-matrix least-squares (*SHELXL*) against F^2 to R_1 = 0.0237 [$I > 2\sigma(I)$], wR_2 = 0.0623 [all data], 375 parameters and 0 restraints.

Complete .cif data for the compound are available under the CSD number 2455391.

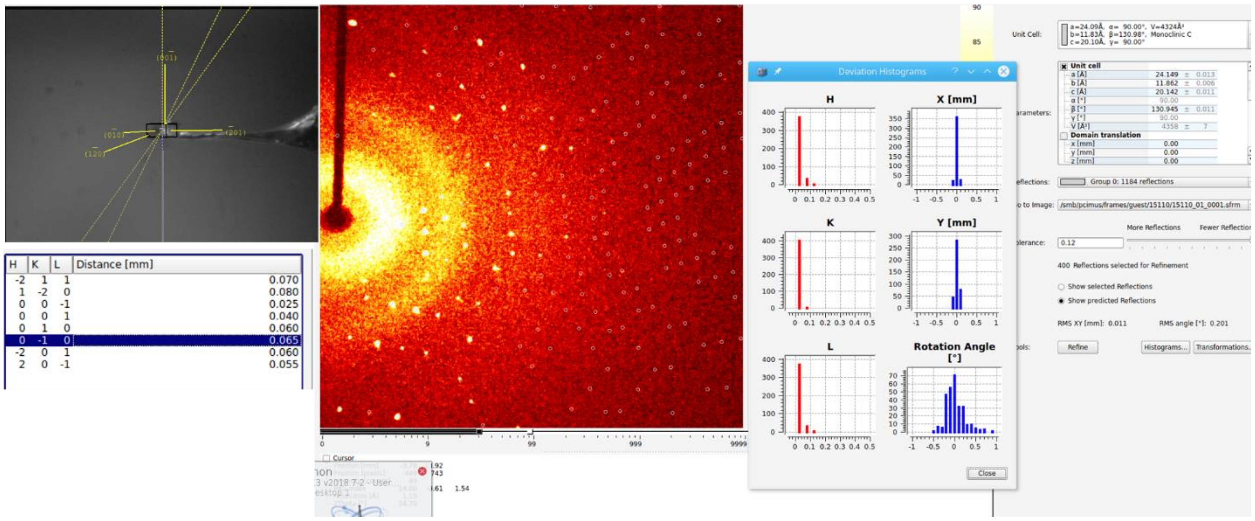

*Figure S49: Crystal faces and unit cell determination/refinement of **2**.*

*Table S3: Intensity statistics for dataset of the X-ray crystal structure analysis of **1**.*

Resolution	#Data	#Theory	%Compl.	Redund.	Mean I	Mean I/s	Rmerge	Rsigma
Inf - 2.68	192	192	100.0	17.89	103.57	108.50	0.0254	0.0075
2.68 - 1.76	454	454	100.0	20.15	62.20	108.88	0.0202	0.0073
1.76 - 1.39	634	634	100.0	20.82	39.24	94.57	0.0203	0.0077
1.39 - 1.21	654	654	100.0	20.53	29.18	87.28	0.0233	0.0082
1.21 - 1.10	615	615	100.0	19.43	23.77	76.76	0.0272	0.0091
1.10 - 1.02	637	637	100.0	15.16	18.52	59.46	0.0313	0.0116
1.02 - 0.96	639	639	100.0	12.70	17.00	50.53	0.0372	0.0138
0.96 - 0.91	653	653	100.0	11.22	12.44	39.88	0.0440	0.0172
0.91 - 0.87	637	637	100.0	10.22	10.75	34.65	0.0501	0.0206
0.87 - 0.83	764	764	100.0	9.69	8.61	30.23	0.0590	0.0247
0.83 - 0.80	658	658	100.0	9.36	8.56	28.06	0.0633	0.0268
0.80 - 0.78	528	528	100.0	8.94	6.71	23.23	0.0733	0.0330
0.78 - 0.75	871	871	100.0	8.68	6.64	21.76	0.0802	0.0356
0.75 - 0.73	649	649	100.0	8.41	5.89	19.49	0.0865	0.0411
0.73 - 0.72	355	355	100.0	8.14	5.07	16.92	0.0990	0.0487
0.72 - 0.70	788	788	100.0	7.89	5.04	16.02	0.0985	0.0511
0.70 - 0.68	875	875	100.0	7.66	4.34	13.82	0.1163	0.0614
0.68 - 0.67	478	478	100.0	7.31	4.19	13.18	0.1148	0.0661
0.67 - 0.66	502	502	100.0	7.33	3.42	11.38	0.1408	0.0801
0.66 - 0.65	530	530	100.0	7.13	3.06	9.93	0.1542	0.0903
0.65 - 0.64	622	692	89.9	4.74	2.46	7.06	0.1721	0.1443
0.74 - 0.64	4486	4556	98.5	7.21	4.09	13.09	0.1161	0.0677

*Table S4: Crystal data and structure refinement of **2**.*

Identification code	15115	
Empirical formula	$C_{31} H_{28} D Br_2 Cl_3 N_2 Ni$	
Color	violet	
Formula weight	755.45 g · mol^{-1}	
Temperature	100(2) K	
Wavelength	0.71073 Å	
Crystal system	MONOCLINIC	
Space group	**C2/c, (no. 15)**	
Unit cell dimensions	a = 25.8208(14) Å	α= 90°.
	b = 16.0862(9) Å	β= 130.266(2)°.
	c = 19.4662(18) Å	γ = 90°.
Volume	6169.6(8) Å3	
Z	8	
Density (calculated)	1.627 Mg · m^{-3}	
Absorption coefficient	3.503 mm-1	
F(000)	3024 e	
Crystal size	0.13 x 0.121 x 0.083 mm^3	
θ range for data collection	1.634 to 33.831°.	
Index ranges	$-40 \le h \le 40$, $-25 \le k \le 25$, $-30 \le l \le 30$	
Reflections collected	141646	
Independent reflections	12366 [R_{int} = 0.0333]	
Reflections with I>2σ(I)	10494	
Completeness to θ = 25.242°	100.0 %	
Absorption correction	Gaussian	
Max. and min. transmission	0.82 and 0.68	
Refinement method	Full-matrix least-squares on F^2	
Data / restraints / parameters	12366 / 0 / 375	
Goodness-of-fit on F2	1.029	
Final R indices [I>2σ(I)]	R_1 = 0.0237	wR_2 = 0.0581
R indices (all data)	R_1 = 0.0338	wR_2 = 0.0623
Largest diff. peak and hole	0.7 and -0.6 e · Å^{-3}	

Table S5: Bond lengths [Å] and angles [°] of ***2****.*

Br(1)-Ni(1)	2.3419(2)	Br(2)-Ni(1)	2.3519(3)
Ni(1)-N(1)	2.0054(10)	Ni(1)-N(2)	2.0075(9)
N(1)-C(1)	1.3353(14)	N(1)-C(12)	1.3619(14)
N(2)-C(10)	1.3330(14)	N(2)-C(11)	1.3666(15)
C(1)-C(2)	1.4076(17)	C(1)-C(13)	1.4878(16)
C(2)-C(3)	1.3718(18)	C(3)-C(4)	1.4053(17)
C(4)-C(5)	1.4348(17)	C(4)-C(12)	1.3990(16)
C(5)-C(6)	1.3525(19)	C(6)-C(7)	1.4339(17)
C(7)-C(8)	1.4072(17)	C(7)-C(11)	1.4010(16)
C(8)-C(9)	1.3686(18)	C(9)-C(10)	1.4071(15)
C(10)-C(22)	1.4891(16)	C(11)-C(12)	1.4316(16)
C(13)-C(14)	1.4005(18)	C(13)-C(18)	1.3997(18)
C(14)-C(15)	1.3936(19)	C(14)-C(19)	1.501(2)
C(15)-C(16)	1.384(2)	C(16)-C(17)	1.390(2)
C(16)-C(20)	1.512(2)	C(18)-C(21)	1.502(2)
C(22)-C(23)	1.4044(16)	C(22)-C(27)	1.4063(17)
C(23)-C(24)	1.3936(16)	C(23)-C(28)	1.5087(17)
C(24)-C(25)	1.3881(18)	C(25)-C(26)	1.3906(19)
C(25)-C(29)	1.5034(18)	C(26)-C(27)	1.3910(17)
C(27)-C(30)	1.5110(17)	Cl(1A)-C(99)	1.813(2)
Cl(1B)-C(99)	1.727(2)	Cl(2A)-C(99)	1.7534(16)
Cl(2B)-C(99)	1.701(4)	Cl(3A)-C(99)	1.7660(15)
Cl(3B)-C(99)	1.666(5)		
Br(1)-Ni(1)-Br(2)	130.200(8)	N(1)-Ni(1)-Br(1)	105.90(3)
N(1)-Ni(1)-Br(2)	111.08(3)	N(1)-Ni(1)-N(2)	83.86(4)
N(2)-Ni(1)-Br(1)	98.49(3)	N(2)-Ni(1)-Br(2)	117.26(3)
C(1)-N(1)-Ni(1)	130.20(8)	C(1)-N(1)-C(12)	118.96(10)
C(12)-N(1)-Ni(1)	110.78(7)	C(10)-N(2)-Ni(1)	130.16(8)
C(10)-N(2)-C(11)	118.74(9)	C(11)-N(2)-Ni(1)	110.38(7)
N(1)-C(1)-C(2)	121.04(11)	N(1)-C(1)-C(13)	118.86(10)
C(2)-C(1)-C(13)	120.10(10)	C(3)-C(2)-C(1)	120.28(11)
C(2)-C(3)-C(4)	119.37(11)	C(3)-C(4)-C(5)	123.17(11)
C(12)-C(4)-C(3)	117.32(11)	C(12)-C(4)-C(5)	119.51(11)
C(6)-C(5)-C(4)	120.76(11)	C(5)-C(6)-C(7)	120.70(11)
C(8)-C(7)-C(6)	123.35(11)	C(11)-C(7)-C(6)	119.53(11)
C(11)-C(7)-C(8)	117.11(11)	C(9)-C(8)-C(7)	119.17(11)
C(8)-C(9)-C(10)	120.75(11)	N(2)-C(10)-C(9)	120.94(11)
N(2)-C(10)-C(22)	121.33(9)	C(9)-C(10)-C(22)	117.65(10)
N(2)-C(11)-C(7)	123.16(10)	N(2)-C(11)-C(12)	117.23(10)
C(7)-C(11)-C(12)	119.61(10)	N(1)-C(12)-C(4)	123.02(10)
N(1)-C(12)-C(11)	117.24(10)	C(4)-C(12)-C(11)	119.74(10)
C(14)-C(13)-C(1)	119.54(11)	C(18)-C(13)-C(1)	118.86(11)
C(18)-C(13)-C(14)	121.46(11)	C(13)-C(14)-C(19)	121.00(12)
C(15)-C(14)-C(13)	118.17(13)	C(15)-C(14)-C(19)	120.81(13)
C(16)-C(15)-C(14)	121.76(14)	C(15)-C(16)-C(17)	118.80(13)
C(15)-C(16)-C(20)	120.50(17)	C(17)-C(16)-C(20)	120.70(17)
C(16)-C(17)-C(18)	121.68(14)	C(13)-C(18)-C(21)	120.72(11)
C(17)-C(18)-C(13)	118.06(13)	C(17)-C(18)-C(21)	121.21(13)
C(23)-C(22)-C(10)	119.99(10)	C(23)-C(22)-C(27)	120.67(11)
C(27)-C(22)-C(10)	118.52(10)	C(22)-C(23)-C(28)	122.03(11)

C(24)-C(23)-C(22)	118.61(11)	C(24)-C(23)-C(28)	119.31(11)
C(25)-C(24)-C(23)	121.76(12)	C(24)-C(25)-C(26)	118.47(11)
C(24)-C(25)-C(29)	120.86(12)	C(26)-C(25)-C(29)	120.62(12)
C(25)-C(26)-C(27)	121.96(12)	C(22)-C(27)-C(30)	121.63(11)
C(26)-C(27)-C(22)	118.45(11)	C(26)-C(27)-C(30)	119.87(11)
Cl(2A)-C(99)-Cl(1A)	102.55(10)	Cl(2A)-C(99)-Cl(3A)	108.37(8)
Cl(2B)-C(99)-Cl(1B)	104.22(14)	Cl(3A)-C(99)-Cl(1A)	111.95(10)
Cl(3B)-C(99)-Cl(1B)	100.45(18)	Cl(3B)-C(99)-Cl(2B)	127.92(19)

Single Crystal structure of 3

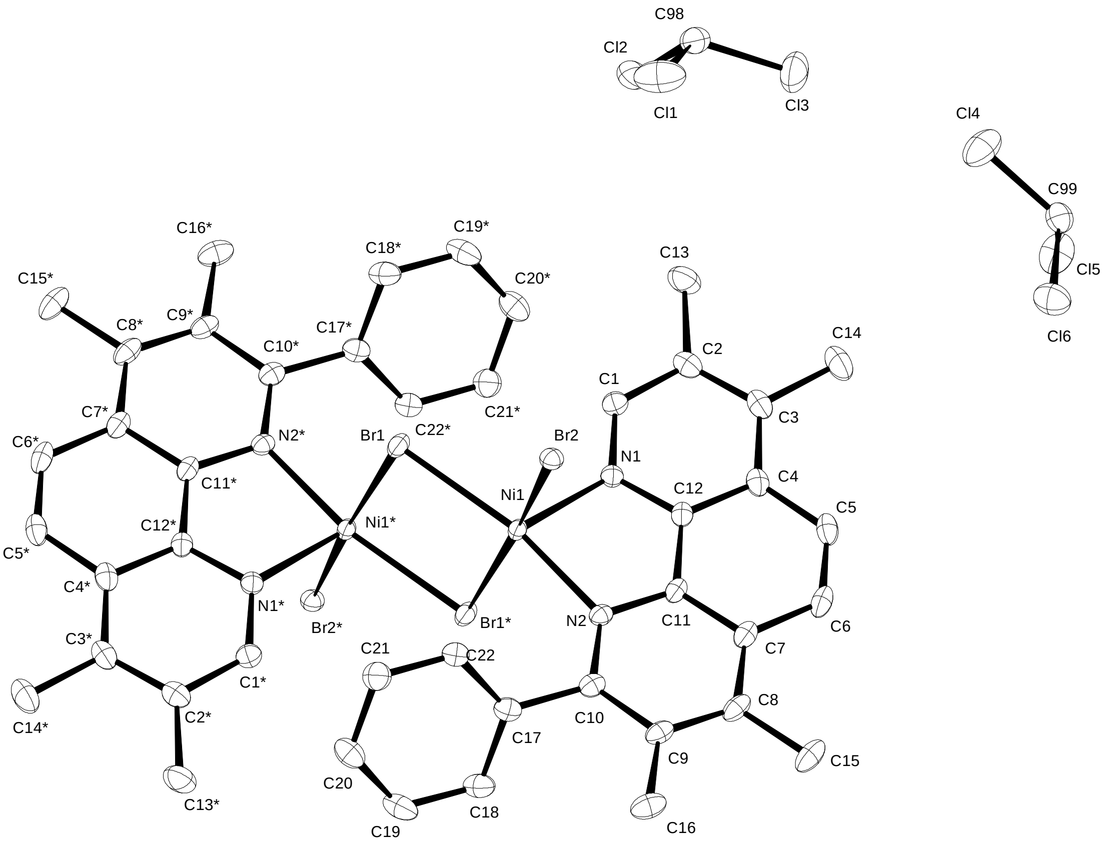

*Figure S50: The molecular structure of **3**; H atoms have been removed for clarity.*

X-Ray Crystal Structure Analysis of 3:

$C_{48}H_{40}Br_4Cl_{12}D_4N_4Ni_2$, M_r = 1543.35 g mol^{-1}, yellow needle, crystal size 0.165 x 0.041 x 0.021 mm^3, Triclinic, space group $P\bar{1}$ [2], a = 9.4494(4) Å, b = 11.1591(4) Å, c = 13.5558(5) Å, α = 89.832(2)°, β = 87.578(2)°, γ = 80.896(2)°, V = 1410.14(9) Å^3, T = 100(2) K, Z = 1, D_{calc} = 1.817 g·cm^3, λ = 0.71073 Å, $\mu(Mo\text{-}K_\alpha)$ = 4.108 mm^{-1}, Gaussian absorption correction ($T_{\min}$ = 0.53, $T_{\max}$ = 0.89), Bruker-AXS D8 VENTURE with APEX-III detector and IµS microfocus Mo-anode X-ray source, 2.185 < θ < 27.498°, 141213 measured reflections, 6483 independent reflections, 5948 reflections with $I > 2\sigma(I)$, R_{int} = 0.0468. The structure was solved by *SHELXT* and refined by full-matrix least-squares (*SHELXL*) against F^2 to R_1 = 0.0260 [$I > 2\sigma(I)$], wR_2 = 0.0669 [all data], 320 parameters and 0 restraints.

Complete .cif data for the compound are available under the CSD number 2455390.

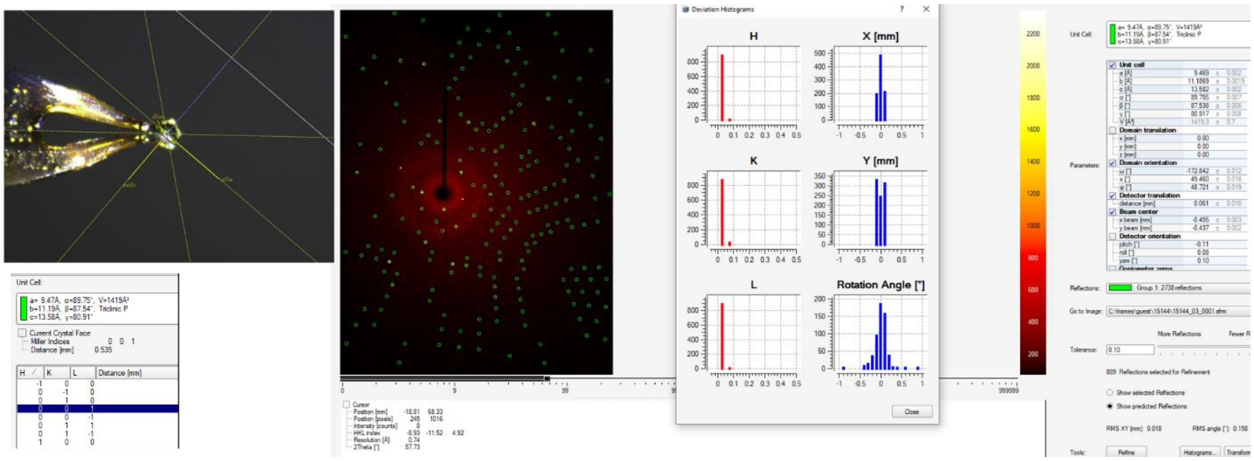

*Figure S51: Crystal faces and unit cell determination/refinement of **3**.*

*Table S6: Intensity statistics for dataset of the X-ray crystal structure analysis of **3**.*

Resolution	#Data	#Theory	%Compl.	Redund.	Mean I	Mean I/s	Rmerge	Rsigma
Inf - 2.62	163	164	99.4	23.12	128.09	95.30	0.0303	0.0188
2.62 - 1.76	376	376	100.0	28.35	89.55	96.73	0.0266	0.0119
1.76 - 1.40	538	538	100.0	30.39	54.44	88.30	0.0293	0.0085
1.40 - 1.22	546	546	100.0	29.31	34.99	67.76	0.0397	0.0097
1.22 - 1.10	594	594	100.0	26.66	37.14	65.33	0.0420	0.0104
1.10 - 1.03	476	476	100.0	22.65	25.08	49.27	0.0512	0.0139
1.03 - 0.97	543	543	100.0	20.76	20.85	40.95	0.0592	0.0165
0.97 - 0.92	547	547	100.0	19.38	16.68	34.01	0.0700	0.0202
0.92 - 0.88	536	536	100.0	18.45	14.25	29.89	0.0799	0.0236
0.88 - 0.84	649	649	100.0	17.49	13.89	27.78	0.0876	0.0258
0.84 - 0.81	585	585	100.0	16.44	12.54	24.80	0.1012	0.0296
0.81 - 0.79	418	418	100.0	16.29	11.10	22.32	0.1132	0.0333
0.79 - 0.77	501	501	100.0	16.14	10.35	20.65	0.1238	0.0361
0.77 - 0.75	530	530	100.0	14.98	8.34	16.69	0.1441	0.0448
0.75 - 0.73	585	585	100.0	14.19	7.69	15.10	0.1546	0.0505
0.73 - 0.71	655	655	100.0	14.31	7.08	13.93	0.1682	0.0549
0.71 - 0.70	367	367	100.0	13.91	7.03	13.48	0.1722	0.0576
0.70 - 0.68	769	769	100.0	13.50	5.61	10.80	0.1941	0.0705
0.68 - 0.67	429	429	100.0	13.12	4.39	8.92	0.2481	0.0916
0.67 - 0.66	471	471	100.0	12.35	4.26	8.23	0.2548	0.0991
0.66 - 0.65	493	554	89.0	10.46	4.47	8.13	0.2507	0.1071
0.75 - 0.65	3769	3830	98.4	13.16	5.87	11.39	0.1919	0.0697

*Table S7: Crystal data and structure refinement of **3**.*

Identification code	15144	
Empirical formula	$C_{48} H_{40} Br_4 Cl_{12} D_4 N_4 Ni_2$	
Color	yellow	
Formula weight	1543.35 g · mol^{-1}	
Temperature	100(2) K	
Wavelength	0.71073 Å	
Crystal system	TRICLINIC	
Space group	P$\bar{1}$, (no. 2)	
Unit cell dimensions	a = 9.4494(4) Å	α= 89.832(2)°.
	b = 11.1591(4) Å	β= 87.578(2)°.
	c = 13.5558(5) Å	γ = 80.896(2)°.
Volume	1410.14(9) Å^3	
Z	1	
Density (calculated)	1.817 Mg · m^{-3}	
Absorption coefficient	4.108 mm-1	
F(000)	760 e	
Crystal size	0.165 x 0.041 x 0.021 mm^3	
θ range for data collection	2.185 to 27.498°.	
Index ranges	$-12 \le h \le 12, -14\le k \le 14, -17 \le l \le 17$	
Reflections collected	141213	
Independent reflections	6483 [R_{int} = 0.0468]	
Reflections with I>2σ(I)	5948	
Completeness to θ = 25.242°	99.9 %	
Absorption correction	Semi-empirical from equivalents	
Max. and min. transmission	0.89 and 0.53	
Refinement method	Full-matrix least-squares on F^2	
Data / restraints / parameters	6483 / 0 / 320	
Goodness-of-fit on F2	1.042	
Final R indices [I>2σ(I)]	R_1 = 0.0260	wR^2 = 0.0650
R indices (all data)	R_1 = 0.0294	wR^2 = 0.0669
Largest diff. peak and hole	2.3 and -0.6 e · Å^{-3}	

*Table S8: Bond lengths [Å] and angles [°] of **3**.*

Br(1)-Ni(1)	2.4636(3)	Br(1)-Ni(1)*	2.5436(4)
Br(2)-Ni(1)	2.5309(3)	Ni(1)-N(1)	1.9970(19)
Ni(1)-N(2)	2.0324(19)	N(1)-C(1)	1.332(3)
N(1)-C(12)	1.354(3)	N(2)-C(10)	1.342(3)
N(2)-C(11)	1.355(3)	C(1)-C(2)	1.406(3)
C(2)-C(3)	1.386(4)	C(2)-C(13)	1.512(3)
C(3)-C(4)	1.418(3)	C(3)-C(14)	1.506(3)
C(4)-C(5)	1.433(3)	C(4)-C(12)	1.407(3)
C(5)-C(6)	1.359(4)	C(6)-C(7)	1.437(3)
C(7)-C(8)	1.418(3)	C(7)-C(11)	1.401(3)
C(8)-C(9)	1.393(3)	C(8)-C(15)	1.506(3)
C(9)-C(10)	1.425(3)	C(9)-C(16)	1.513(3)
C(10)-C(17)	1.486(3)	C(11)-C(12)	1.442(3)
C(17)-C(18)	1.398(3)	C(17)-C(22)	1.396(3)
C(18)-C(19)	1.387(4)	C(19)-C(20)	1.387(4)
C(20)-C(21)	1.389(4)	C(21)-C(22)	1.390(3)
Cl(1)-C(98)	1.752(3)	Cl(2)-C(98)	1.768(3)
Cl(3)-C(98)	1.754(3)	Cl(4)-C(99)	1.754(3)
Cl(5)-C(99)	1.759(3)	Cl(6)-C(99)	1.765(3)
Ni(1)-Br(1)-Ni(1)*	94.878(11)	Br(1)-Ni(1)-Br(1)*	85.123(11)
Br(1)-Ni(1)-Br(2)	93.102(11)	Br(2)-Ni(1)-Br(1)*	170.283(14)
N(1)-Ni(1)-Br(1)	107.92(6)	N(1)-Ni(1)-Br(1)*	100.90(5)
N(1)-Ni(1)-Br(2)	88.75(5)	N(1)-Ni(1)-N(2)	82.37(8)
N(2)-Ni(1)-Br(1)*	90.75(5)	N(2)-Ni(1)-Br(1)	169.46(6)
N(2)-Ni(1)-Br(2)	89.34(5)	C(1)-N(1)-Ni(1)	127.96(16)
C(1)-N(1)-C(12)	118.49(19)	C(12)-N(1)-Ni(1)	112.47(15)
C(10)-N(2)-Ni(1)	129.47(16)	C(10)-N(2)-C(11)	118.96(19)
C(11)-N(2)-Ni(1)	111.34(14)	N(1)-C(1)-C(2)	123.1(2)
C(1)-C(2)-C(13)	118.6(2)	C(3)-C(2)-C(1)	118.9(2)
C(3)-C(2)-C(13)	122.5(2)	C(2)-C(3)-C(4)	118.8(2)
C(2)-C(3)-C(14)	120.3(2)	C(4)-C(3)-C(14)	120.8(2)
C(3)-C(4)-C(5)	124.5(2)	C(12)-C(4)-C(3)	118.0(2)
C(12)-C(4)-C(5)	117.5(2)	C(6)-C(5)-C(4)	121.9(2)
C(5)-C(6)-C(7)	121.7(2)	C(8)-C(7)-C(6)	124.1(2)
C(11)-C(7)-C(6)	117.8(2)	C(11)-C(7)-C(8)	118.1(2)
C(7)-C(8)-C(15)	119.4(2)	C(9)-C(8)-C(7)	118.9(2)
C(9)-C(8)-C(15)	121.7(2)	C(8)-C(9)-C(10)	118.9(2)
C(8)-C(9)-C(16)	120.3(2)	C(10)-C(9)-C(16)	120.8(2)
N(2)-C(10)-C(9)	121.9(2)	N(2)-C(10)-C(17)	115.6(2)
C(9)-C(10)-C(17)	122.5(2)	N(2)-C(11)-C(7)	123.0(2)
N(2)-C(11)-C(12)	116.55(19)	C(7)-C(11)-C(12)	120.4(2)
N(1)-C(12)-C(4)	122.6(2)	N(1)-C(12)-C(11)	116.58(19)
C(4)-C(12)-C(11)	120.7(2)	C(18)-C(17)-C(10)	121.3(2)
C(22)-C(17)-C(10)	119.8(2)	C(22)-C(17)-C(18)	118.9(2)
C(19)-C(18)-C(17)	120.3(2)	C(20)-C(19)-C(18)	120.4(2)
C(19)-C(20)-C(21)	119.8(2)	C(22)-C(21)-C(20)	119.9(2)
C(21)-C(22)-C(17)	120.7(2)	Cl(1)-C(98)-Cl(2)	110.15(14)
Cl(1)-C(98)-Cl(3)	110.88(14)	Cl(3)-C(98)-Cl(2)	109.52(14)
Cl(4)-C(99)-Cl(5)	110.88(14)	Cl(4)-C(99)-Cl(6)	110.76(14)
Cl(5)-C(99)-Cl(6)	109.83(14)		

Symmetry transformations used to generate equivalent atoms: * -x+1,-y+2,-z+1

Single Crystal structure of 4

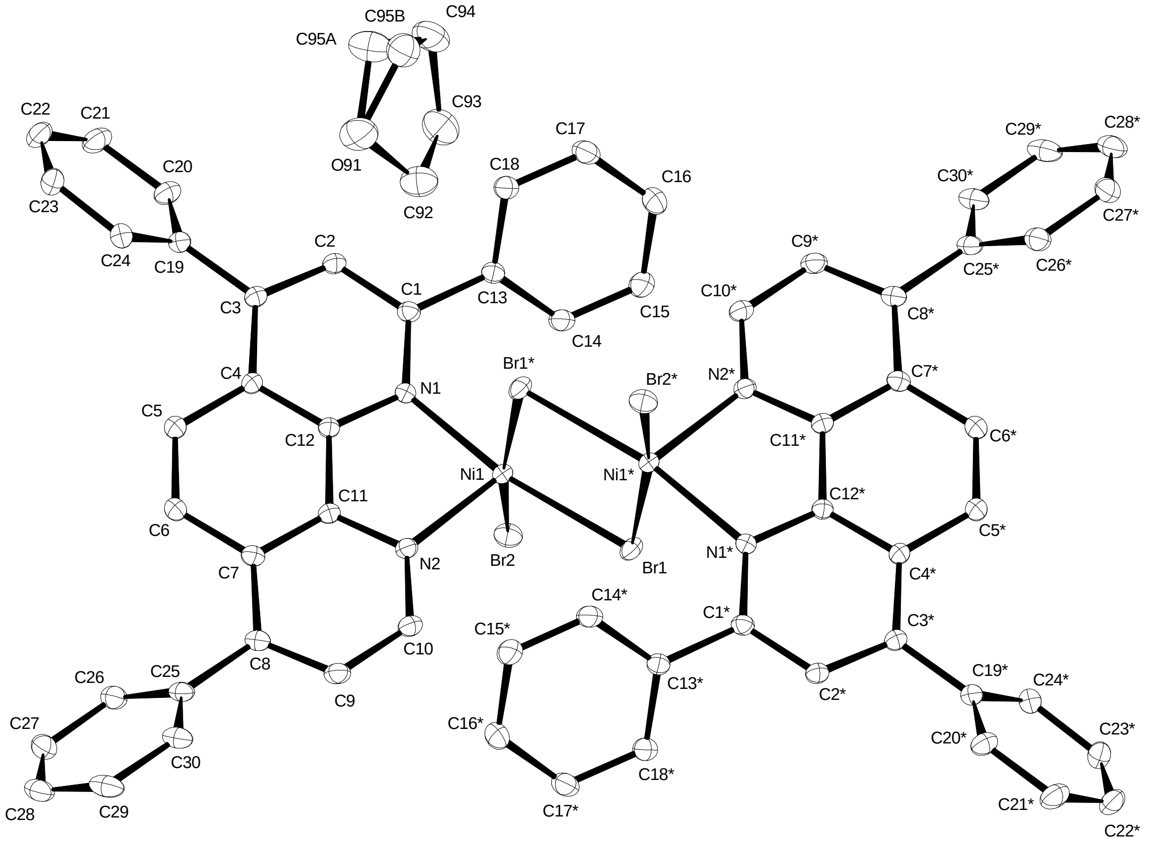

*Figure S52: The molecular structure of **4**; H atoms have been removed for clarity.*

X-Ray Crystal Structure Analysis of 4:

$C_{68}H_{56}Br_4N_4Ni_2O_2$, M_r = 1398.22 g mol^{-1}, yellow block, crystal size 0.118 x 0.089 x 0.085 mm^3, Triclinic, space group $P\bar{1}$ [2], a = 9.4810(6) Å, b = 10.0954(7) Å, c = 15.2154(11) Å, α = 75.630(3)°, β = 80.567(2)°, γ = 88.502(2)°, V = 1391.53(17) Å^3, T = 100(2) K, Z = 1, D_{calc} = 1.669 g·cm^3, λ = 0.71073 Å, $\mu(Mo\text{-}K_\alpha)$ = 3.600 mm^{-1}, Gaussian absorption correction ($T_{\min}$ = 0.66, $T_{\max}$ = 0.74), Bruker-AXS Kappa Mach3 with APEX-II detector and IµS microfocus Mo-anode X-ray source, 1.400 < θ < 33.142°, 51585 measured reflections, 10610 independent reflections, 9196 reflections with $I > 2\sigma(I)$, R_{int} = 0.0306. The structure was solved by *SHELXT* and refined by full-matrix least-squares (*SHELXL*) against F^2 to R_1 = 0.0261 [$I > 2\sigma(I)$], wR_2 = 0.0676 [all data], 365 parameters and 0 restraints.

Complete .cif data for the compound are available under the CSD number 2455387.

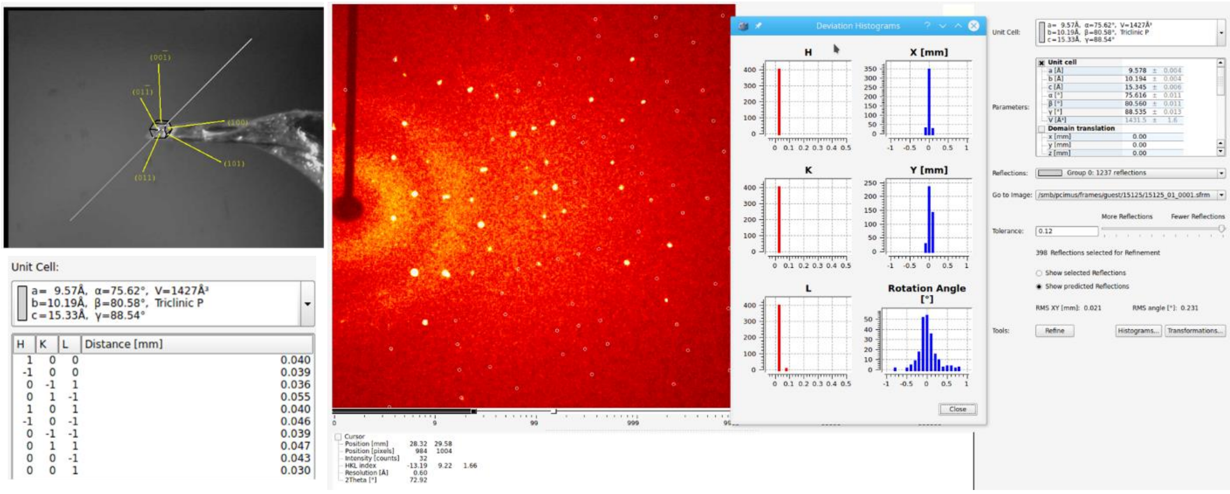

*Figure S53: Crystal faces and unit cell determination/refinement of **4**.*

*Table S9: Intensity statistics for dataset of the X-ray crystal structure analysis of **4**.*

Resolution	#Data	#Theory	%Compl.	Redund.	Mean I	Mean I/s	Rmerge	Rsigma
Inf - 2.44	199	199	100.0	9.12	96.81	68.77	0.0225	0.0127
2.44 - 1.64	461	461	100.0	9.37	64.57	63.68	0.0211	0.0128
1.64 - 1.31	643	643	100.0	9.30	33.38	56.01	0.0241	0.0138
1.31 - 1.14	665	665	100.0	8.93	30.64	52.47	0.0260	0.0146
1.14 - 1.04	626	626	100.0	7.22	24.87	43.16	0.0305	0.0174
1.04 - 0.96	693	693	100.0	5.40	18.11	32.64	0.0360	0.0228
0.96 - 0.90	721	721	100.0	4.52	12.66	25.95	0.0453	0.0286
0.90 - 0.86	576	576	100.0	3.96	11.98	22.63	0.0455	0.0322
0.86 - 0.82	702	702	100.0	3.85	10.86	21.15	0.0489	0.0352
0.82 - 0.79	612	612	100.0	3.62	10.89	19.93	0.0501	0.0381
0.79 - 0.76	732	732	100.0	3.53	9.01	17.42	0.0545	0.0440
0.76 - 0.74	567	567	100.0	3.29	7.05	14.38	0.0659	0.0549
0.74 - 0.72	589	590	99.8	3.29	6.54	13.66	0.0668	0.0596
0.72 - 0.70	722	724	99.7	3.11	6.68	12.79	0.0685	0.0620
0.70 - 0.68	738	743	99.3	3.01	5.38	11.02	0.0810	0.0763
0.68 - 0.66	866	876	98.9	2.88	4.61	9.65	0.0909	0.0886
0.66 - 0.65	498	502	99.2	2.82	4.06	8.39	0.0988	0.1014
0.65 - 0.64	459	473	97.0	2.64	3.63	7.73	0.1034	0.1145
0.64 - 0.62	1100	1130	97.3	2.62	3.59	7.50	0.1071	0.1200
0.62 - 0.61	802	916	87.6	2.16	3.49	6.75	0.1135	0.1368
0.71 - 0.61	4815	4993	96.4	2.69	4.31	8.78	0.0930	0.0990

*Table S10: Crystal data and structure refinement of **4**.*

Identification code	15125	
Empirical formula	$C_{68} H_{56} Br_4 N_4 Ni_2 O_2$	
Color	yellow	
Formula weight	1398.22 g·mol^{-1}	
Temperature	100(2) K	
Wavelength	0.71073 Å	
Crystal system	TRICLINIC	
Space group	**P$\bar{1}$, (no. 2)**	
Unit cell dimensions	a = 9.4810(6) Å	α= 75.630(3)°.
	b = 10.0954(7) Å	β= 80.567(2)°.
	c = 15.2154(11) Å	γ = 88.502(2)°.
Volume	1391.53(17) Å^3	
Z	1	
Density (calculated)	1.669 Mg · m^{-3}	
Absorption coefficient	3.600 mm^{-1}	
F(000)	704 e	
Crystal size	0.118 x 0.089 x 0.085 mm^3	
θ range for data collection	1.400 to 33.142°	
Index ranges	$-14 \leq h \leq 14$, $-14 \leq k \leq 15$, $-23 \leq l \leq 23$	
Reflections collected	51585	
Independent reflections	10610 [Rint = 0.0306]	
Reflections with I>2σ(I)	9196	
Completeness to θ = 25.242°	100.0 %	
Absorption correction	Gaussian	
Max. and min. transmission	0.74 and 0.66	
Refinement method	Full-matrix least-squares on F^2	
Data / restraints / parameters	10610 / 0 / 365	
Goodness-of-fit on F2	1.028	
Final R indices [I>2σ(I)]	R_1 = 0.0261	wR^2 = 0.0651
R indices (all data)	R_1 = 0.0333	wR^2 = 0.0676
Largest diff. peak and hole	1.2 and -0.5 e · Å^{-3}	

*Table S11: Bond lengths [Å] and angles [°] of **4**.*

Br(1)-Ni(1)	2.4537(3)	Br(1)-Ni(1)[*]	2.5405(2)
Br(2)-Ni(1)	2.5076(2)	Ni(1)-N(1)	2.0434(11)
Ni(1)-N(2)	2.0024(11)	N(1)-C(1)	1.3394(16)
N(1)-C(12)	1.3578(16)	N(2)-C(10)	1.3292(16)
N(2)-C(11)	1.3591(18)	C(1)-C(2)	1.4083(19)
C(1)-C(13)	1.4806(18)	C(2)-C(3)	1.3794(18)
C(3)-C(4)	1.4268(17)	C(3)-C(19)	1.4851(19)
C(4)-C(5)	1.4308(18)	C(4)-C(12)	1.4065(19)
C(5)-C(6)	1.3590(18)	C(6)-C(7)	1.435(2)
C(7)-C(8)	1.4290(17)	C(7)-C(11)	1.4037(18)
C(8)-C(9)	1.382(2)	C(8)-C(25)	1.4817(18)
C(9)-C(10)	1.4009(19)	C(11)-C(12)	1.4432(17)
C(13)-C(14)	1.396(2)	C(13)-C(18)	1.3984(18)
C(14)-C(15)	1.384(2)	C(15)-C(16)	1.388(2)
C(16)-C(17)	1.386(2)	C(17)-C(18)	1.383(2)
C(19)-C(20)	1.3955(19)	C(19)-C(24)	1.402(2)
C(20)-C(21)	1.391(2)	C(21)-C(22)	1.386(2)
C(22)-C(23)	1.391(2)	C(23)-C(24)	1.388(2)
C(25)-C(26)	1.396(2)	C(25)-C(30)	1.3975(19)
C(26)-C(27)	1.383(2)	C(27)-C(28)	1.387(2)
C(28)-C(29)	1.383(2)	C(29)-C(30)	1.391(2)
O(91)-C(92)	1.427(2)	O(91)-C(95A)	1.405(3)
O(91)-C(95B)	1.497(8)	C(92)-C(93)	1.509(2)
C(93)-C(94)	1.543(3)	C(94)-C(95A)	1.551(4)
C(94)-C(95B)	1.453(8)		
Ni(1)-Br(1)-Ni(1)[*]	93.788(9)	Br(1)-Ni(1)-Br(1)[*]	86.212(8)
Br(1)-Ni(1)-Br(2)	92.345(9)	Br(2)-Ni(1)-Br(1)[*]	167.124(9)
N(1)-Ni(1)-Br(1)	169.59(3)	N(1)-Ni(1)-Br(1)[*]	85.35(3)
N(1)-Ni(1)-Br(2)	94.52(3)	N(2)-Ni(1)-Br(1)	105.38(3)
N(2)-Ni(1)-Br(1)[*]	98.92(3)	N(2)-Ni(1)-Br(2)	93.81(3)
N(2)-Ni(1)-N(1)	81.98(4)	C(1)-N(1)-Ni(1)	129.14(9)
C(1)-N(1)-C(12)	118.88(11)	C(12)-N(1)-Ni(1)	111.66(8)
C(10)-N(2)-Ni(1)	128.31(10)	C(10)-N(2)-C(11)	118.36(12)
C(11)-N(2)-Ni(1)	113.28(8)	N(1)-C(1)-C(2)	120.86(12)
N(1)-C(1)-C(13)	118.16(12)	C(2)-C(1)-C(13)	120.85(11)
C(3)-C(2)-C(1)	121.56(12)	C(2)-C(3)-C(4)	117.68(12)
C(2)-C(3)-C(19)	119.44(11)	C(4)-C(3)-C(19)	122.88(12)
C(3)-C(4)-C(5)	124.17(12)	C(12)-C(4)-C(3)	117.52(12)
C(12)-C(4)-C(5)	118.29(11)	C(6)-C(5)-C(4)	121.67(13)
C(5)-C(6)-C(7)	121.56(12)	C(8)-C(7)-C(6)	124.17(12)
C(11)-C(7)-C(6)	117.79(11)	C(11)-C(7)-C(8)	117.90(12)
C(7)-C(8)-C(25)	122.70(12)	C(9)-C(8)-C(7)	117.50(12)
C(9)-C(8)-C(25)	119.77(11)	C(8)-C(9)-C(10)	120.68(12)
N(2)-C(10)-C(9)	122.36(13)	N(2)-C(11)-C(7)	123.13(11)
N(2)-C(11)-C(12)	116.03(11)	C(7)-C(11)-C(12)	120.79(12)
N(1)-C(12)-C(4)	123.46(11)	N(1)-C(12)-C(11)	116.77(12)
C(4)-C(12)-C(11)	119.71(11)	C(14)-C(13)-C(1)	121.13(12)
C(14)-C(13)-C(18)	118.50(13)	C(18)-C(13)-C(1)	120.29(12)
C(15)-C(14)-C(13)	120.39(13)	C(14)-C(15)-C(16)	120.56(14)
C(17)-C(16)-C(15)	119.45(14)	C(18)-C(17)-C(16)	120.22(13)

C(17)-C(18)-C(13)	120.81(13)	C(20)-C(19)-C(3)	119.38(12)
C(20)-C(19)-C(24)	119.21(13)	C(24)-C(19)-C(3)	121.35(12)
C(21)-C(20)-C(19)	120.08(14)	C(22)-C(21)-C(20)	120.52(14)
C(21)-C(22)-C(23)	119.71(14)	C(24)-C(23)-C(22)	120.26(14)
C(23)-C(24)-C(19)	120.19(13)	C(26)-C(25)-C(8)	121.71(12)
C(26)-C(25)-C(30)	118.60(12)	C(30)-C(25)-C(8)	119.66(13)
C(27)-C(26)-C(25)	120.53(13)	C(26)-C(27)-C(28)	120.58(15)
C(29)-C(28)-C(27)	119.43(14)	C(28)-C(29)-C(30)	120.40(14)
C(29)-C(30)-C(25)	120.42(14)	C(92)-O(91)-C(95B)	98.4(3)
C(95A)-O(91)-C(92)	111.29(18)	C(92)-C(93)-C(94)	102.50(15)
C(93)-C(94)-C(95A)	104.66(16)	C(95B)-C(94)-C(93)	103.7(3)
O(91)-C(95A)-C(94)	106.1(2)	C(94)-C(95B)-O(91)	106.5(5)

Symmetry transformations used to generate equivalent atoms: * -x+1,-y,-z+2

Single Crystal structure of 6

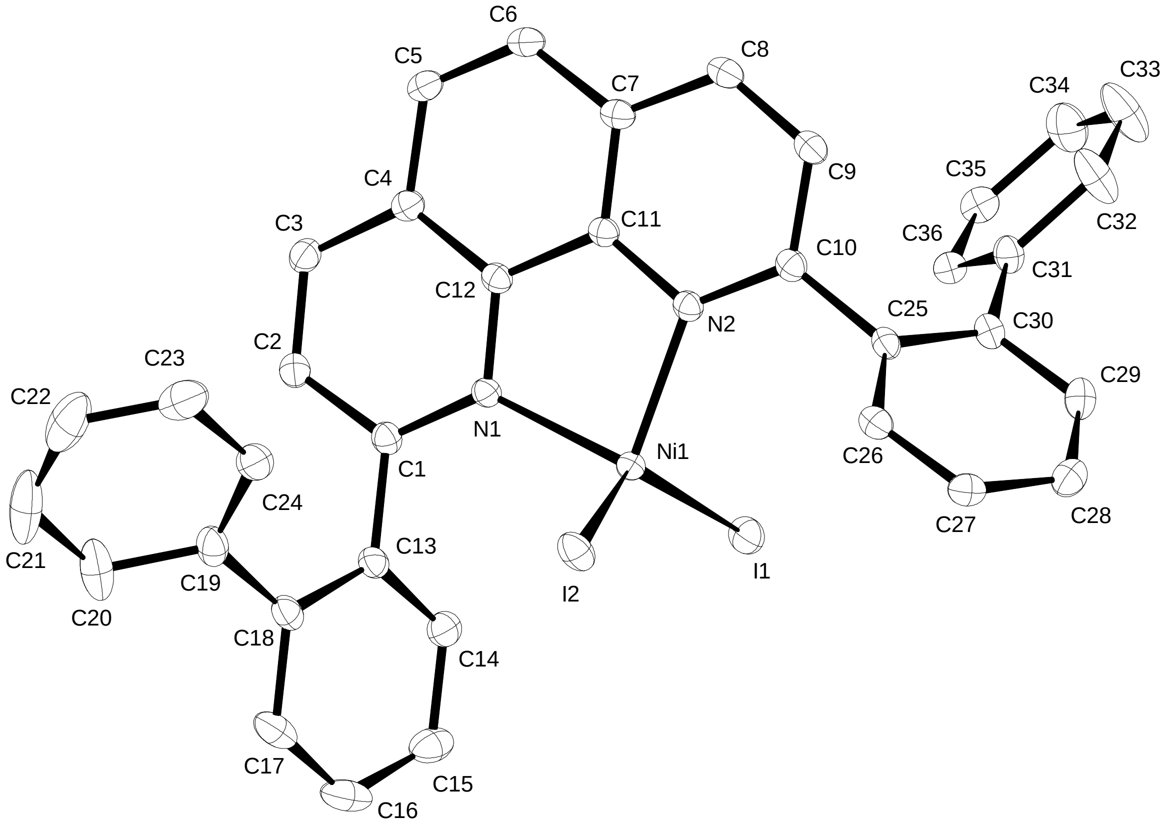

*Figure S54: The molecular structure of **6**; H atoms have been removed for clarity.*

X-Ray Crystal Structure Analysis of 6:

$C_{36}H_{24}I_2N_2Ni$, M_r = 797.08 g mol^{-1}, translucent dark orange plate, crystal size 0.103 x 0.064 x 0.021 mm^3, Monoclinic, space group *P21/n* [14], a = 7.488(10) Å, b = 16.102(19) Å, c = 24.88(3) Å, β = 96.15(3)°, V = 2982(6) Å^3, T = 100(2) K, Z = 4, D_{calc} = 1.775 g·cm^3, λ = 0.71073 Å, μ(*Mo-K$_\alpha$*) = 2.749 mm^{-1}, Gaussian absorption correction ($T_{\min}$ = 0.86, $T_{\max}$ = 1.00), Bruker-AXS D8 VENTURE with APEX-III detector and IµS microfocus Mo-anode X-ray source, 2.530 < θ < 31.007°, 307814 measured reflections, 9498 independent reflections, 8609 reflections with $I > 2\sigma(I)$, R_{int} = 0.0447. The structure was solved by *SHELXT* and refined by full-matrix least-squares (*SHELXL*) against F^2 to R_1 = 0.0197 [$I > 2\sigma(I)$], wR_2 = 0.0502 [all data], 370 parameters and 0 restraints.

Complete .cif data for the compound are available under the CSD number 2455388.

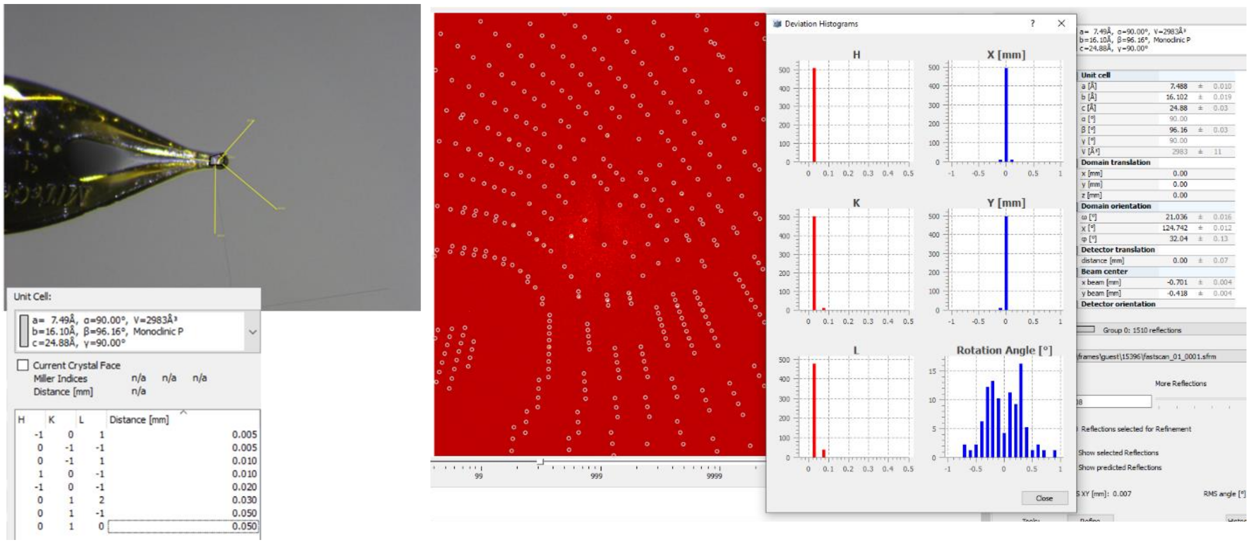

*Figure S55: Crystal faces and unit cell determination/refinement of **6**.*

*Table S12: Intensity statistics for dataset of the X-ray crystal structure analysis of **6**.*

Resolution	#Data	#Theory	%Compl.	Redund.	Mean I	Mean I/s	Rmerge	Rsigma
Inf - 2.88	148	153	96.7	45.84	121.88	129.50	0.0211	0.0126
2.88 - 1.89	350	350	100.0	55.63	88.85	135.81	0.0213	0.0053
1.89 - 1.49	496	496	100.0	55.26	54.53	116.22	0.0289	0.0058
1.49 - 1.31	478	478	100.0	47.86	41.48	96.70	0.0364	0.0069
1.31 - 1.18	536	536	100.0	39.83	33.47	80.44	0.0405	0.0084
1.18 - 1.10	453	453	100.0	37.97	31.18	75.00	0.0419	0.0089
1.10 - 1.03	534	534	100.0	34.77	25.89	65.03	0.0469	0.0104
1.03 - 0.98	485	485	100.0	33.05	19.38	54.98	0.0563	0.0124
0.98 - 0.93	553	553	100.0	32.01	15.86	48.14	0.0645	0.0143
0.93 - 0.90	431	431	100.0	30.26	14.49	43.09	0.0700	0.0160
0.90 - 0.87	475	475	100.0	30.91	14.56	44.33	0.0729	0.0163
0.87 - 0.84	517	517	100.0	30.35	12.47	38.06	0.0796	0.0181
0.84 - 0.81	638	638	100.0	26.71	11.27	32.35	0.0884	0.0217
0.81 - 0.79	475	475	100.0	25.94	8.93	27.61	0.1017	0.0256
0.79 - 0.77	507	507	100.0	25.29	8.96	25.49	0.1044	0.0271
0.77 - 0.75	571	571	100.0	24.66	8.88	25.37	0.1061	0.0280
0.75 - 0.74	306	306	100.0	23.75	7.45	21.14	0.1186	0.0330
0.74 - 0.72	698	698	100.0	21.80	6.87	19.01	0.1354	0.0390
0.72 - 0.71	361	361	100.0	21.46	6.21	17.04	0.1465	0.0425
0.71 - 0.70	399	399	100.0	21.11	6.02	16.63	0.1502	0.0443
0.70 - 0.69	402	410	98.0	18.80	4.70	12.61	0.1785	0.0595
0.79 - 0.69	3244	3252	99.8	22.53	7.16	20.04	0.1253	0.0362

Table S13: Crystal data and structure refinement of ***6****.*

Identification code	15396	
Empirical formula	$C_{36} H_{24} I_2 N_2 Ni$	
Color	translucent dark orange	
Formula weight	797.08 g · mol^{-1}	
Temperature	100(2) K	
Wavelength	0.71073 Å	
Crystal system	MONOCLINIC	
Space group	**$P2_1/n$, (no. 14)**	
Unit cell dimensions	a = 7.488(10) Å	α= 90°.
	b = 16.102(19) Å	β= 96.15(3)°.
	c = 24.88(3) Å	γ = 90°.
Volume	2982(6) Å^3	
Z	4	
Density (calculated)	1.775 Mg · m^{-3}	
Absorption coefficient	2.749 mm^{-1}	
F(000)	1552 e	
Crystal size	0.103 x 0.064 x 0.021 mm^3	
θ range for data collection	2.530 to 31.007°.	
Index ranges	$-10 \leq h \leq 10$, $-23 \leq k \leq 23$, $-35 \leq l \leq 36$	
Reflections collected	307814	
Independent reflections	9498 [R_{int} = 0.0447]	
Reflections with $I>2\sigma(I)$	8609	
Completeness to θ = 25.242°	99.9 %	
Absorption correction	Numerical	
Max. and min. transmission	1.00 and 0.86	
Refinement method	Full-matrix least-squares on F^2	
Data / restraints / parameters	9498 / 0 / 370	
Goodness-of-fit on F2	1.021	
Final R indices [I>2□(I)]	R_1 = 0.0197	wR^2 = 0.0483
R indices (all data)	R_1 = 0.0236	wR^2 = 0.0502
Largest diff. peak and hole	1.0 and -0.6 e · Å^{-3}	

*Table S14: Bond lengths [Å] and angles [°] of **6**.*

I(1)-Ni(1)	2.531(2)	I(2)-Ni(1)	2.540(3)
Ni(1)-N(1)	2.008(2)	Ni(1)-N(2)	2.024(3)
N(1)-C(1)	1.338(2)	N(1)-C(12)	1.367(2)
N(2)-C(10)	1.340(2)	N(2)-C(11)	1.368(2)
C(1)-C(2)	1.413(2)	C(1)-C(13)	1.485(2)
C(2)-C(3)	1.368(3)	C(3)-C(4)	1.413(2)
C(4)-C(5)	1.433(2)	C(4)-C(12)	1.407(2)
C(5)-C(6)	1.357(2)	C(6)-C(7)	1.431(3)
C(7)-C(8)	1.412(2)	C(7)-C(11)	1.406(2)
C(8)-C(9)	1.368(3)	C(9)-C(10)	1.416(2)
C(10)-C(25)	1.485(3)	C(11)-C(12)	1.436(2)
C(13)-C(14)	1.394(3)	C(13)-C(18)	1.412(2)
C(14)-C(15)	1.387(3)	C(15)-C(16)	1.388(3)
C(16)-C(17)	1.384(3)	C(17)-C(18)	1.401(3)
C(18)-C(19)	1.488(3)	C(19)-C(20)	1.400(3)
C(19)-C(24)	1.399(3)	C(20)-C(21)	1.391(3)
C(21)-C(22)	1.382(4)	C(22)-C(23)	1.389(3)
C(23)-C(24)	1.393(3)	C(25)-C(26)	1.400(2)
C(25)-C(30)	1.408(2)	C(26)-C(27)	1.387(3)
C(27)-C(28)	1.388(3)	C(28)-C(29)	1.387(3)
C(29)-C(30)	1.398(3)	C(30)-C(31)	1.493(2)
C(31)-C(32)	1.394(3)	C(31)-C(36)	1.392(3)
C(32)-C(33)	1.390(3)	C(33)-C(34)	1.386(3)
C(34)-C(35)	1.387(3)	C(35)-C(36)	1.393(3)
I(1)-Ni(1)-I(2)	133.77(5)	N(1)-Ni(1)-I(1)	112.68(10)
N(1)-Ni(1)-I(2)	101.68(6)	N(1)-Ni(1)-N(2)	84.05(5)
N(2)-Ni(1)-I(1)	99.82(4)	N(2)-Ni(1)-I(2)	114.06(6)
C(1)-N(1)-Ni(1)	130.24(11)	C(1)-N(1)-C(12)	119.01(14)
C(12)-N(1)-Ni(1)	110.70(10)	C(10)-N(2)-Ni(1)	131.17(12)
C(10)-N(2)-C(11)	118.57(14)	C(11)-N(2)-Ni(1)	110.20(9)
N(1)-C(1)-C(2)	121.00(14)	N(1)-C(1)-C(13)	119.10(15)
C(2)-C(1)-C(13)	119.90(13)	C(3)-C(2)-C(1)	120.45(14)
C(2)-C(3)-C(4)	119.40(15)	C(3)-C(4)-C(5)	123.29(15)
C(12)-C(4)-C(3)	117.28(14)	C(12)-C(4)-C(5)	119.42(13)
C(6)-C(5)-C(4)	120.99(15)	C(5)-C(6)-C(7)	120.61(14)
C(8)-C(7)-C(6)	122.80(14)	C(11)-C(7)-C(6)	119.91(13)
C(11)-C(7)-C(8)	117.27(15)	C(9)-C(8)-C(7)	119.15(15)
C(8)-C(9)-C(10)	120.68(13)	N(2)-C(10)-C(9)	120.98(15)
N(2)-C(10)-C(25)	120.20(14)	C(9)-C(10)-C(25)	118.79(13)
N(2)-C(11)-C(7)	123.19(13)	N(2)-C(11)-C(12)	117.45(14)
C(7)-C(11)-C(12)	119.35(15)	N(1)-C(12)-C(4)	122.74(13)
N(1)-C(12)-C(11)	117.55(15)	C(4)-C(12)-C(11)	119.72(14)
C(14)-C(13)-C(1)	118.51(13)	C(14)-C(13)-C(18)	120.31(15)
C(18)-C(13)-C(1)	121.09(15)	C(15)-C(14)-C(13)	120.53(15)
C(14)-C(15)-C(16)	119.63(17)	C(17)-C(16)-C(15)	120.32(16)
C(16)-C(17)-C(18)	121.25(15)	C(13)-C(18)-C(19)	122.49(15)
C(17)-C(18)-C(13)	117.95(16)	C(17)-C(18)-C(19)	119.54(14)
C(20)-C(19)-C(18)	119.98(17)	C(24)-C(19)-C(18)	121.56(14)
C(24)-C(19)-C(20)	118.43(17)	C(21)-C(20)-C(19)	120.3(2)
C(22)-C(21)-C(20)	120.77(19)	C(21)-C(22)-C(23)	119.7(2)

C(22)-C(23)-C(24)	119.9(2)	C(23)-C(24)-C(19)	120.90(17)
C(26)-C(25)-C(10)	119.74(13)	C(26)-C(25)-C(30)	119.55(14)
C(30)-C(25)-C(10)	120.49(15)	C(27)-C(26)-C(25)	120.72(14)
C(26)-C(27)-C(28)	119.99(16)	C(29)-C(28)-C(27)	119.69(15)
C(28)-C(29)-C(30)	121.41(15)	C(25)-C(30)-C(31)	124.51(14)
C(29)-C(30)-C(25)	118.63(16)	C(29)-C(30)-C(31)	116.79(14)
C(32)-C(31)-C(30)	119.20(15)	C(36)-C(31)-C(30)	121.51(15)
C(36)-C(31)-C(32)	118.87(15)	C(33)-C(32)-C(31)	120.61(18)
C(34)-C(33)-C(32)	120.29(19)	C(33)-C(34)-C(35)	119.46(16)
C(34)-C(35)-C(36)	120.42(16)	C(31)-C(36)-C(35)	120.34(16)

Single Crystal structure of 7

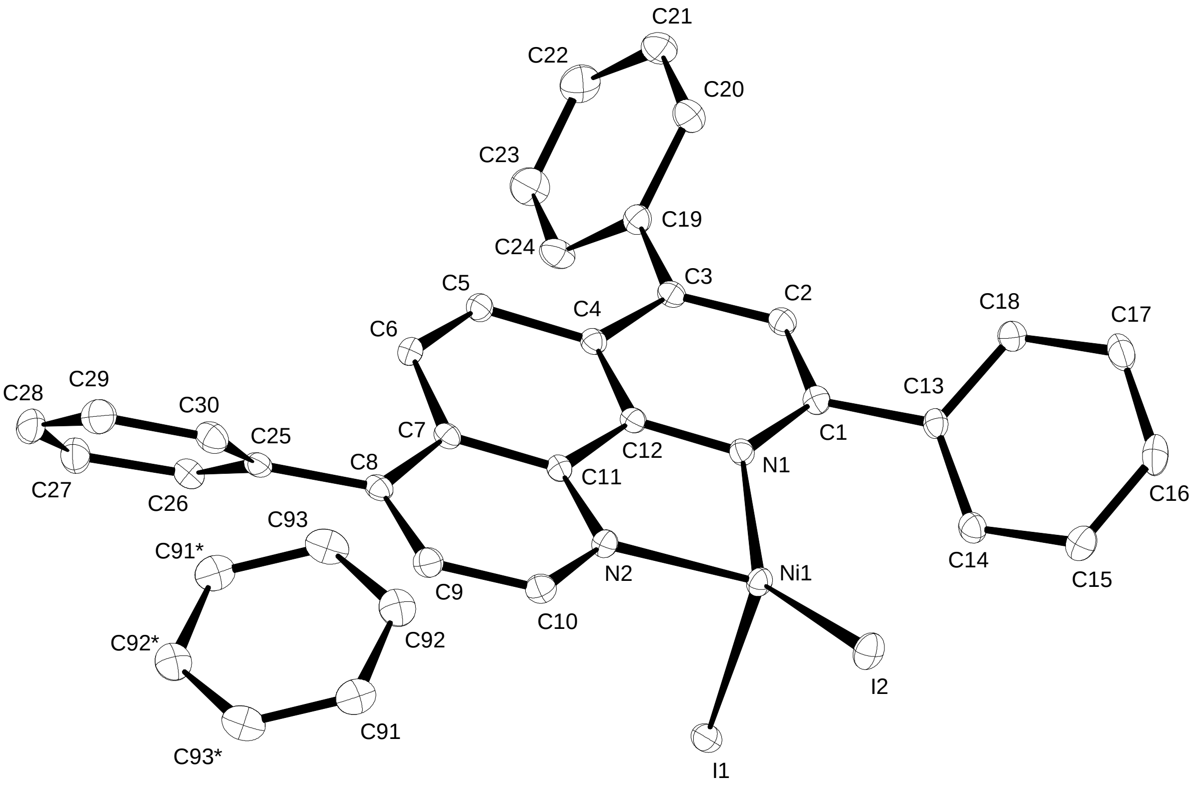

*Figure S56: The molecular structure of **7**; H atoms have been removed for clarity.*

X-Ray Crystal Structure Analysis of 7:

C_{33} H_{23} I_2 N_2 Ni, M_r = 760.04 g mol^{-1}, orange plate, crystal size 0.117 x 0.095 x 0.05 mm^3, Monoclinic, space group *P21/c* [14], a = 11.8061(6) Å, b = 25.6066(13) Å, c = 9.2124(5) Å, β = 99.787(2)°, V = 2744.5(2) Å^3, T = 100(2) K, Z = 4, D_{calc} = 1.839 g·cm^3, λ = 0.71073 Å, μ(*Mo-K$_\alpha$*) = 2.982 mm^{-1}, Gaussian absorption correction ($T_{\min}$ = 0.77, $T_{\max}$ = 0.89), Bruker-AXS Kappa Mach3 with APEX-II detector and IµS microfocus Mo-anode X-ray source, 1.590 < θ < 32.344°, 94489 measured reflections, 9731 independent reflections, 7927 reflections with $I > 2\sigma(I)$, R_{int} = 0.0483. The structure was solved by *SHELXT* and refined by full-matrix least-squares (*SHELXL*) against F^2 to R_1 = 0.0247 [$I > 2\sigma(I)$], wR_2 = 0.0496 [all data], 343 parameters and 0 restraints.

Complete .cif data for the compound are available under the CSD number 2455389.

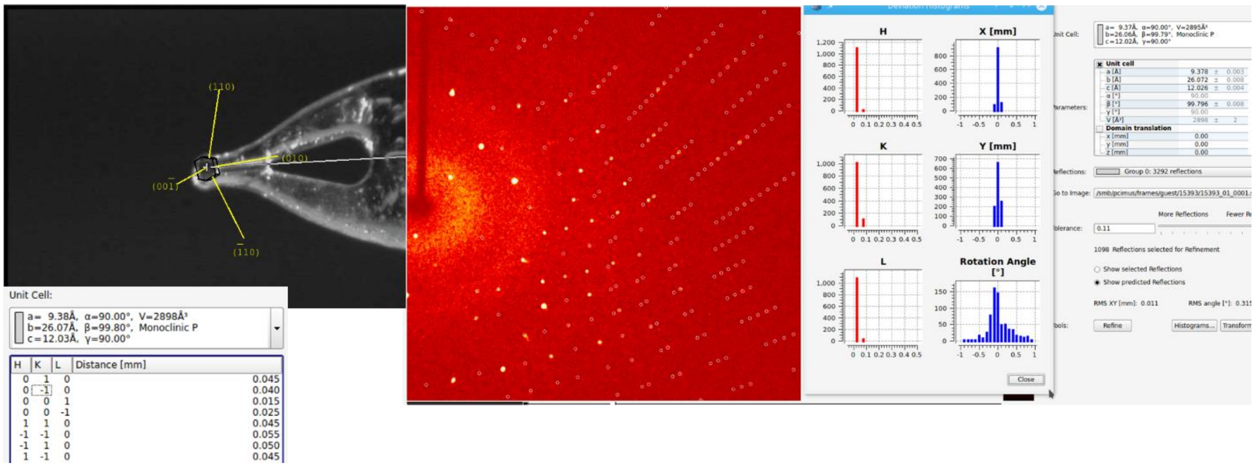

Figure S57: Crystal faces and unit cell determination/refinement of ***7****.*

Table S15: Intensity statistics for dataset of the X-ray crystal structure analysis of ***7****.*

Resolution	#Data	#Theory	%Compl.	Redund.	Mean I	Mean I/s	Rmerge	Rsigma
Inf - 2.78	149	150	99.3	16.37	86.92	99.02	0.0203	0.0076
2.78 - 1.82	354	354	100.0	17.80	62.42	86.81	0.0243	0.0082
1.82 - 1.44	511	511	100.0	17.99	40.01	69.37	0.0299	0.0098
1.44 - 1.26	488	488	100.0	17.82	30.59	60.26	0.0357	0.0115
1.26 - 1.14	512	512	100.0	17.20	28.00	53.85	0.0407	0.0129
1.14 - 1.06	484	484	100.0	14.52	21.09	39.73	0.0498	0.0171
1.06 - 0.99	564	564	100.0	11.22	19.30	32.22	0.0539	0.0214
0.99 - 0.94	503	503	100.0	9.55	15.82	25.81	0.0642	0.0269
0.94 - 0.90	492	492	100.0	8.42	13.88	21.57	0.0709	0.0326
0.90 - 0.87	440	440	100.0	7.95	11.11	18.44	0.0805	0.0396
0.87 - 0.84	478	478	100.0	7.62	10.93	17.52	0.0862	0.0422
0.84 - 0.81	571	571	100.0	7.28	11.09	16.40	0.0887	0.0450
0.81 - 0.79	435	435	100.0	7.03	10.11	15.00	0.0948	0.0504
0.79 - 0.76	723	723	100.0	6.87	8.39	12.53	0.1148	0.0604
0.76 - 0.75	283	283	100.0	6.52	6.93	10.29	0.1370	0.0739
0.75 - 0.73	587	587	100.0	6.42	7.32	10.43	0.1353	0.0731
0.73 - 0.71	626	626	100.0	6.30	6.11	8.73	0.1565	0.0882
0.71 - 0.70	362	362	100.0	5.91	6.33	8.75	0.1560	0.0919
0.70 - 0.69	380	380	100.0	5.95	5.03	7.18	0.1995	0.1128
0.69 - 0.67	816	816	100.0	5.34	4.96	6.59	0.2050	0.1259
0.67 - 0.66	182	255	71.4	1.83	4.02	3.77	0.2148	0.2344
0.76 - 0.66	3236	3309	97.8	5.68	5.89	8.18	0.1636	0.0999

*Table S16: Crystal data and structure refinement of **7**.*

Identification code	15393	
Empirical formula	$C_{33} H_{23} I_2 N_2 Ni$	
Color	orange	
Formula weight	760.04 g · mol^{-1}	
Temperature	100(2) K	
Wavelength	0.71073 Å	
Crystal system	MONOCLINIC	
Space group	**$P2_1/c$, (no. 14)**	
Unit cell dimensions	a = 11.8061(6) Å	α= 90°.
	b = 25.6066(13) Å	β= 99.787(2)°.
	c = 9.2124(5) Å	γ = 90°.
Volume	2744.5(2) Å3	
Z	4	
Density (calculated)	1.839 Mg · m^{-3}	
Absorption coefficient	2.982 mm-1	
F(000)	1476 e	
Crystal size	0.117 x 0.095 x 0.05 mm3	
θ range for data collection	1.590 to 32.344°.	
Index ranges	$-17 \le h \le 17$, $-38 \le k \le 37$, $-13 \le l \le 13$	
Reflections collected	94489	
Independent reflections	9731 [R_{int} = 0.0483]	
Reflections with I>2σ(I)	7927	
Completeness to θ = 25.242°	100.0 %	
Absorption correction	Gaussian	
Max. and min. transmission	0.89 and 0.77	
Refinement method	Full-matrix least-squares on F^2	
Data / restraints / parameters	9731 / 0 / 343	
Goodness-of-fit on F2	1.024	
Final R indices [I>2σ(I)]	R_1 = 0.0247	wR^2 = 0.0456
R indices (all data)	R_1 = 0.0393	wR^2 = 0.0496
Largest diff. peak and hole	0.6 and -0.6 e · Å^{-3}	

*Table S17: Bond lengths [Å] and angles [°] of **7**.*

I(1)-Ni(1)	2.5996(3)	I(2)-Ni(1)	2.5029(3)
Ni(1)-N(1)	2.0039(15)	Ni(1)-N(2)	1.9768(15)
N(1)-C(1)	1.340(2)	N(1)-C(12)	1.361(2)
N(2)-C(10)	1.328(2)	N(2)-C(11)	1.359(2)
C(1)-C(2)	1.404(2)	C(1)-C(13)	1.478(2)
C(2)-C(3)	1.380(3)	C(3)-C(4)	1.423(2)
C(3)-C(19)	1.475(2)	C(4)-C(5)	1.432(3)
C(4)-C(12)	1.401(2)	C(5)-C(6)	1.359(3)
C(6)-C(7)	1.432(2)	C(7)-C(8)	1.424(2)
C(7)-C(11)	1.401(2)	C(8)-C(9)	1.382(3)
C(8)-C(25)	1.478(3)	C(9)-C(10)	1.392(3)
C(11)-C(12)	1.436(2)	C(13)-C(14)	1.393(3)
C(13)-C(18)	1.398(3)	C(14)-C(15)	1.381(3)
C(15)-C(16)	1.385(3)	C(16)-C(17)	1.383(3)
C(17)-C(18)	1.385(3)	C(19)-C(20)	1.401(3)
C(19)-C(24)	1.391(3)	C(20)-C(21)	1.385(3)
C(21)-C(22)	1.386(3)	C(22)-C(23)	1.385(3)
C(23)-C(24)	1.387(3)	C(25)-C(26)	1.394(3)
C(25)-C(30)	1.393(3)	C(26)-C(27)	1.386(3)
C(27)-C(28)	1.382(3)	C(28)-C(29)	1.380(3)
C(29)-C(30)	1.387(3)	C(91)-C(92)	1.388(3)
C(91)-C(93)*	1.385(3)	C(92)-C(93)	1.386(3)
I(2)-Ni(1)-I(1)	113.393(10)	N(1)-Ni(1)-I(1)	97.61(4)
N(1)-Ni(1)-I(2)	143.13(4)	N(2)-Ni(1)-I(1)	95.87(5)
N(2)-Ni(1)-I(2)	111.74(4)	N(2)-Ni(1)-N(1)	82.97(6)
C(1)-N(1)-Ni(1)	130.54(13)	C(1)-N(1)-C(12)	118.58(15)
C(12)-N(1)-Ni(1)	110.79(11)	C(10)-N(2)-Ni(1)	129.14(13)
C(10)-N(2)-C(11)	118.45(16)	C(11)-N(2)-Ni(1)	112.05(12)
N(1)-C(1)-C(2)	120.61(16)	N(1)-C(1)-C(13)	117.58(16)
C(2)-C(1)-C(13)	121.71(16)	C(3)-C(2)-C(1)	121.77(17)
C(2)-C(3)-C(4)	117.46(16)	C(2)-C(3)-C(19)	120.36(16)
C(4)-C(3)-C(19)	122.17(16)	C(3)-C(4)-C(5)	124.15(16)
C(12)-C(4)-C(3)	117.48(16)	C(12)-C(4)-C(5)	118.21(16)

C(6)-C(5)-C(4)	121.64(17)	C(5)-C(6)-C(7)	121.26(17)
C(8)-C(7)-C(6)	124.44(17)	C(11)-C(7)-C(6)	117.99(16)
C(11)-C(7)-C(8)	117.56(16)	C(7)-C(8)-C(25)	122.24(16)
C(9)-C(8)-C(7)	117.84(17)	C(9)-C(8)-C(25)	119.92(16)
C(8)-C(9)-C(10)	120.68(17)	N(2)-C(10)-C(9)	122.25(17)
N(2)-C(11)-C(7)	123.15(16)	N(2)-C(11)-C(12)	116.06(15)
C(7)-C(11)-C(12)	120.77(16)	N(1)-C(12)-C(4)	123.57(16)
N(1)-C(12)-C(11)	116.36(15)	C(4)-C(12)-C(11)	120.04(16)
C(14)-C(13)-C(1)	121.96(16)	C(14)-C(13)-C(18)	118.68(17)
C(18)-C(13)-C(1)	119.08(17)	C(15)-C(14)-C(13)	120.76(17)
C(14)-C(15)-C(16)	120.07(18)	C(17)-C(16)-C(15)	119.82(18)
C(16)-C(17)-C(18)	120.34(18)	C(17)-C(18)-C(13)	120.22(18)
C(20)-C(19)-C(3)	120.53(17)	C(24)-C(19)-C(3)	120.30(17)
C(24)-C(19)-C(20)	119.08(17)	C(21)-C(20)-C(19)	120.19(18)
C(20)-C(21)-C(22)	120.16(19)	C(23)-C(22)-C(21)	120.02(18)
C(22)-C(23)-C(24)	120.05(19)	C(23)-C(24)-C(19)	120.48(18)
C(26)-C(25)-C(8)	121.08(17)	C(30)-C(25)-C(8)	119.71(17)
C(30)-C(25)-C(26)	119.19(18)	C(27)-C(26)-C(25)	120.16(18)
C(28)-C(27)-C(26)	120.11(19)	C(29)-C(28)-C(27)	120.24(19)
C(28)-C(29)-C(30)	119.98(19)	C(29)-C(30)-C(25)	120.29(18)
C(93)*-C(91)-C(92)	119.60(19)	C(93)-C(92)-C(91)	120.0(2)
C(91)*-C(93)-C(92)	120.38(19)		

Symmetry transformations used to generate equivalent atoms: * -x+2,-y+1,-z+1

Single Crystal structure of 8

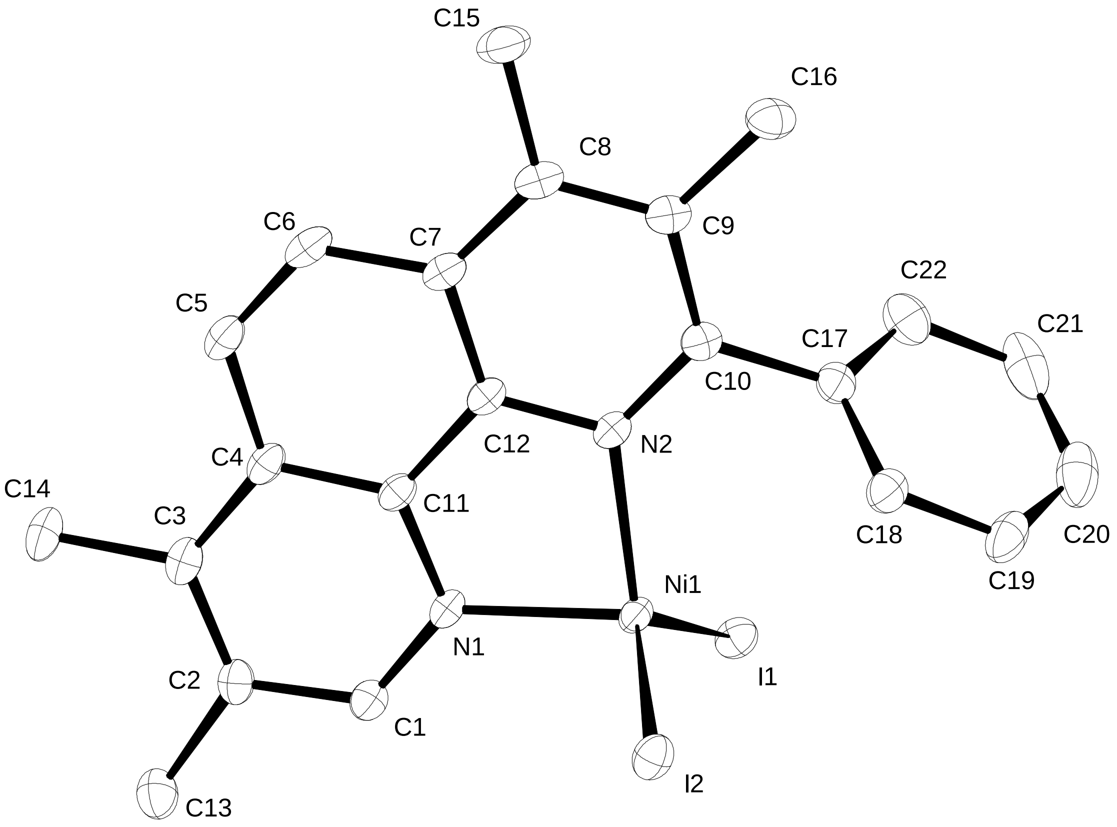

*Figure S58: The molecular structure of **8**; H atoms have been removed for clarity.*

X-Ray Crystal Structure Analysis of 8:

$C_{22}H_{20}I_2N_2Ni$, M_r = 624.91 g mol^{-1}, orange plate, crystal size 0.091 x 0.051 x 0.043 mm^3, Monoclinic, space group *P2₁/n* [14], *a* = 10.7502(3) Å, b = 17.6109(5) Å, *c* = 11.9528(3) Å, β = 110.7140(10)°, *V* = 2116.63(10) Å^3, *T* = 100(2) K, *Z* = 4, D_{calc} = 1.961 g·cm^3, λ = 0.71073 Å, $\mu(Mo\text{-}K_\alpha)$ = 3.843 mm^{-1}, Gaussian absorption correction (T_{min} = 0.79, T_{max} = 0.90), Bruker-AXS D8 VENTURE with APEX-III detector and IµS microfocus Mo-anode X-ray source, 2.158 < θ < 31.554°, 215286 measured reflections, 7081 independent reflections, 6296 reflections with $I > 2\sigma(I)$, R_{int} = 0.0793. The structure was solved by *SHELXT* and refined by full-matrix least-squares (*SHELXL*) against F^2 to R_1 = 0.0220 [$I > 2\sigma(I)$], wR_2 = 0.0507 [all data], 248 parameters and 0 restraints.

Complete .cif data for the compound are available under the CSD number 2455392.

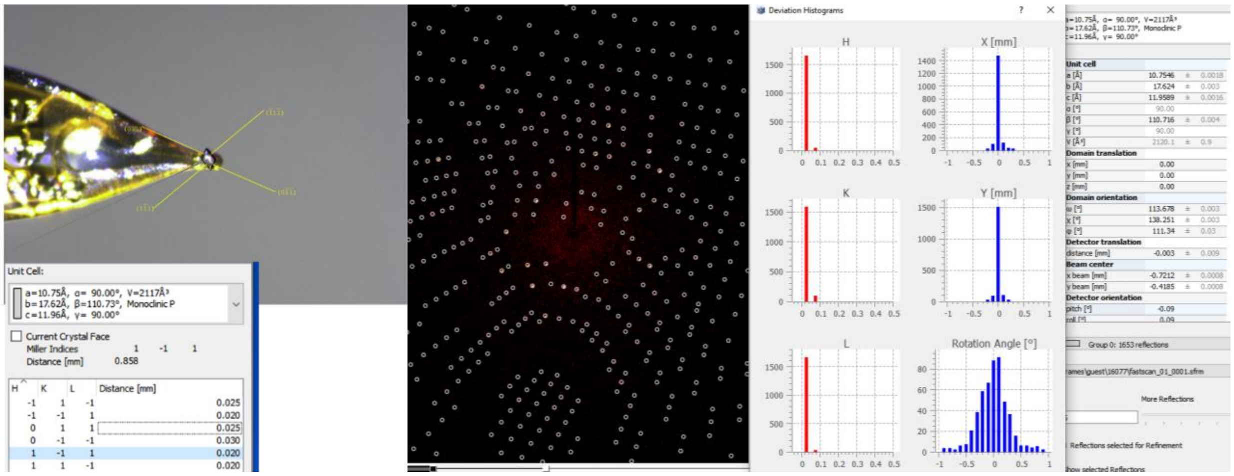

*Figure S59: Crystal faces of **8**.*

*Table S18: Intensity statistics for dataset of the X-ray crystal structure analysis of **8**.*

Resolution	#Data	#Theory	%Compl.	Redund.	Mean I	Mean I/s	Rmerge	Rsigma
2.82 - 1.87	254	254	100.0	41.46	85.52	86.56	0.0339	0.0083
1.87 - 1.48	365	365	100.0	41.31	58.05	68.55	0.0438	0.0095
1.48 - 1.28	393	393	100.0	40.53	40.11	59.37	0.0594	0.0115
1.28 - 1.17	335	335	100.0	39.44	36.75	52.68	0.0658	0.0128
1.17 - 1.08	374	374	100.0	39.60	29.57	45.80	0.0753	0.0143
1.08 - 1.02	358	358	100.0	36.40	25.35	38.77	0.0825	0.0165
1.02 - 0.96	437	437	100.0	32.44	20.22	33.86	0.0990	0.0204
0.96 - 0.92	331	331	100.0	31.42	15.18	27.22	0.1184	0.0252
0.92 - 0.88	426	426	100.0	28.85	16.02	26.16	0.1171	0.0259
0.88 - 0.85	361	361	100.0	28.65	11.87	21.45	0.1386	0.0310
0.85 - 0.83	279	279	100.0	28.04	12.85	21.95	0.1395	0.0310
0.83 - 0.80	458	458	100.0	27.26	11.58	19.37	0.1472	0.0336
0.80 - 0.78	353	353	100.0	26.44	8.67	16.32	0.1708	0.0414
0.78 - 0.76	378	378	100.0	26.00	9.41	16.44	0.1706	0.0410
0.76 - 0.74	445	445	100.0	25.08	8.77	15.35	0.1822	0.0446
0.74 - 0.73	230	230	100.0	23.29	6.46	12.11	0.2172	0.0570
0.73 - 0.71	489	489	100.0	22.25	6.41	11.71	0.2239	0.0600
0.71 - 0.70	296	296	100.0	20.77	5.99	10.10	0.2331	0.0672
0.70 - 0.69	291	291	100.0	20.73	5.36	9.41	0.2503	0.0719
0.69 - 0.68	334	335	99.7	18.73	5.10	8.48	0.2519	0.0959
0.78 - 0.68	2463	2464	100.0	22.60	6.95	12.23	0.2063	0.0577

*Table S19: Crystal data and structure refinement of **8**.*

Identification code	16077	
Empirical formula	$C_{22} H_{20} I_2 N_2 Ni$	
Color	orange	
Formula weight	624.91 g · mol^{-1}	
Temperature	100(2) K	
Wavelength	0.71073 Å	
Crystal system	MONOCLINIC	
Space group	**$P2_1/n$, (no. 14)**	
Unit cell dimensions	a = 10.7502(3) Å	α= 90°.
	b = 17.6109(5) Å	β= 110.7140(10)°.
	c = 11.9528(3) Å	γ = 90°.
Volume	2116.63(10) $Å^3$	
Z	4	
Density (calculated)	1.961 Mg · m^{-3}	
Absorption coefficient	3.843 mm-1	
F(000)	1200 e	
Crystal size	0.091 x 0.051 x 0.043 mm3	
θ range for data collection	2.158 to 31.554°.	
Index ranges	$-15 \le h \le 15$, $-25 \le k \le 25$, $-17 \le l \le 17$	
Reflections collected	215286	
Independent reflections	7081 [R_{int} = 0.0793]	
Reflections with $I>2\sigma(I)$	6296	
Completeness to θ = 25.242°	100.0 %	
Absorption correction	Gaussian	
Max. and min. transmission	0.90 and 0.79	
Refinement method	Full-matrix least-squares on F^2	
Data / restraints / parameters	7081 / 0 / 248	
Goodness-of-fit on F2	1.090	
Final R indices [$I>2\sigma(I)$]	R_1 = 0.0220	wR^2 = 0.0486
R indices (all data)	R_1 = 0.0270	wR^2 = 0.0507
Largest diff. peak and hole	0.7and -0.6 e · $Å^{-3}$	

*Table S20: Bond lengths [Å] and angles [°] of **8**.*

I(1)-Ni(1)	2.5320(3)	I(2)-Ni(1)	2.5663(3)
Ni(1)-N(1)	1.9826(16)	Ni(1)-N(2)	2.0007(16)
N(1)-C(1)	1.324(2)	N(1)-C(11)	1.357(2)
N(2)-C(10)	1.336(2)	N(2)-C(12)	1.360(2)
C(1)-C(2)	1.408(3)	C(2)-C(3)	1.395(3)
C(2)-C(13)	1.496(3)	C(3)-C(4)	1.418(3)
C(3)-C(14)	1.505(3)	C(4)-C(5)	1.436(3)
C(4)-C(11)	1.405(3)	C(5)-C(6)	1.358(3)
C(6)-C(7)	1.440(3)	C(7)-C(8)	1.416(3)
C(7)-C(12)	1.407(3)	C(8)-C(9)	1.393(3)
C(8)-C(15)	1.507(3)	C(9)-C(10)	1.416(3)
C(9)-C(16)	1.507(3)	C(10)-C(17)	1.490(3)
C(11)-C(12)	1.432(3)	C(17)-C(18)	1.390(3)
C(17)-C(22)	1.395(3)	C(18)-C(19)	1.390(3)
C(19)-C(20)	1.384(4)	C(20)-C(21)	1.390(4)
C(21)-C(22)	1.381(3)		
I(1)-Ni(1)-I(2)	131.005(11)	N(1)-Ni(1)-I(1)	107.31(5)
N(1)-Ni(1)-I(2)	100.96(5)	N(1)-Ni(1)-N(2)	82.94(7)
N(2)-Ni(1)-I(1)	111.03(5)	N(2)-Ni(1)-I(2)	111.50(4)
C(1)-N(1)-Ni(1)	129.01(13)	C(1)-N(1)-C(11)	118.54(16)
C(11)-N(1)-Ni(1)	112.44(13)	C(10)-N(2)-Ni(1)	128.94(13)
C(10)-N(2)-C(12)	119.28(16)	C(12)-N(2)-Ni(1)	111.75(13)
N(1)-C(1)-C(2)	123.65(18)	C(1)-C(2)-C(13)	118.40(18)
C(3)-C(2)-C(1)	118.33(18)	C(3)-C(2)-C(13)	123.26(18)
C(2)-C(3)-C(4)	118.72(17)	C(2)-C(3)-C(14)	120.54(19)
C(4)-C(3)-C(14)	120.73(18)	C(3)-C(4)-C(5)	124.65(18)
C(11)-C(4)-C(3)	118.17(17)	C(11)-C(4)-C(5)	117.17(18)
C(6)-C(5)-C(4)	121.82(18)	C(5)-C(6)-C(7)	121.96(18)
C(8)-C(7)-C(6)	125.53(18)	C(12)-C(7)-C(6)	117.01(18)
C(12)-C(7)-C(8)	117.44(18)	C(7)-C(8)-C(15)	121.24(19)
C(9)-C(8)-C(7)	119.49(18)	C(9)-C(8)-C(15)	119.23(19)
C(8)-C(9)-C(10)	118.92(18)	C(8)-C(9)-C(16)	120.26(18)
C(10)-C(9)-C(16)	120.81(18)	N(2)-C(10)-C(9)	121.90(18)
N(2)-C(10)-C(17)	114.67(16)	C(9)-C(10)-C(17)	123.25(18)
N(1)-C(11)-C(4)	122.50(18)	N(1)-C(11)-C(12)	116.40(16)
C(4)-C(11)-C(12)	121.01(17)	N(2)-C(12)-C(7)	122.75(18)
N(2)-C(12)-C(11)	116.27(16)	C(7)-C(12)-C(11)	120.87(17)
C(18)-C(17)-C(10)	121.14(19)	C(18)-C(17)-C(22)	119.5(2)
C(22)-C(17)-C(10)	119.03(19)	C(19)-C(18)-C(17)	120.0(2)
C(20)-C(19)-C(18)	120.6(2)	C(19)-C(20)-C(21)	119.0(2)
C(22)-C(21)-C(20)	121.0(2)	C(21)-C(22)-C(17)	119.8(2)

Single Crystal structure of S1

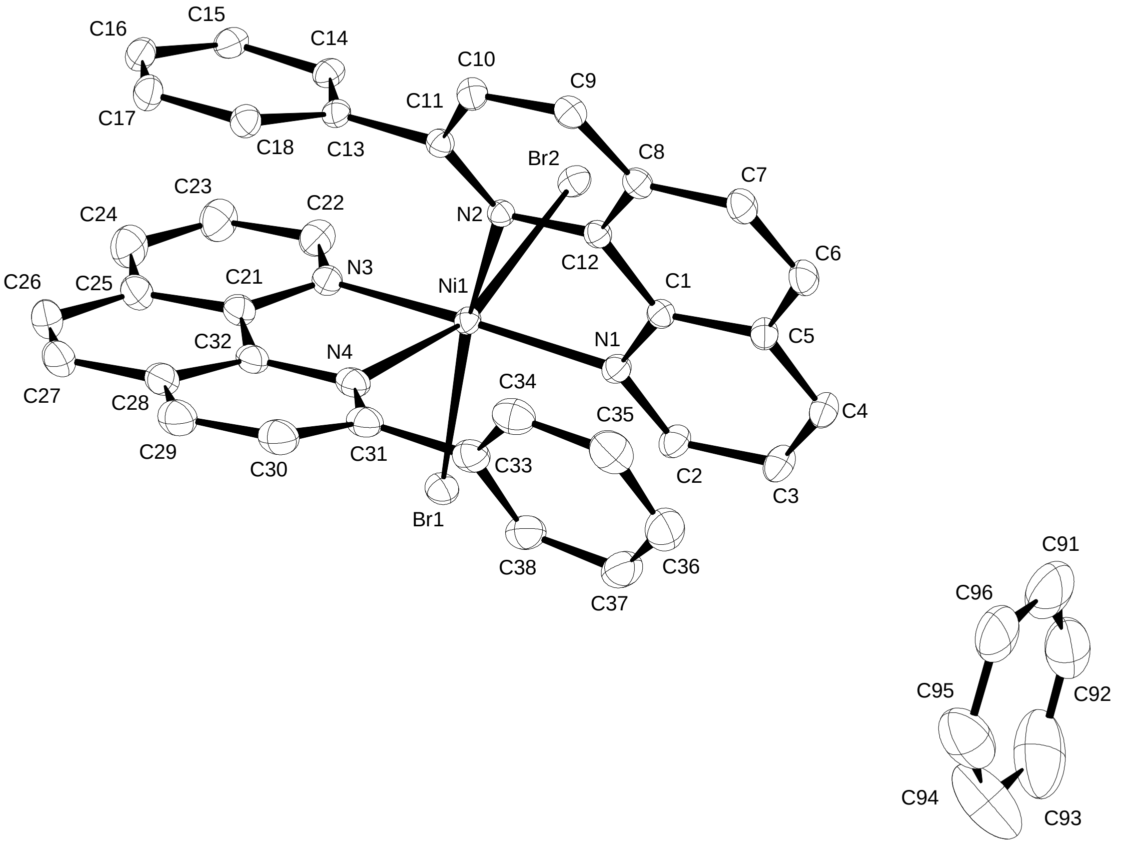

Figure S60: The molecular structure of ***S1****; H atoms have been removed for clarity.*

X-Ray Crystal Structure Analysis of S1:

$C_{42}H_{30}Br_2N_4Ni$, M_r = 809.23 g mol^{-1}, neon-green prism, crystal size 0.263 x 0.15 x 0.071 mm^3, Monoclinic, space group *P2₁/c* [14], *a* = 8.5535(5) Å, b = 15.4957(11) Å, *c* = 25.9336(18) Å, β = 95.545(3)°, *V* = 3421.2(4) $Å^3$, *T* = 100(2) K, *Z* = 4, D_{calc} = 1.571 g·cm^3, λ = 0.71073 Å, $\mu(Mo\text{-}K_\alpha)$ = 2.940 mm^{-1}, Gaussian absorption correction (T_{min} = 0.39, T_{max} = 0.67), Bruker-AXS D8 VENTURE with APEX-III detector and IµS microfocus Mo-anode X-ray source, 2.392 < θ < 36.429°, 502781 measured reflections, 16682 independent reflections, 12079 reflections with $I > 2\sigma(I)$, R_{int} = 0.0991. The structure was solved by *SHELXT* and refined by full-matrix least-squares (*SHELXL*) against F^2 to R_1 = 0.0398 [$I > 2\sigma(I)$], wR_2 = 0.0910 [all data], 442 parameters and 0 restraints.

Complete .cif data for the compound are available under the CSD number 2455393.

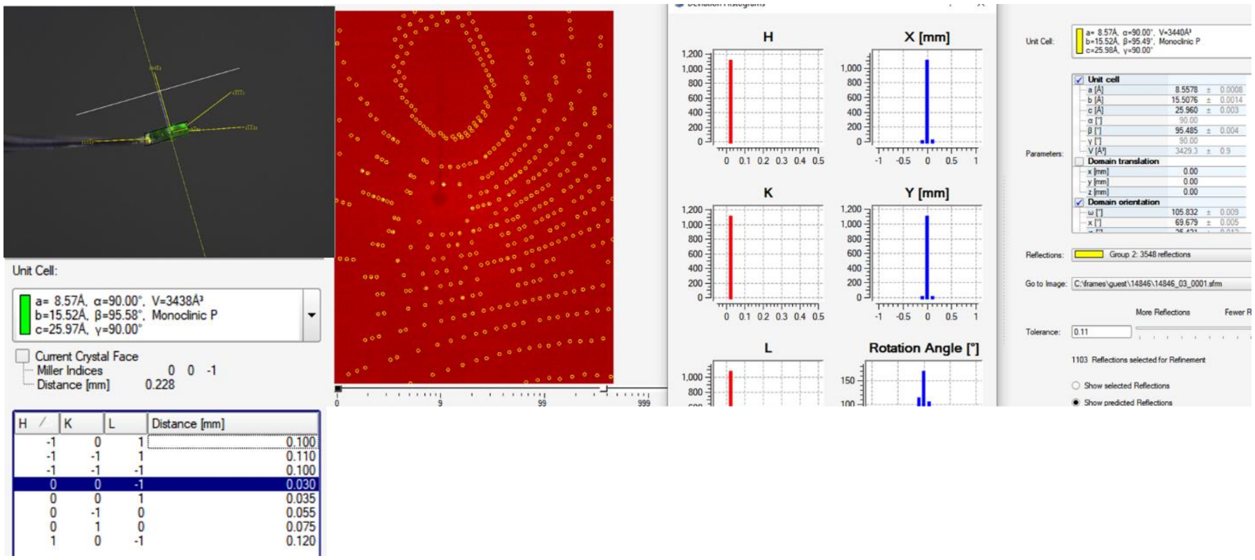

*Figure S61: Unit cell determination/refinement of **S1**.*

*Table S21: Intensity statistics for dataset of the X-ray crystal structure analysis of **S1**.*

Resolution	#Data	#Theory	%Compl.	Redund.	Mean I	Mean I/s	Rmerge	Rsigma
Inf - 2.49	258	263	98.1	51.29	62.96	91.34	0.0279	0.0089
2.49 - 1.65	602	602	100.0	56.14	37.19	85.43	0.0344	0.0081
1.65 - 1.30	882	882	100.0	55.33	21.65	66.18	0.0499	0.0098
1.30 - 1.13	891	891	100.0	53.18	18.11	56.91	0.0636	0.0115
1.13 - 1.03	815	815	100.0	42.65	12.95	42.74	0.0776	0.0151
1.03 - 0.95	913	913	100.0	38.27	9.87	34.68	0.0973	0.0196
0.95 - 0.89	946	946	100.0	34.56	8.02	27.96	0.1161	0.0247
0.89 - 0.85	758	758	100.0	33.21	7.17	24.84	0.1364	0.0293
0.85 - 0.81	949	949	100.0	30.37	5.92	19.78	0.1612	0.0368
0.81 - 0.78	812	812	100.0	30.35	5.33	17.48	0.1807	0.0426
0.78 - 0.75	986	986	100.0	28.30	4.12	13.71	0.2237	0.0574
0.75 - 0.73	738	738	100.0	26.03	3.78	11.74	0.2505	0.0685
0.73 - 0.71	797	797	100.0	25.47	3.18	9.61	0.2929	0.0850
0.71 - 0.69	953	953	100.0	24.87	3.13	8.92	0.3142	0.0940
0.69 - 0.67	1000	1000	100.0	23.37	2.63	7.00	0.3754	0.1242
0.67 - 0.65	1159	1159	100.0	20.99	2.24	5.24	0.4469	0.1693
0.65 - 0.64	642	642	100.0	21.03	2.20	4.85	0.4847	0.1830
0.64 - 0.63	674	674	100.0	19.99	1.86	3.82	0.5484	0.2354
0.63 - 0.62	710	710	100.0	13.41	1.74	2.77	0.5927	0.3410
0.62 - 0.60	1689	1731	97.6	6.52	1.47	1.50	0.6829	0.7272
0.70 - 0.60	6369	6411	99.3	16.80	2.05	4.31	0.4599	0.2828

*Table S22: Crystal data and structure refinement of **S1**.*

Identification code	14846	
Empirical formula	$C_{42} H_{30} Br_2 N_4 Ni$	
Color	neon-green	
Formula weight	809.23 g · mol^{-1}	
Temperature	100(2) K	
Wavelength	0.71073 Å	
Crystal system	MONOCLINIC	
Space group	**$P2_1/c$, (no. 14)**	
Unit cell dimensions	a = 8.5535(5) Å	α= 90°.
	b = 15.4957(11) Å	β= 95.545(3)°.
	c = 25.9336(18) Å	γ = 90°.
Volume	3421.2(4) Å^3	
Z	4	
Density (calculated)	1.571 Mg · m^{-3}	
Absorption coefficient	2.940 mm^{-1}	
F(000)	1632 e	
Crystal size	0.263 x 0.15 x 0.071 mm^3	
θ range for data collection	2.392 to 36.429°.	
Index ranges	$-14 \leq h \leq 14$, $-25 \leq k \leq 25$, $-43 \leq l \leq 43$	
Reflections collected	502781	
Independent reflections	16682 [R_{int} = 0.0991]	
Reflections with $I>2\sigma(I)$	12079	
Completeness to θ = 25.242°	100.0 %	
Absorption correction	Semi-empirical from equivalents	
Max. and min. transmission	0.67 and 0.39	
Refinement method	Full-matrix least-squares on F^2	
Data / restraints / parameters	16682 / 0 / 442	
Goodness-of-fit on F2	1.023	
Final R indices [$I>2\sigma(I)$]	R_1 = 0.0398	wR^2 = 0.0812
R indices (all data)	R_1 = 0.0754	wR^2 = 0.0910
Largest diff. peak and hole	1.6 and -1.1 e · Å^{-3}	

*Table S23: Bond lengths [Å] and angles [°] of **S1**.*

Br(1)-Ni(1)	2.6255(3)	Br(2)-Ni(1)	2.6837(3)
Ni(1)-N(1)	2.0577(14)	Ni(1)-N(2)	2.1734(13)
Ni(1)-N(3)	2.0521(15)	Ni(1)-N(4)	2.2044(15)
N(1)-C(1)	1.359(2)	N(1)-C(2)	1.332(2)
N(2)-C(11)	1.341(2)	N(2)-C(12)	1.365(2)
N(3)-C(21)	1.344(2)	N(3)-C(22)	1.332(2)
N(4)-C(31)	1.333(2)	N(4)-C(32)	1.368(2)
C(1)-C(5)	1.410(2)	C(1)-C(12)	1.435(2)
C(2)-C(3)	1.401(2)	C(3)-C(4)	1.373(3)
C(4)-C(5)	1.406(2)	C(5)-C(6)	1.434(2)
C(6)-C(7)	1.357(3)	C(7)-C(8)	1.430(2)
C(8)-C(9)	1.407(2)	C(8)-C(12)	1.412(2)
C(9)-C(10)	1.366(2)	C(10)-C(11)	1.413(2)
C(11)-C(13)	1.480(2)	C(13)-C(14)	1.396(2)
C(13)-C(18)	1.401(2)	C(14)-C(15)	1.391(2)
C(15)-C(16)	1.391(3)	C(16)-C(17)	1.388(3)
C(17)-C(18)	1.391(2)	C(21)-C(25)	1.411(3)
C(21)-C(32)	1.442(3)	C(22)-C(23)	1.408(3)
C(23)-C(24)	1.362(3)	C(24)-C(25)	1.401(3)
C(25)-C(26)	1.434(3)	C(26)-C(27)	1.352(3)
C(27)-C(28)	1.436(3)	C(28)-C(29)	1.405(3)
C(28)-C(32)	1.411(2)	C(29)-C(30)	1.370(3)
C(30)-C(31)	1.412(3)	C(31)-C(33)	1.486(3)
C(33)-C(34)	1.398(3)	C(33)-C(38)	1.397(3)
C(34)-C(35)	1.388(3)	C(35)-C(36)	1.388(3)
C(36)-C(37)	1.385(3)	C(37)-C(38)	1.389(3)
C(91)-C(92)	1.350(6)	C(91)-C(96)	1.346(5)
C(92)-C(93)	1.396(6)	C(93)-C(94)	1.469(6)
C(94)-C(95)	1.346(6)	C(95)-C(96)	1.381(5)
Br(1)-Ni(1)-Br(2)	106.245(10)	N(1)-Ni(1)-Br(1)	91.07(4)
N(1)-Ni(1)-Br(2)	82.76(4)	N(1)-Ni(1)-N(2)	79.21(5)
N(1)-Ni(1)-N(4)	109.33(6)	N(2)-Ni(1)-Br(1)	164.38(4)

N(2)-Ni(1)-Br(2)	84.80(4)	N(2)-Ni(1)-N(4)	81.54(5)
N(3)-Ni(1)-Br(1)	81.36(4)	N(3)-Ni(1)-Br(2)	92.00(4)
N(3)-Ni(1)-N(1)	169.25(6)	N(3)-Ni(1)-N(2)	109.77(5)
N(3)-Ni(1)-N(4)	78.51(6)	N(4)-Ni(1)-Br(1)	90.34(4)
N(4)-Ni(1)-Br(2)	159.56(4)	C(1)-N(1)-Ni(1)	114.83(10)
C(2)-N(1)-Ni(1)	126.65(12)	C(2)-N(1)-C(1)	118.29(14)
C(11)-N(2)-Ni(1)	131.24(11)	C(11)-N(2)-C(12)	117.57(13)
C(12)-N(2)-Ni(1)	110.61(10)	C(21)-N(3)-Ni(1)	116.26(12)
C(22)-N(3)-Ni(1)	125.06(12)	C(22)-N(3)-C(21)	118.26(16)
C(31)-N(4)-Ni(1)	131.92(13)	C(31)-N(4)-C(32)	117.19(15)
C(32)-N(4)-Ni(1)	110.00(11)	N(1)-C(1)-C(5)	122.42(15)
N(1)-C(1)-C(12)	117.37(14)	C(5)-C(1)-C(12)	120.20(15)
N(1)-C(2)-C(3)	122.76(16)	C(4)-C(3)-C(2)	119.40(16)
C(3)-C(4)-C(5)	119.26(16)	C(1)-C(5)-C(6)	119.61(15)
C(4)-C(5)-C(1)	117.79(16)	C(4)-C(5)-C(6)	122.60(15)
C(7)-C(6)-C(5)	120.24(16)	C(6)-C(7)-C(8)	121.17(16)
C(9)-C(8)-C(7)	122.79(15)	C(9)-C(8)-C(12)	117.08(15)
C(12)-C(8)-C(7)	120.11(15)	C(10)-C(9)-C(8)	119.22(15)
C(9)-C(10)-C(11)	120.45(15)	N(2)-C(11)-C(10)	121.79(14)
N(2)-C(11)-C(13)	120.15(14)	C(10)-C(11)-C(13)	118.05(14)
N(2)-C(12)-C(1)	117.79(14)	N(2)-C(12)-C(8)	123.71(14)
C(8)-C(12)-C(1)	118.49(14)	C(14)-C(13)-C(11)	120.58(14)
C(14)-C(13)-C(18)	119.84(15)	C(18)-C(13)-C(11)	119.50(15)
C(15)-C(14)-C(13)	119.97(16)	C(16)-C(15)-C(14)	119.97(16)
C(17)-C(16)-C(15)	120.27(16)	C(16)-C(17)-C(18)	120.18(17)
C(17)-C(18)-C(13)	119.71(16)	N(3)-C(21)-C(25)	122.73(17)
N(3)-C(21)-C(32)	117.14(16)	C(25)-C(21)-C(32)	120.10(17)
N(3)-C(22)-C(23)	122.80(18)	C(24)-C(23)-C(22)	118.75(19)
C(23)-C(24)-C(25)	119.95(18)	C(21)-C(25)-C(26)	119.11(19)
C(24)-C(25)-C(21)	117.39(18)	C(24)-C(25)-C(26)	123.48(19)
C(27)-C(26)-C(25)	121.12(19)	C(26)-C(27)-C(28)	120.81(18)
C(29)-C(28)-C(27)	122.48(18)	C(29)-C(28)-C(32)	117.57(18)
C(32)-C(28)-C(27)	119.94(18)	C(30)-C(29)-C(28)	118.48(18)
C(29)-C(30)-C(31)	120.62(19)	N(4)-C(31)-C(30)	122.26(18)
N(4)-C(31)-C(33)	119.25(16)	C(30)-C(31)-C(33)	118.42(17)
N(4)-C(32)-C(21)	117.72(15)	N(4)-C(32)-C(28)	123.62(17)

C(28)-C(32)-C(21)	118.66(17)	C(34)-C(33)-C(31)	120.30(17)
C(38)-C(33)-C(31)	119.92(17)	C(38)-C(33)-C(34)	119.67(18)
C(35)-C(34)-C(33)	119.9(2)	C(34)-C(35)-C(36)	120.2(2)
C(37)-C(36)-C(35)	120.0(2)	C(36)-C(37)-C(38)	120.4(2)
C(37)-C(38)-C(33)	119.75(19)	C(96)-C(91)-C(92)	119.3(4)
C(91)-C(92)-C(93)	123.0(3)	C(92)-C(93)-C(94)	116.4(4)
C(95)-C(94)-C(93)	117.9(4)	C(94)-C(95)-C(96)	122.1(4)
C(91)-C(96)-C(95)	121.1(4)		

6. NMR Spectra

A multipoint base multipoint baseline correction was conducted using the segments algorithm in MestReNova.

^{1}H NMR of 2 (500 MHz, $CDCl_3$, 25 °C)

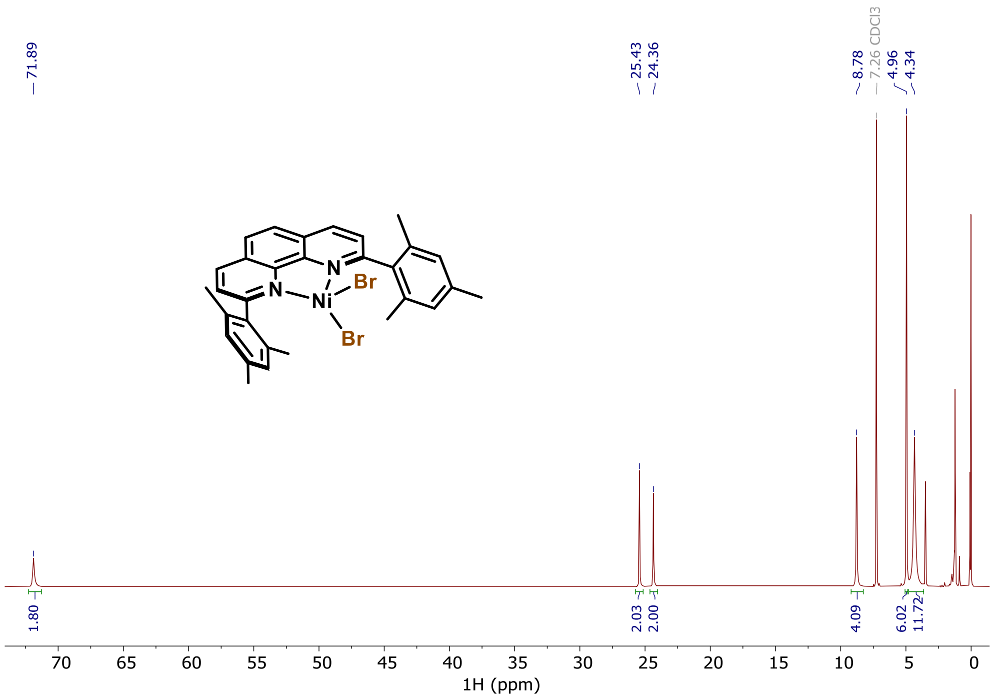

^{1}H NMR of 2 (400 MHz, THF-d_8, 25 °C)

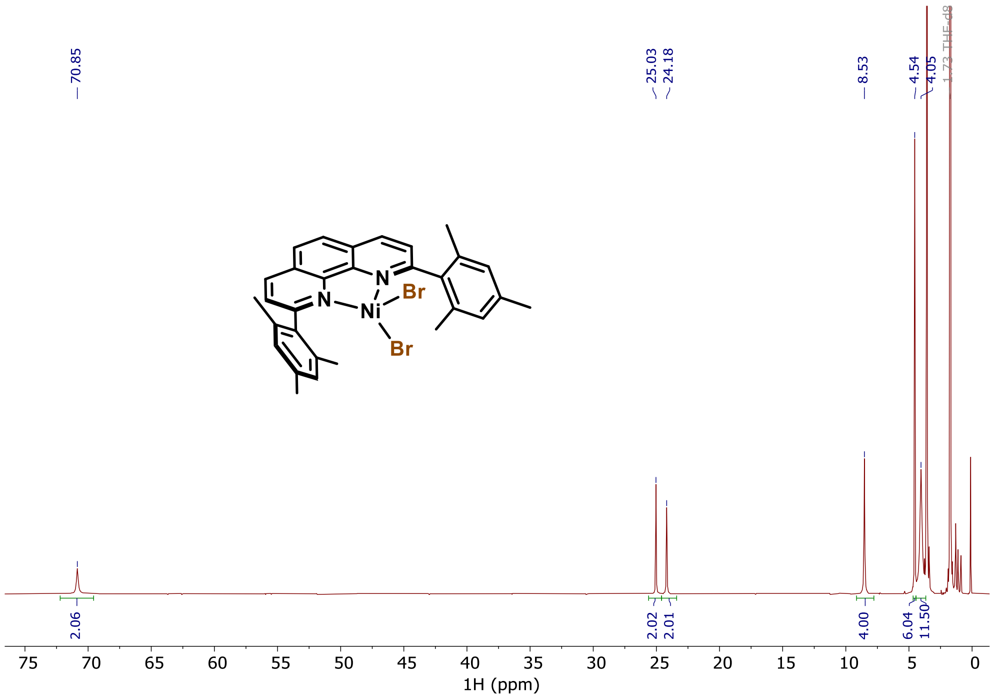

¹H NMR of 3 (500 MHz, $CDCl_3$, 25 °C)

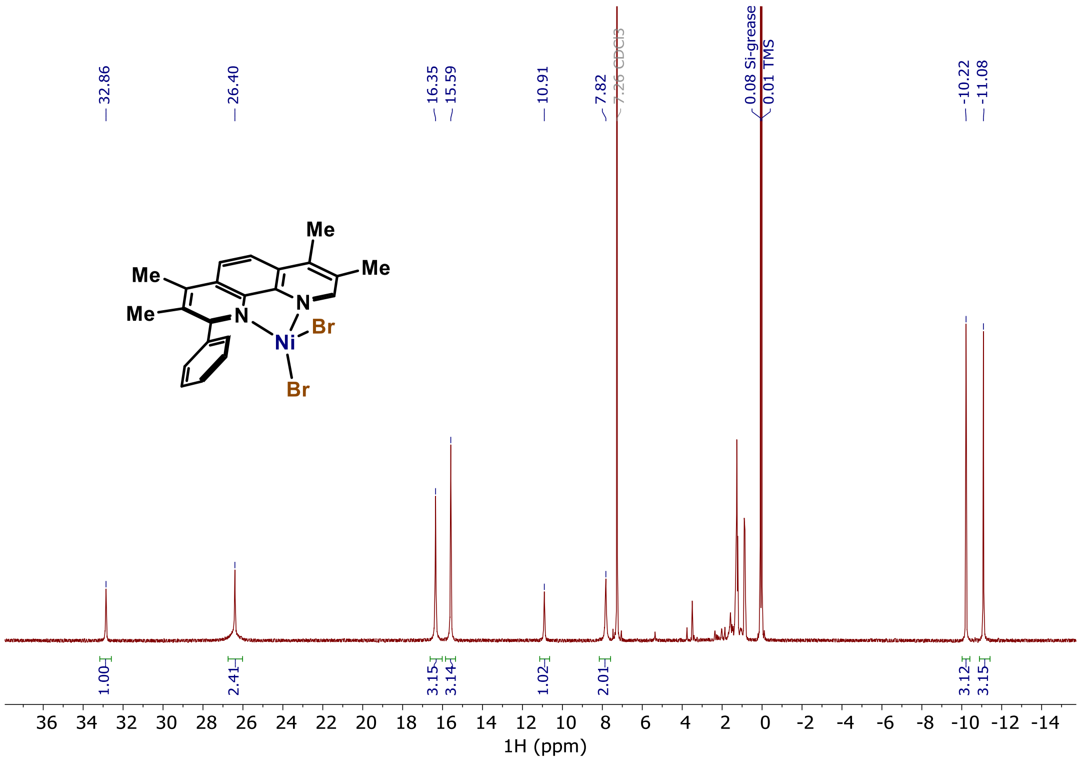

¹H NMR of 3 (400 MHz, THF-d_8, 25 °C)

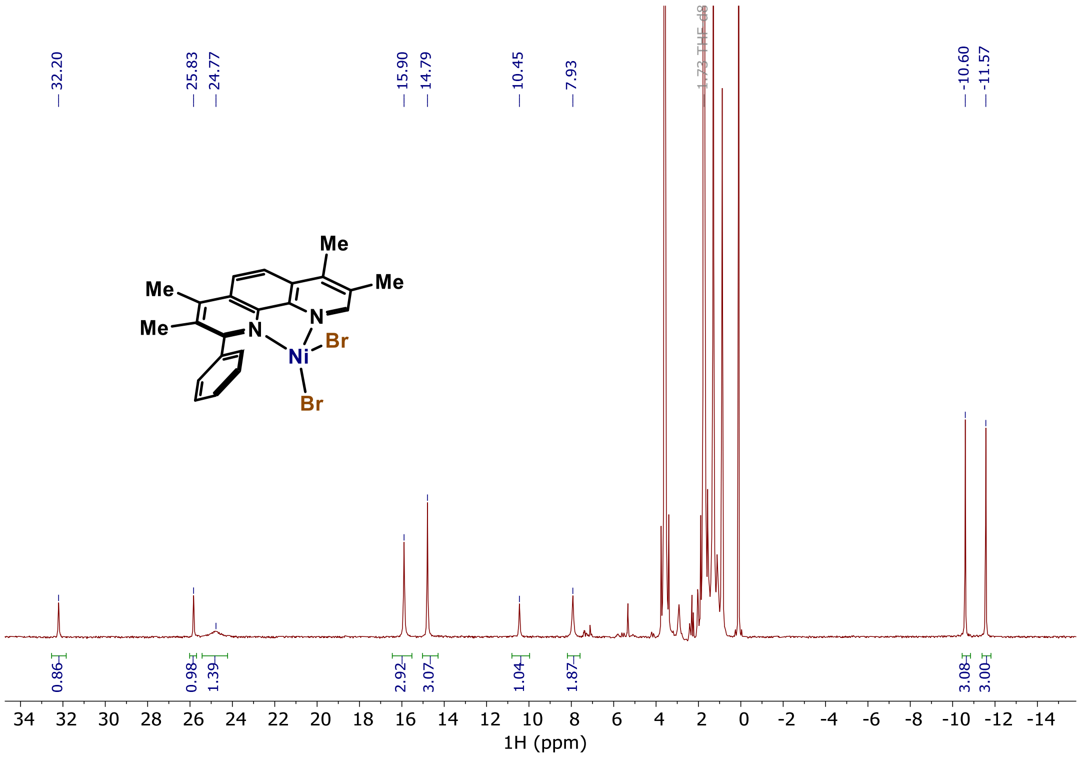

^{1}H NMR of 4 (400 MHz, $CDCl_3$, 25 °C)

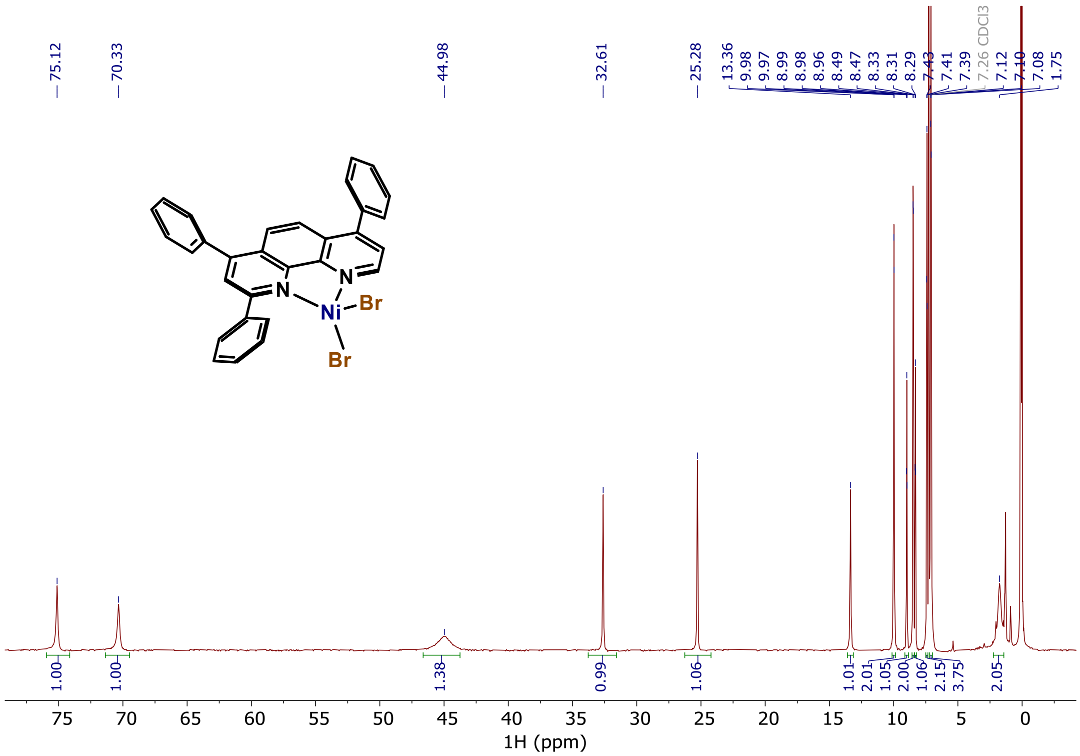

^{1}H NMR of 4 (400 MHz, THF-d_8, 25 °C)

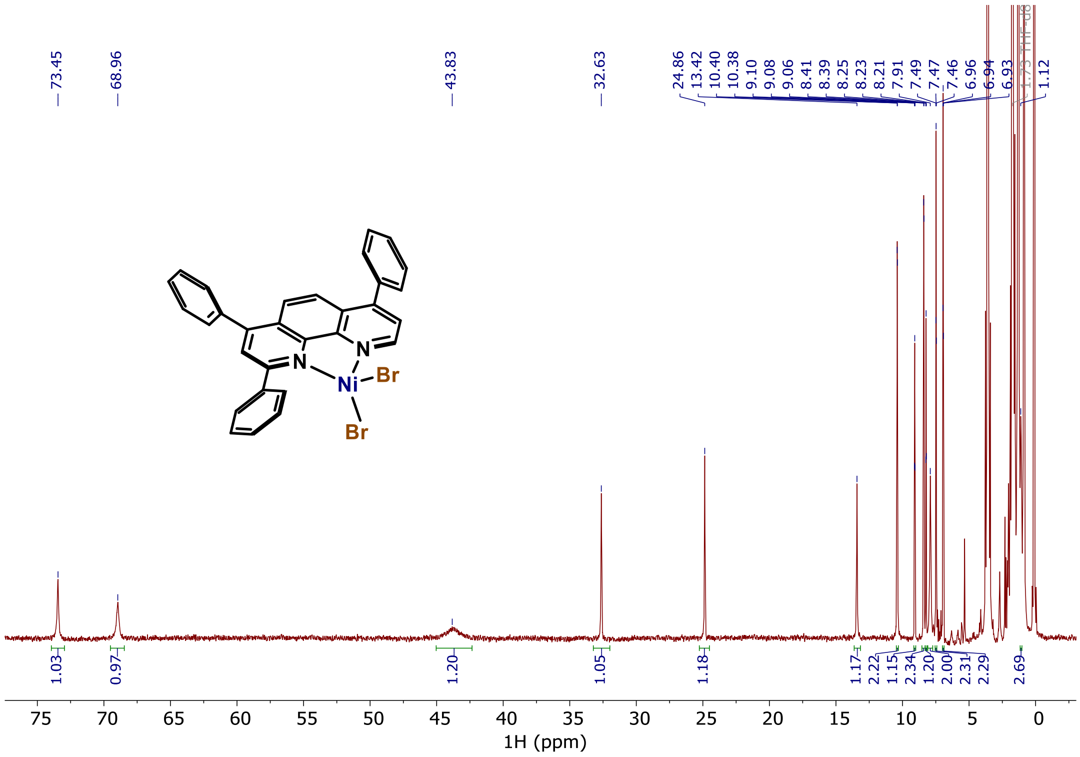

^{1}H NMR of 6 (500 MHz, $CDCl_3$, 25 °C)

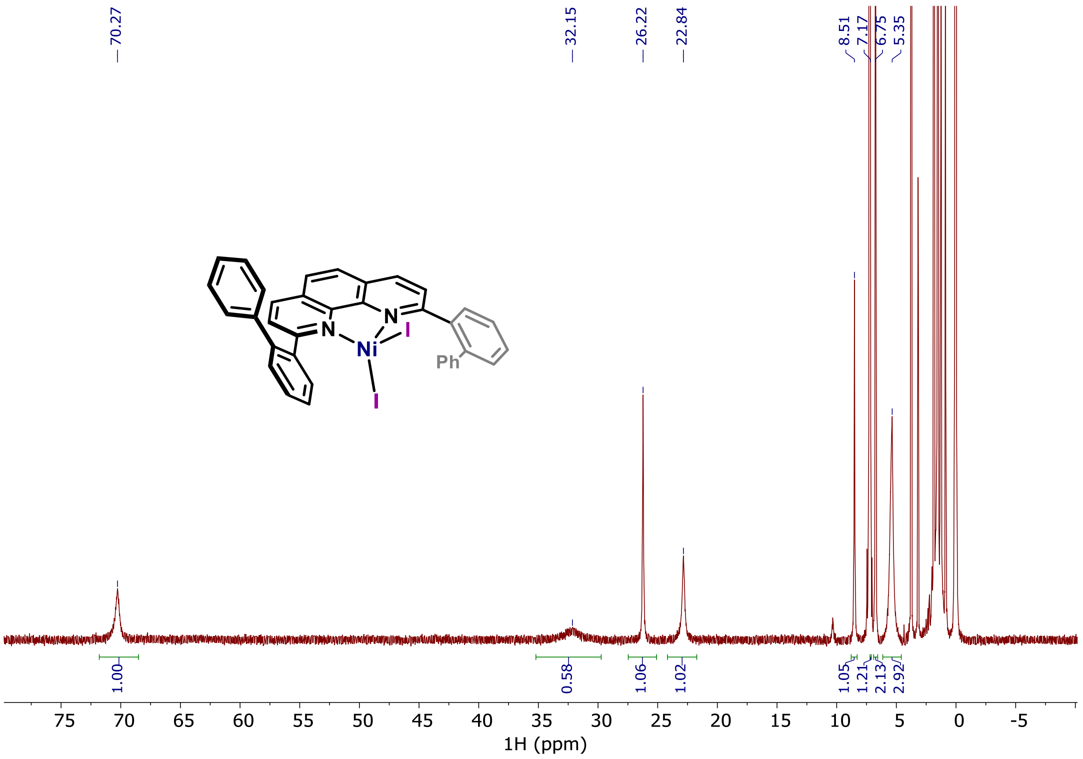

^{1}H NMR of 6 (400 MHz, THF-d_8, 25 °C)

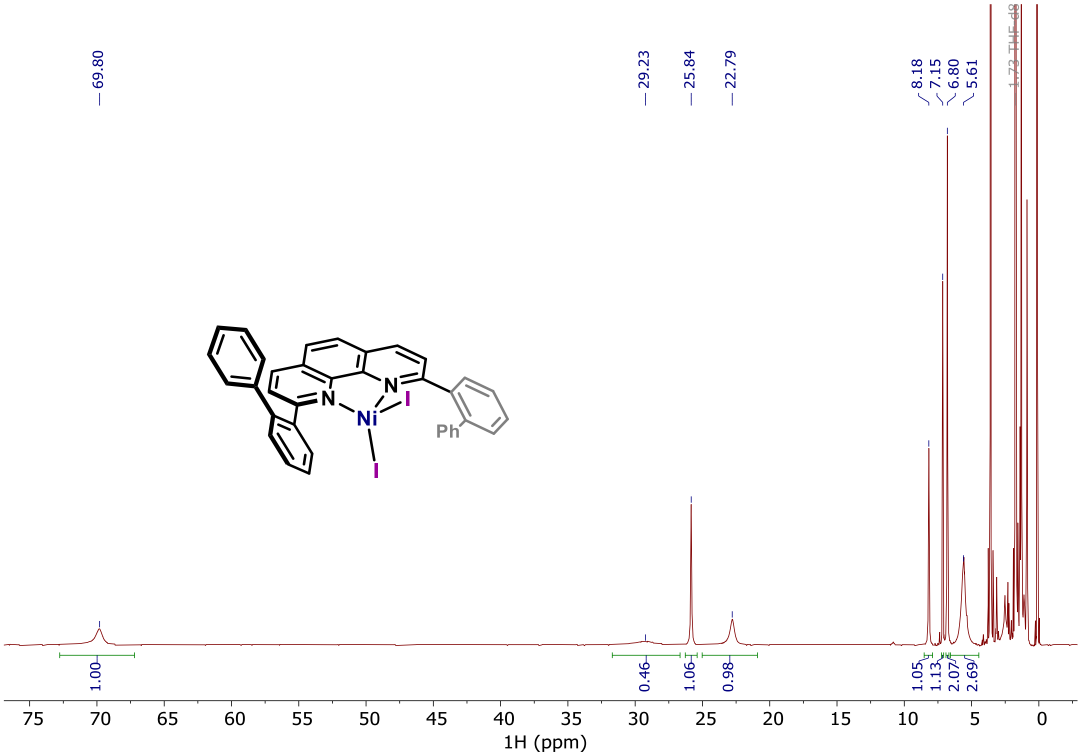

^{1}H NMR of 7 (400 MHz, $CDCl_3$, 25 °C)

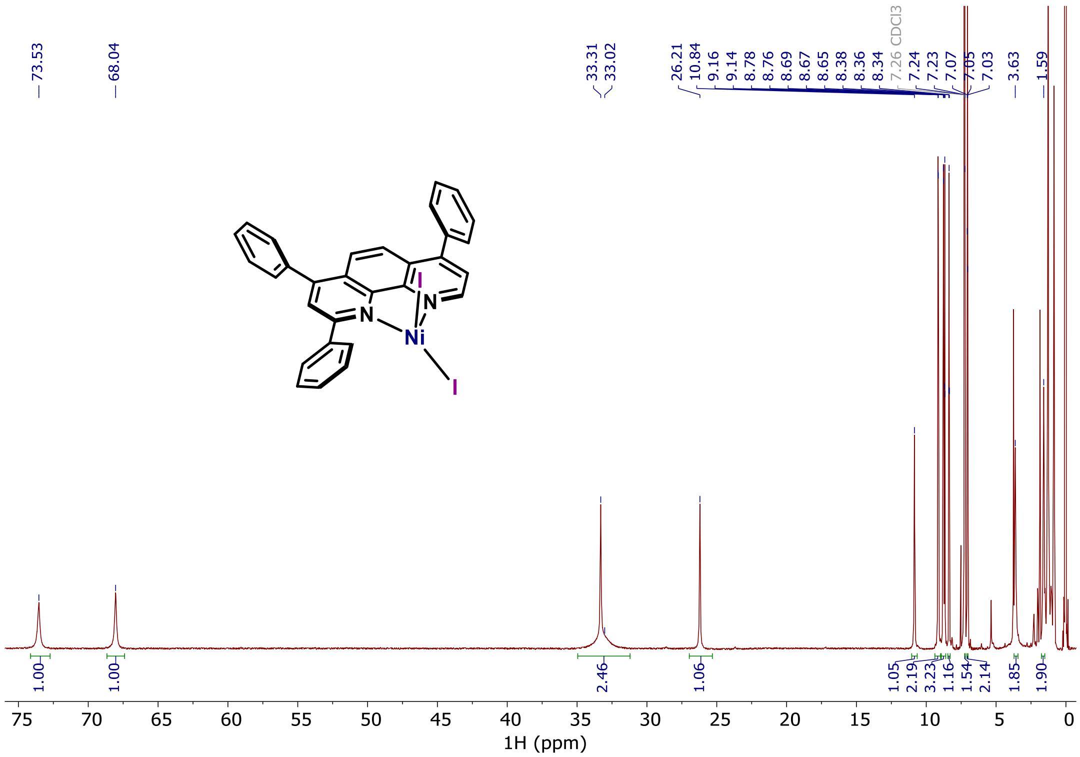

^{1}H NMR of 7 (400 MHz, THF-d_8, 25 °C)

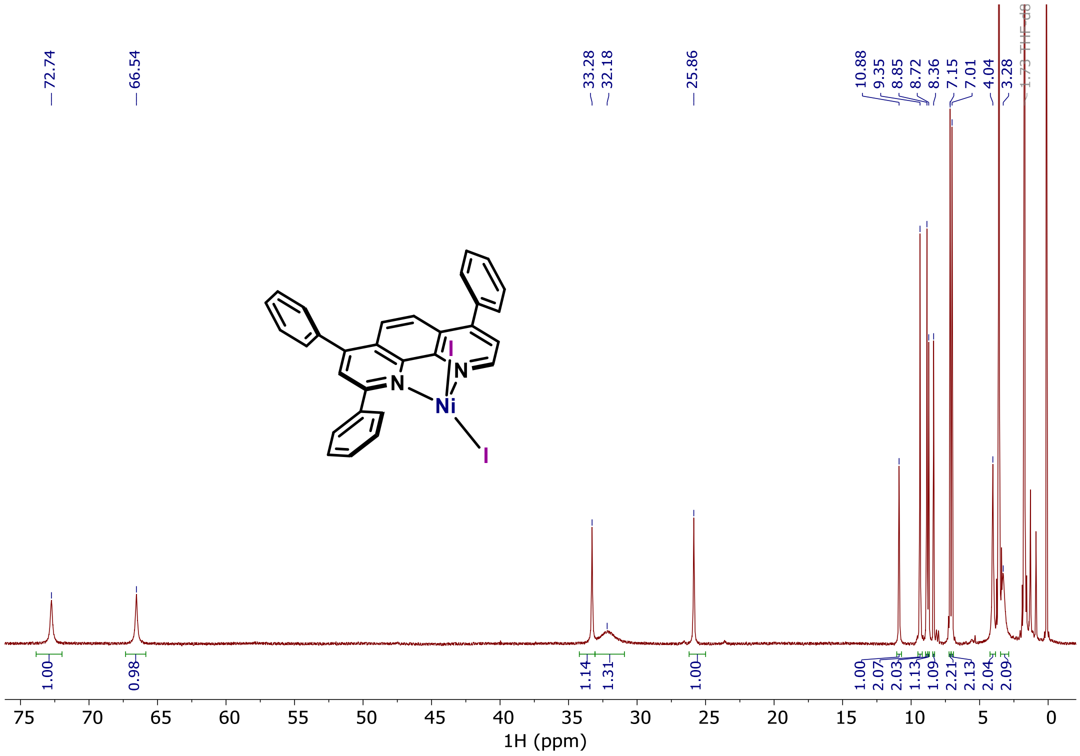

^{1}H NMR of 8 (400 MHz, $CDCl_3$, 25 °C)

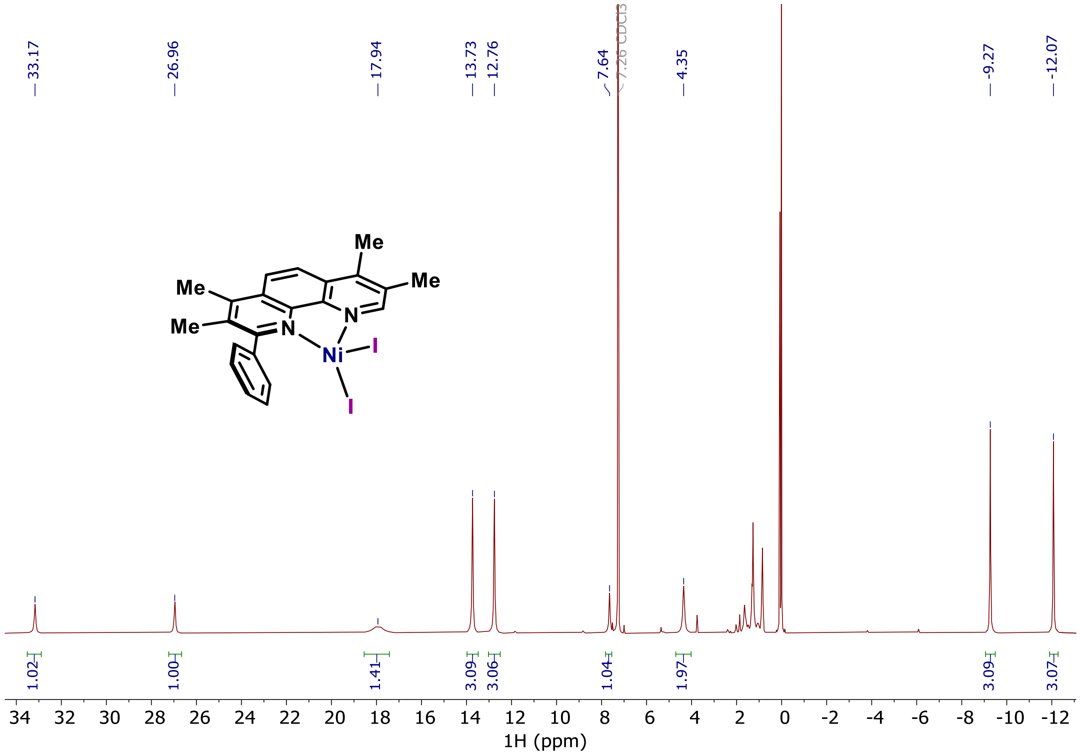

^{1}H NMR of 8 (400 MHz, THF-d_8, 25 °C)

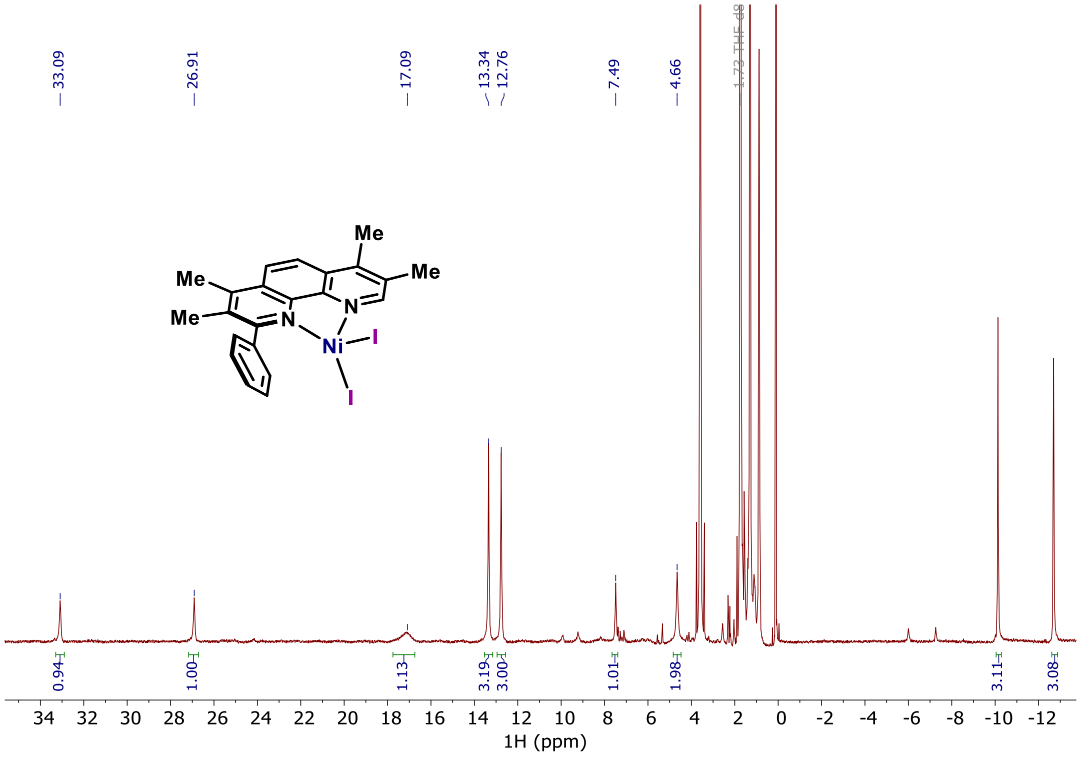

7. References

(1) Mateos-Calbet, A.; Bruzzese, P. C.; Mermigki, M. A.; Schnegg, A.; Pantazis, D. A.; Cornella, J. Rapid Oxygen Atom Transfer at a Catalysis-Relevant Ni(I)–Alkyl Complex with N_2O. *J. Am. Chem. Soc.* **2025**. DOI: 10.1021/jacs.5c03351.
(2) Somerville, R. J.; Odena, C.; Obst, M. F.; Hazari, N.; Hopmann, K. H.; Martin, R. Ni(I)–Alkyl Complexes Bearing Phenanthroline Ligands: Experimental Evidence for CO_2 Insertion at Ni(I) Centers. *J. Am. Chem. Soc.* **2020**, *142* (25), 10936-10941. DOI: 10.1021/jacs.0c04695.
(3) Vander Griend, D. A.; Bediako, D. K.; DeVries, M. J.; DeJong, N. A.; Heeringa, L. P. Detailed Spectroscopic, Thermodynamic, and Kinetic Characterization of Nickel(II) Complexes with 2,2'-Bipyridine and 1,10-Phenanthroline Attained via Equilibrium-Restricted Factor Analysis. *Inorg. Chem.* **2008**, *47* (2), 656-662. DOI: 10.1021/ic700553d.
(4) Kawamata, Y.; Vantourout, J. C.; Hickey, D. P.; Bai, P.; Chen, L.; Hou, Q.; Qiao, W.; Barman, K.; Edwards, M. A.; Garrido-Castro, A. F.; et al. Electrochemically Driven, Ni-Catalyzed Aryl Amination: Scope, Mechanism, and Applications. *J. Am. Chem. Soc.* **2019**, *141* (15), 6392-6402. DOI: 10.1021/jacs.9b01886.
(5) Chrisman, C. H.; Kudisch, M.; Puffer, K. O.; Stewart, T. K.; Lamb, Y. M. L.; Lim, C.-H.; Escobar, R.; Thordarson, P.; Johannes, J. W.; Miyake, G. M. Halide Noninnocence and Direct Photoreduction of Ni(II) Enables Coupling of Aryl Chlorides in Dual Catalytic, Carbon–Heteroatom Bond-Forming Reactions. *J. Am. Chem. Soc.* **2023**, *145* (22), 12293-12304. DOI: 10.1021/jacs.3c02784.
(6) Baldinelli, L.; Belanzoni, P.; Bistoni, G. Mechanism of Nitrous Oxide Activation in C(sp^2)–O Bond Formation Reactions Catalyzed by Nickel Complexes. *J. Am. Chem. Soc.* **2024**, *146* (9), 6016-6024. DOI: 10.1021/jacs.3c12922.
(7) Becke, A. D. Density-functional exchange-energy approximation with correct asymptotic behavior. *Phys. Rev. A* **1988**, *38* (6), 3098-3100. DOI: 10.1103/PhysRevA.38.3098.
(8) Pollak, P.; Weigend, F. Segmented Contracted Error-Consistent Basis Sets of Double- and Triple-ζ Valence Quality for One- and Two-Component Relativistic All-Electron Calculations. *J. Chem. Theory Comput.* **2017**, *13* (8), 3696-3705. DOI: 10.1021/acs.jctc.7b00593.
(9) Franzke, Y. J.; Treß, R.; Pazdera, T. M.; Weigend, F. Error-consistent segmented contracted all-electron relativistic basis sets of double- and triple-zeta quality for NMR shielding constants. *Phys. Chem. Chem. Phys.* **2019**, *21* (30), 16658-16664, 10.1039/C9CP02382H. DOI: 10.1039/C9CP02382H.
(10) Garcia-Ratés, M.; Neese, F. Effect of the Solute Cavity on the Solvation Energy and its Derivatives within the Framework of the Gaussian Charge Scheme. *J. Comput. Chem.* **2020**, *41* (9), 922-939. DOI: https://doi.org/10.1002/jcc.26139.
(11) Grimme, S.; Antony, J.; Ehrlich, S.; Krieg, H. A consistent and accurate ab initio parametrization of density functional dispersion correction (DFT-D) for the 94 elements H-Pu. *J. Chem. Phys.* **2010**, *132* (15). DOI: 10.1063/1.3382344 (acccessed 5/13/2025).
(12) Noodleman, L. Valence bond description of antiferromagnetic coupling in transition metal dimers. *J. Chem. Phys.* **1981**, *74* (10), 5737-5743. DOI: 10.1063/1.440939 (acccessed 5/13/2025).
(13) Noodleman, L.; Norman, J. G., Jr.; Osborne, J. H.; Aizman, A.; Case, D. A. Models for ferredoxins: electronic structures of iron-sulfur clusters with one, two, and four iron atoms. *J. Am. Chem. Soc.* **1985**, *107* (12), 3418-3426. DOI: 10.1021/ja00298a004.
(14) Becke, A. D. Density-functional thermochemistry. III. The role of exact exchange. *J. Chem. Phys.* **1993**, *98* (7), 5648-5652. DOI: 10.1063/1.464913 (acccessed 5/13/2025).
(15) Lee, C.; Yang, W.; Parr, R. G. Development of the Colle-Salvetti correlation-energy formula into a functional of the electron density. *Phys. Rev. B* **1988**, *37* (2), 785-789. DOI: 10.1103/PhysRevB.37.785.
